# Supplementary material for: MAVS integrates glucose metabolism and RIG-I-like receptor signaling
Source: Nat Commun. 2023 Sep 2;14:5343. doi: 10.1038/s41467-023-41028-9 (PMC10475032; doi:10.1038/s41467-023-41028-9)

Supplementary Information

MAVS integrates glucose metabolism and RIG-I-like receptor signaling

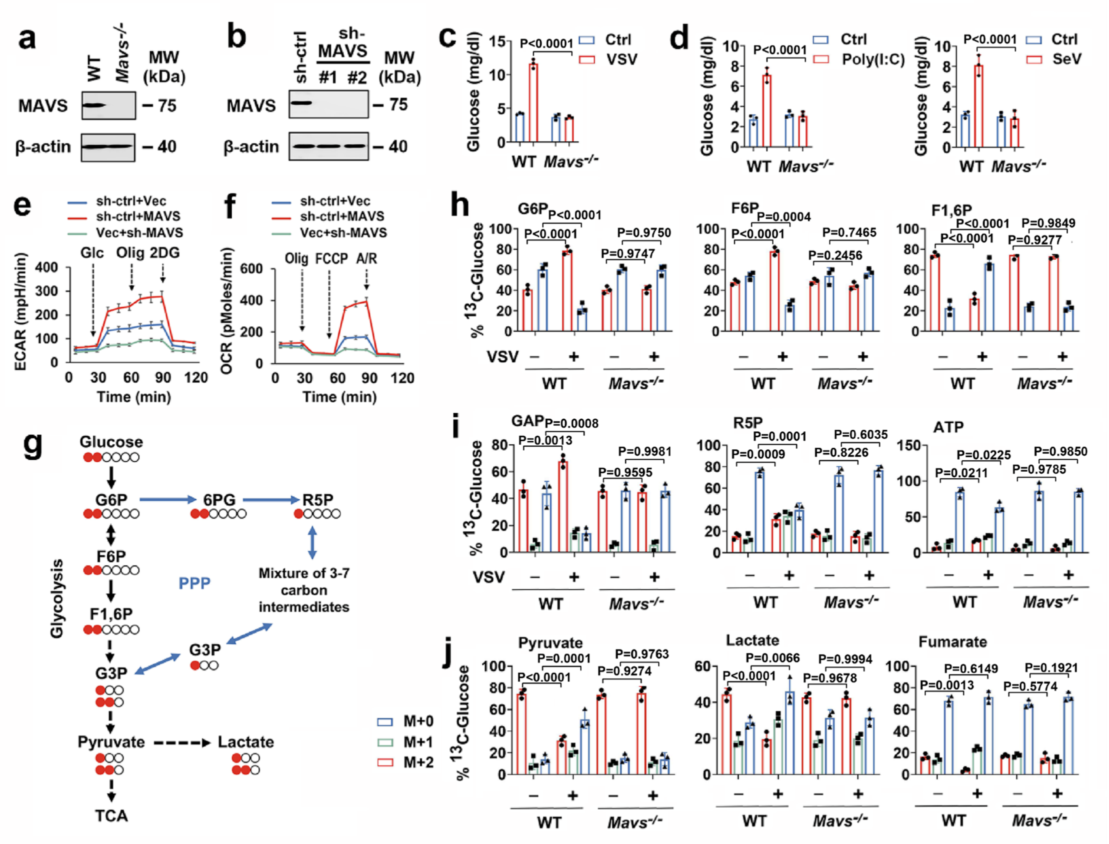

Supplementary Fig. 1. RLR activation shifts glucose flux from glycolysis to the PPP and the HBP via MAVS, Related to Fig 1.

(a) MAVS expression in BMDMs was measured using Western blot analyses. Experiments were repeated at least three times.

(b) HepG2 cells were transfected with shRNA-ctrl or indicated shRNA-MAVS for 48 h before Western blot assays. Experiments were repeated at least three times.

(c) *Mavs*-WT (WT) and *Mavs*$^{-/-}$ BMDMs were infected with or without VSV (MOI = 1) for 6 h before glucose levels measurements (n = 3 mice per condition, repeated measures two-way ANOVA, mean ± SEM).

(d) WT and *Mavs*$^{-/-}$ BMDMs were treated with or without poly(I:C) (1 μg/mL) for 12 h (left panel) or infected with or without SeV (MOI = 1) for 6 h (right panel) before glucose levels analyses. (n = 3 mice per condition, two-way ANOVA, mean ± SEM)

(e and f) HepG2 cells were transfected with control vector, pCMV-MAVS, shRNA-control (sh-ctrl), or shRNA-MAVS for indicated times before ECAR (e) or OCR (f) analyses in supernatants (n = 3 per condition, repeated measures two-way ANOVA,

mean ± SEM).
(g) Schematic of 1,2-^{13}C-glucose carbon labeling through glycolysis and PPP.
(h-j) WT and *Mavs*$^{-/-}$ BMDMs were infected with or without VSV (MOI = 1) for 6 h. 1,2-^{13}C-glucose incorporation into upper glycolytic metabolites, PPP, nucleotides, and TCA cycle were analyzed. (n = 3 mice per condition, two-way ANOVA, mean ± SEM). Source data are provided as a Source Data file.

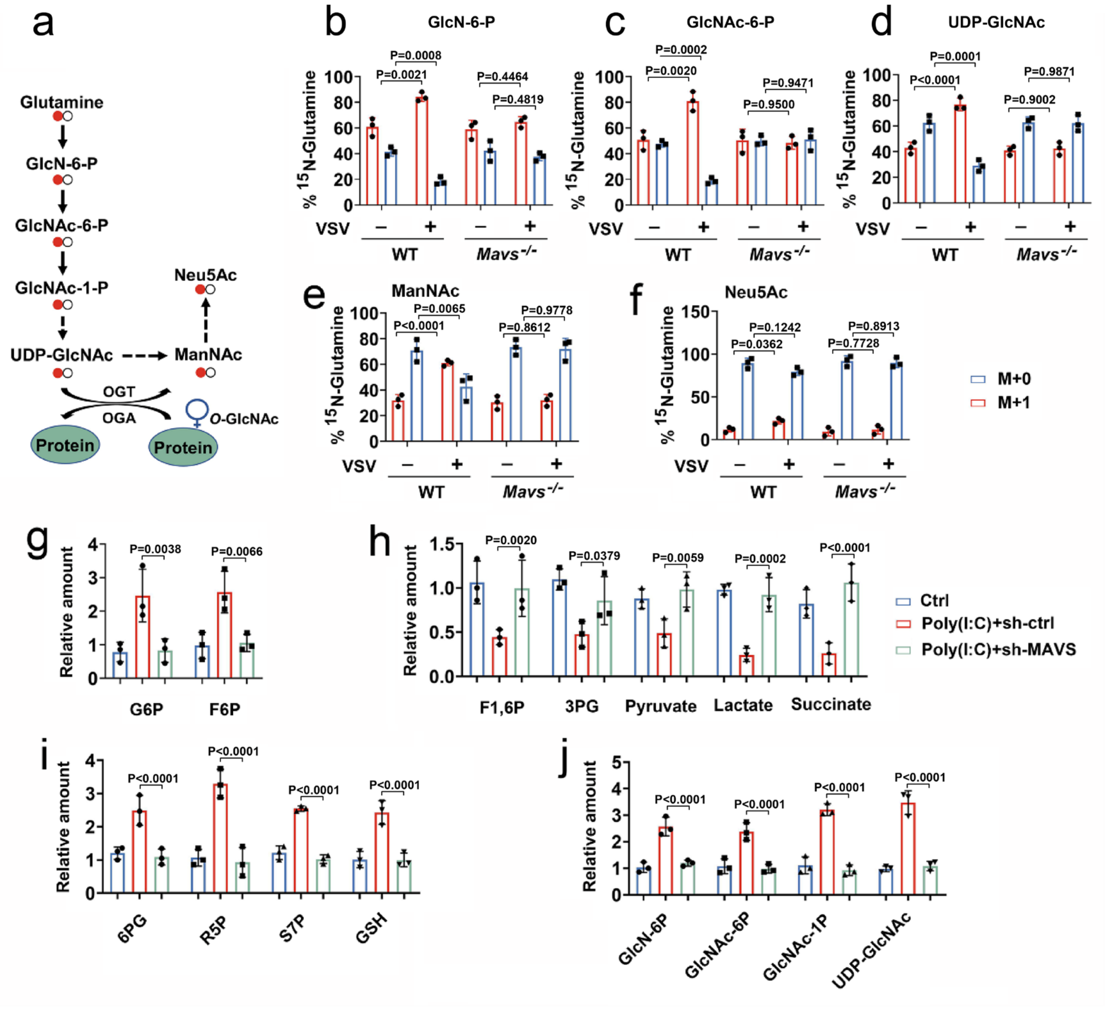

Supplementary Fig. 2. RLR activation shifts glucose flux from glycolysis to the PPP and the HBP via MAVS, Related to Fig 1.
(a) Schematic of the hexosamine biosynthesis pathway and ^{15}N incorporation from [γ-^{15}N] glutamine into the HBP intermediates.
(b-f) WT and *Mavs*$^{-/-}$ BMDMs were cultured [γ-^{15}N] glutamine-containing media and infected with or without VSV (MOI = 1) for 6 h. Metabolites were extracted and subjected to LC-MS.
(g-j) THP-1 cells were stimulated or unstimulated (Med) for 12 h with poly(I:C) (50 µg/ml) metabolites quantified by metabolomics.
All experiments in (g-j) were repeated at least three times. Data in (b)-(f) represent two-way ANOVA as means ± SEMs, n = 3 mice per condition. Data in (g)-(j) are presented

in the means ± SD, two-sided Student's t-test. Source data are provided as a Source Data file.

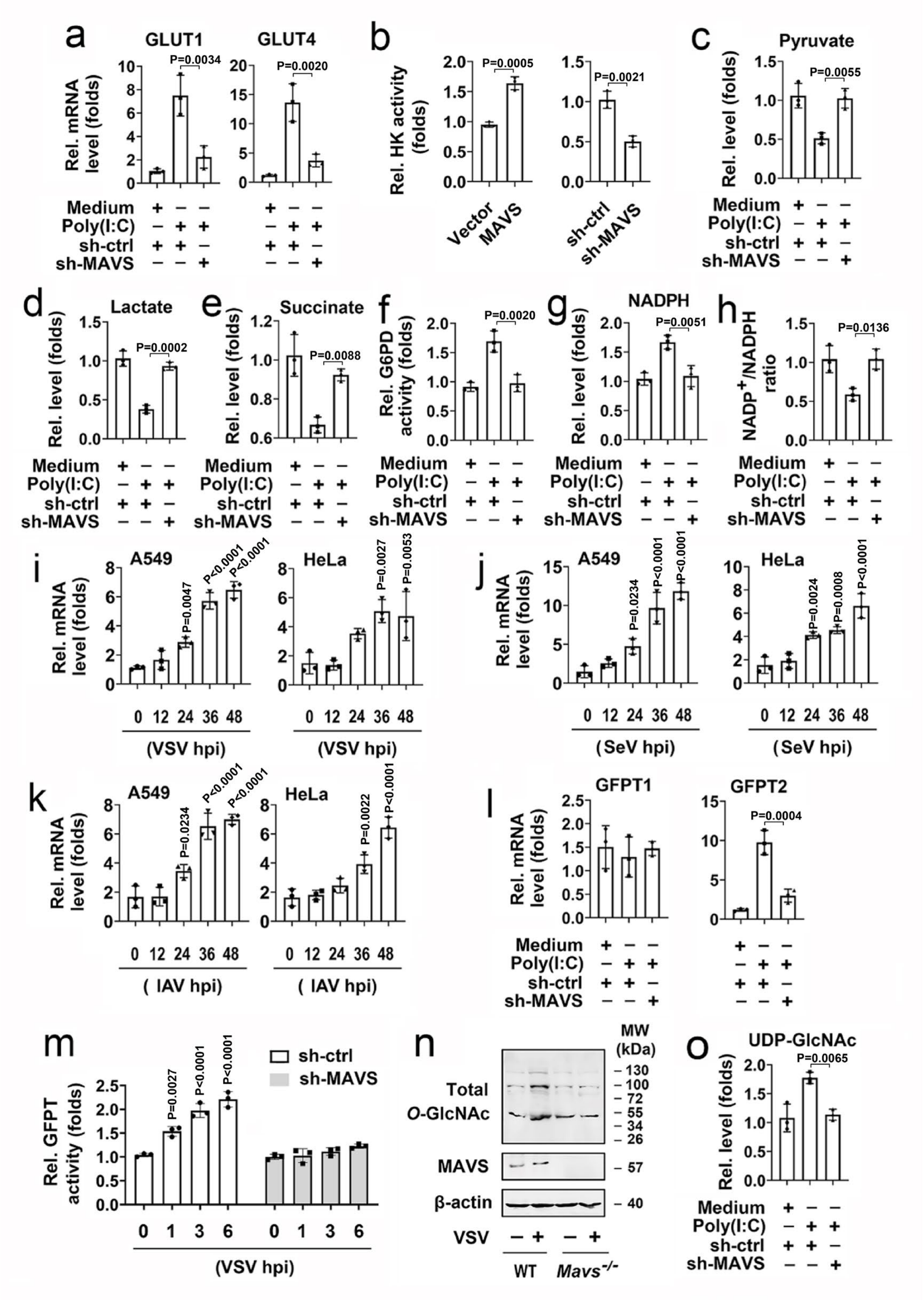

Supplementary Fig. 3. RLR activation alters intermediates of glucose metabolism via MAVS, Related to Fig 2.

(a) THP-1 cells were transfected with sh-ctrl or sh-MAVS for 36 h and stimulated or unstimulated (Med) for 12 h with poly(I:C) (50 μg/ml) before qPCR.

(b) THP-1 cells were transfected with control vector or pCMV-MAVS (left panel), sh-ctrl, or sh-MAVS (right panel) for 36 h, followed by measurement of HK activity.

(c-h) THP-1 cells were transfected with sh-ctrl or sh-MAVS for 36 h and stimulated or unstimulated (Med) for 12 h with poly(I:C) (50 μg/ml), followed by measuring total pyruvate levels (c), lactate levels (d), succinate levels (e), G6PD activity (f), NADPH (g), and $NADP^+$/NADPH ratio (h) levels

(i) A549 cells (left panel) and HeLa cells (right panel) were infected with VSV (MOI = 1) for indicated times. GFPT2 RNA levels were quantified by qPCR.

(j and k) Experiments were performed similar to those in (i), except SeV (MOI = 1) (j) or IAV (MOI = 1) (k) were used.

(l) THP-1 cells were transfected with sh-ctrl or sh-MAVS for 36 h and stimulated or unstimulated (Med) for 12 h with poly(I:C) (50 μg/ml). GFPT1 (left panel) or GFPT2 (right panel) RNA levels were quantified by qPCR.

(m) THP-1 cells were transfected with sh-ctrl or sh-MAVS for 36 h and were infected with VSV (MOI = 1) for indicated times, followed by measuring GFPT activity.

(n) WT and *Mavs*$^{-/-}$ BMDMs were infected with or without VSV (MOI = 1) for 6 h, followed by measuring total *O*-GlcNAc levels. Experiments were repeated at least three times.

(o) THP-1 cells were transfected with sh-ctrl or sh-MAVS for 36 h and stimulated or unstimulated (Med) for 12 h with poly(I:C) (50 μg/ml), followed by measuring UDP-GlcNAc levels.

All data are presented as means ± SD, n = 3 per condition, two-sided Student's t-test. Source data are provided as a Source Data file.

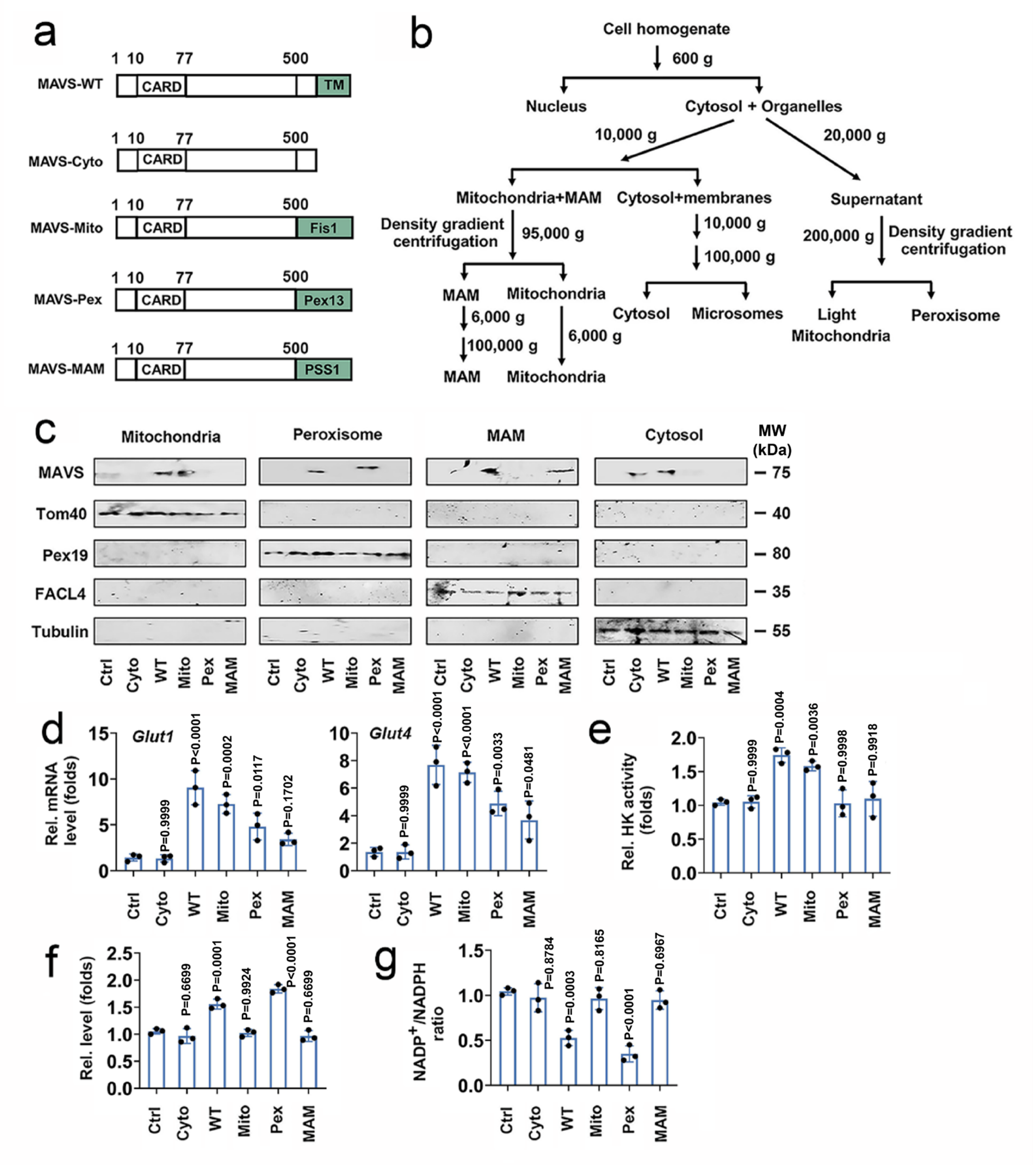

Supplementary Fig. 4. MAVS subcellular localization is critical for glucose metabolism reprogramming, Related to Fig 3.

(a) Schematic of WT and mutant MAVS alleles to be tested for signaling from peroxisomes, mitochondria, and MAM.

(b) Cell fractionation strategy.

(c) *Mavs*$^{-/-}$ BMDMs were transfected with the control vector or indicated MAVS alleles for 48 h. Subcellular fractions were isolated for immunoblot analysis. Fractionation markers: mitochondria (Tom40); MAM (FACL4); peroxisomes (Pex19); cytosol (Tubulin). Experiments were repeated at least three times.

(d and e) *Mavs*$^{-/-}$ BMDMs were transfected with the control vector or indicated MAVS alleles for 24 h, followed by measurement of GLUT1 and GLUT4 mRNA levels (d), or mitochondria HK activity (e) (n = 3 mice per condition, one-way ANOVA).

(f and g) *Mavs*$^{-/-}$ BMDMs were transfected with the control vector or indicated MAVS

alleles for 24 h, followed by an analysis of NADPH (f) and $NADP^+$/NADPH (g) ratio levels (n = 3 mice per condition, one-way ANOVA).

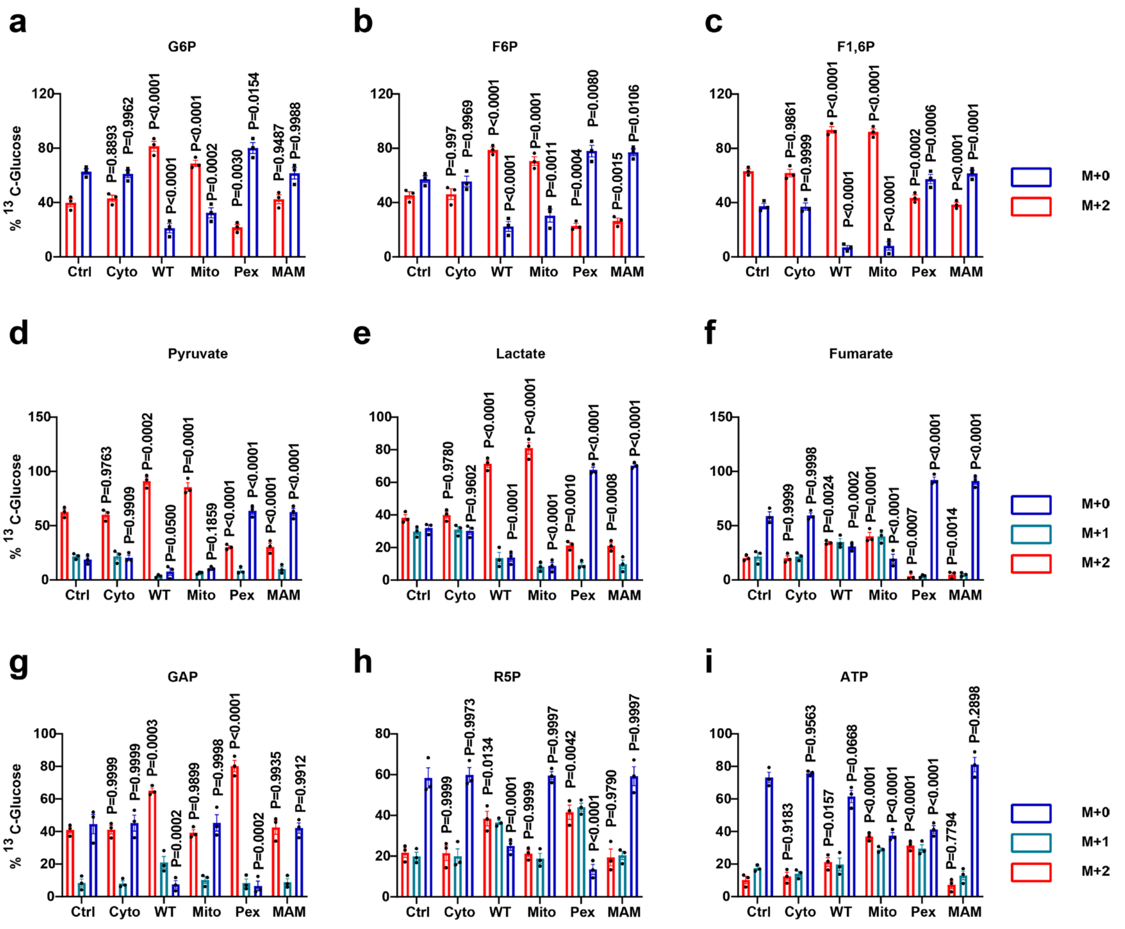

Supplementary Fig. 5. MAVS subcellular localization is critical for glucose metabolism reprogramming, Related to Fig 3.

(a-i) *Mavs*$^{-/-}$ BMDMs were transfected with a control vector or indicated MAVS alleles for 24 h. 1,2-^{13}C-glucose incorporation into glycolytic metabolites (a-d), TCA cycle (e and f), PPP (g and h), and nucleotides (i) were analyzed (n = 3 mice per condition, one-way ANOVA, mean ± SEM). Source data are provided as a Source Data file.

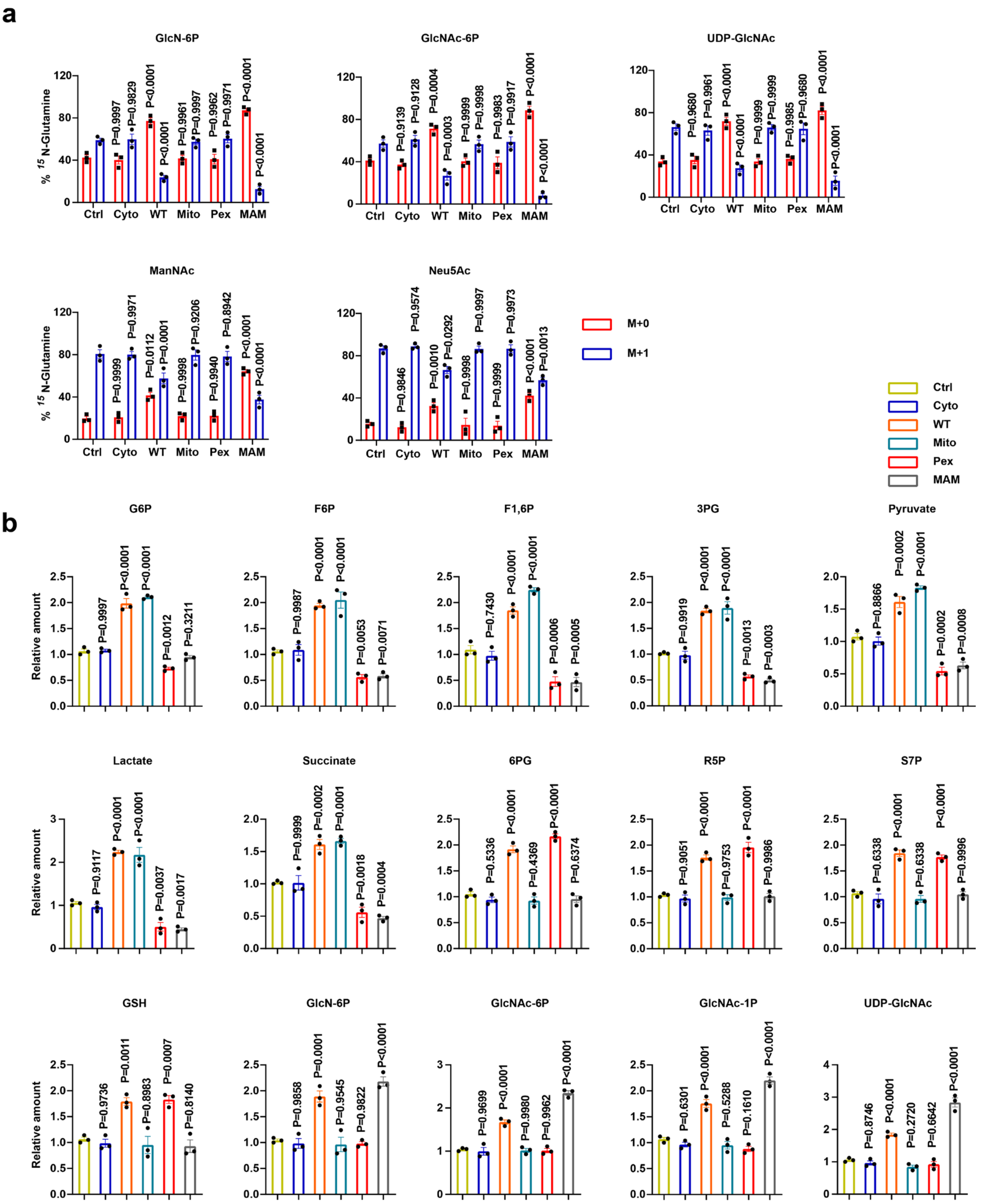

Supplementary Fig. 6. MAVS subcellular localization is critical for glucose metabolism reprogramming, Related to Fig 3.

(a) *Mavs*$^{-/-}$ BMDMs were transfected with a control vector or indicated MAVS alleles for 24 h and cultured with [γ-^{15}N] glutamine-containing media for 6 h. Metabolites were extracted and subjected to LC-MS.

(b) *Mavs*$^{-/-}$ BMDMs were transfected with control vector or indicated MAVS alleles for 36 h, metabolites quantified by metabolomics.

All data are presented as means ± SEMs, n = 3 mice per condition, one-way ANOVA. Source data are provided as a Source Data file.

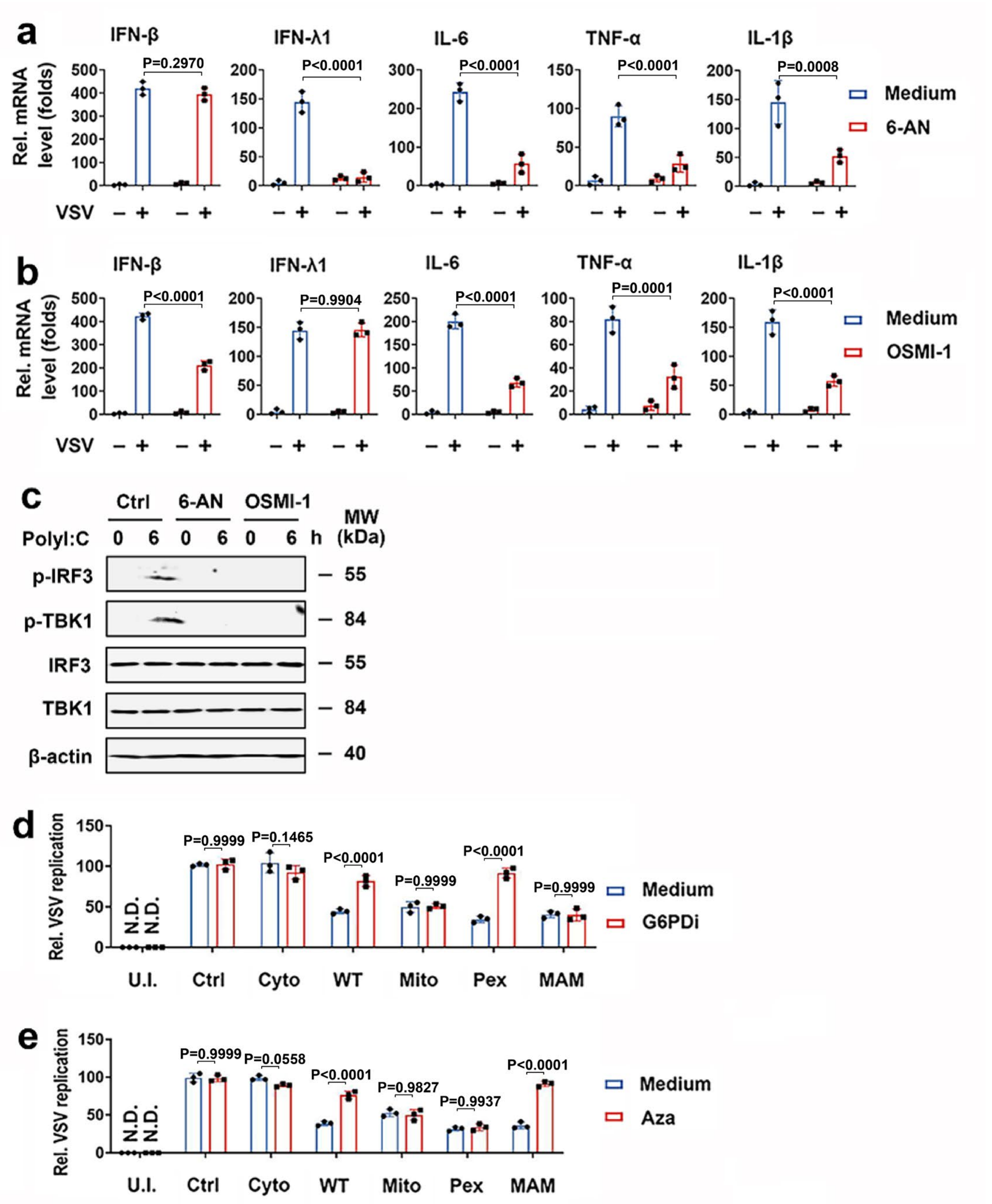

Supplementary Fig. 7. the PPP and the HBP regulate antiviral immune responses in response to RLR activation, Related to Fig 4.

(a) THP-1 cells were infected with or without VSV (MOI = 1) for 6 h and treated with or without 6-AN (1 mM for 4 h) before qPCR analyses.

(b) Experiments were performed as described in (a), except that OSMI-1 (20 μM for 2 h) were used.

(c) THP-1 cells were treated with or without polyI:C for 3 h. Then, cells were treated with G6DPi 6-AN (1 mM for 3 h) or OSMI-1 (20 μM for 3 h), followed by immunoblotting with the indicated antibodies. Experiments were repeated at least three times.

(d and e) *Mavs*$^{-/-}$ BMDMs were transfected with the control vector or indicated MAVS alleles for 24 h and infected with or without VSV (MOI = 1) for 12 h. Then, cells were

treated with or without G6DPi (50 μM for 4 h) (d) or Aza (0.5 mM for 6 h) (e) before viral titers were measured using a plaque assay.

Data in (a) and (b) are repeated at least three times and presented as means ± SD, two-way ANOVA.

Data in (d) and (e) are expressed as means ± SEMs, n = 3 mice per condition, two-way ANOVA. Source data are provided as a Source Data file.

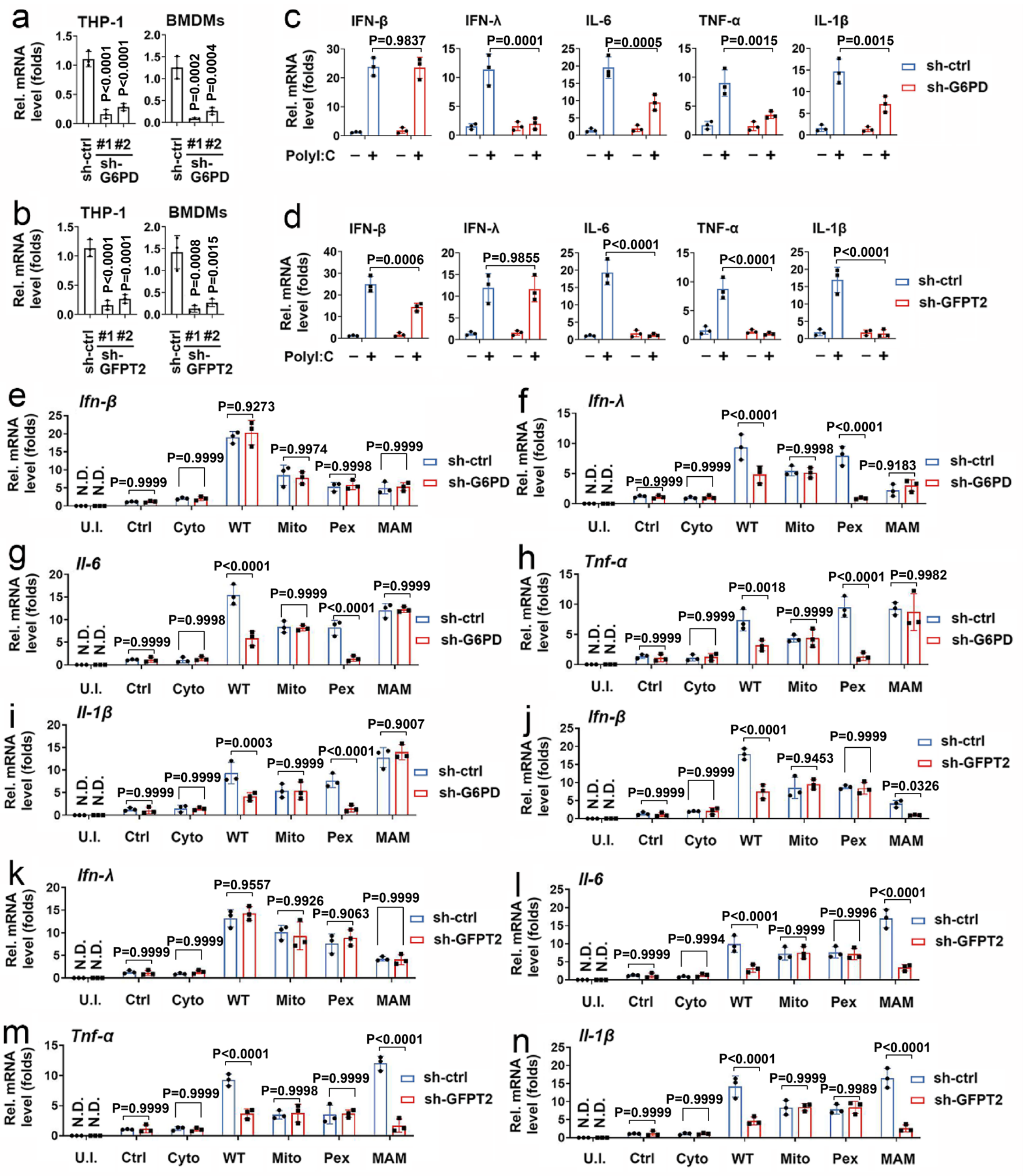

Supplementary Fig. 8. the PPP and the HBP regulate antiviral immune responses in response to RLR activation, Related to Fig 4.

(a) THP-1 cells (left panel) or BMDMs (right panel) were transfected with sh-ctrl or

sh-G6PD for 48 h before qPCR.

(b) Experiments were performed similar to those in (a), except sh-GFPT2 were used.

(c and d) THP-1 cells were transfected with sh-ctrl, sh-G6PD (c), or sh-GFPT2 (d) for 36 h and stimulated or unstimulated (Med) for 12 h with poly(I:C) (50 µg/ml) before qPCR.

(e-i) *Mavs*$^{-/-}$ BMDMs were transfected with sh-ctrl, sh-G6PD, or indicated MAVS alleles for 48 h before qPCR.

(j-n) Experiments were performed as described in (e-i), except that sh-GFPT2 was used. Data in (a)-(d) were repeated at least three times and presented as means ± SD, two-sided Student's t-test. Data in (e)-(n) are presented as means ± SEMs, n = 3 mice per condition, two-way ANOVA. Source data are provided as a Source Data file.

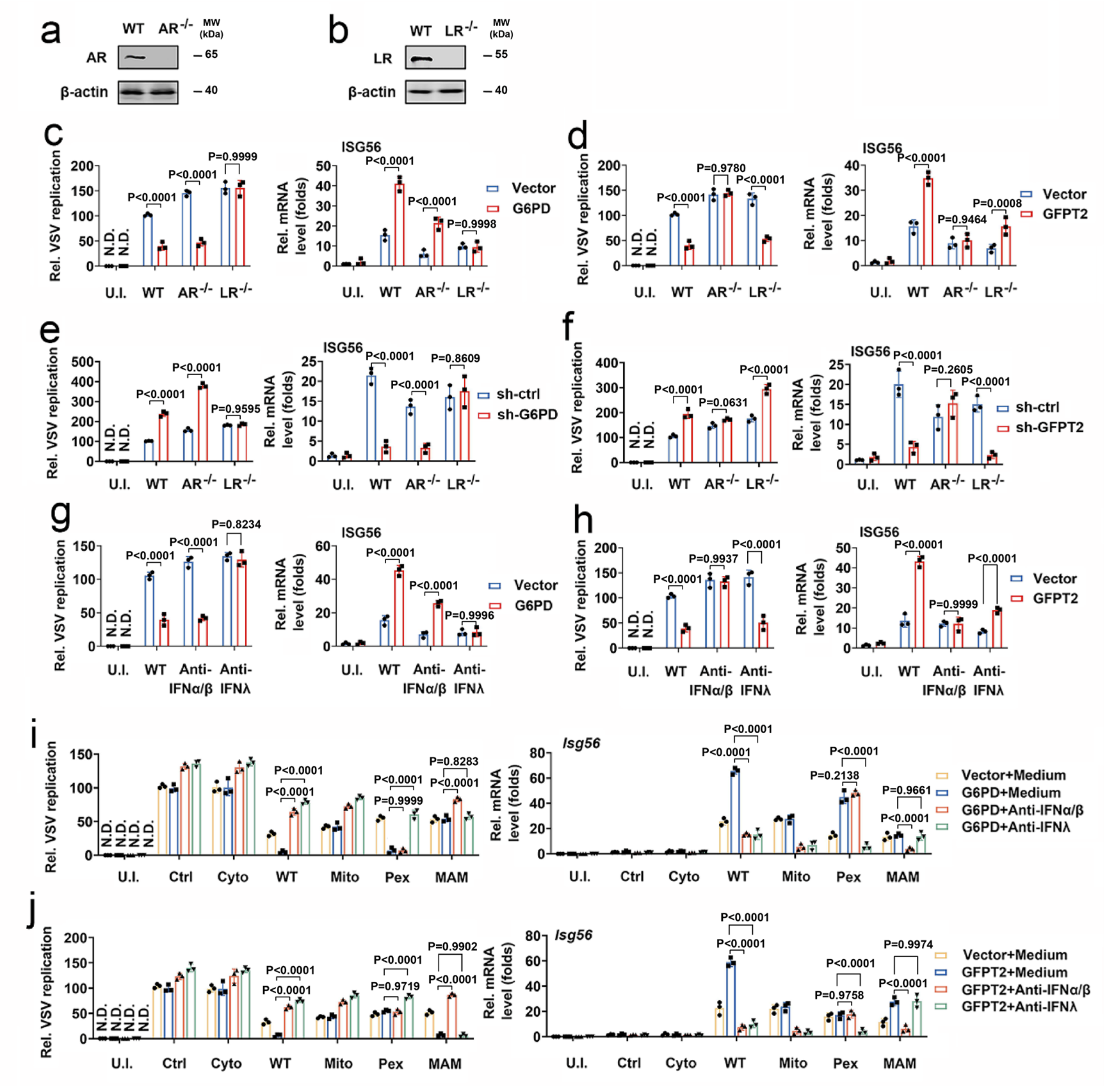

Supplementary Fig. 9. The PPP and the HBP regulate antiviral immune responses in response to RLR activation, Related to Fig 4.

(a and b) IFNAR1 expression in IFNAR1 WT (WT) and IFNAR1$^{-/-}$ (AR$^{-/-}$) cells (a),

and IFNLR expression in IFNLR WT (WT) and IFNLR$^{-/-}$ (LR$^{-/-}$) cells (b) were measured using Western blot. Experiments were repeated at least three times.
(c) WT and AR$^{-/-}$ cells were transfected with control vector or pCMV-G6PD for 36 h and infected with or without VSV (MOI = 1) for 12 h, followed by an analysis of viral titers (left panel) or ISG56 RNA levels (right panel).
(d) Experiments were performed as described in (c), except that pCMV-GFPT2 were transfected.
(e and f) Experiments were performed as described in (c and d), except that sh-G6PD (e) or sh-GFPT2 (f) were transfected.
(g) A549 cells were transfected with a control vector or pCMV-G6PD for 36 h and were then infected with VSV (MOI = 1) and incubated with neutralizing antibodies against IFNα/β (each at 2 μg/mL) or IFNλ (1 μg/mL) for 12 h, followed by an analysis of viral titers (left panel) or ISG56 RNA levels (right panel).
(h) Experiments were performed as described in (g), except that pCMV-GFPT2 were transfected.
(i) *Mavs*$^{-/-}$ BMDMs were transfected with the control vector, pCMV-G6PD, or indicated MAVS alleles for 36 h and were then infected with VSV (MOI = 1) and incubated with neutralizing antibodies against IFNα/β or IFNλ for 12 h, followed by an analysis of viral titers (left panel) or ISG56 RNA levels (right panel).
(j) Experiments were performed as described in (i), except that pCMV-GFPT2 were transfected.
Data in (c)-(h) are repeated at least three times and presented as means ± SD, two-sided Student's t-test. Data in (i) and (j) are presented as means ± SEMs, n = 3 mice per condition, two-way ANOVA. Source data are provided as a Source Data file.

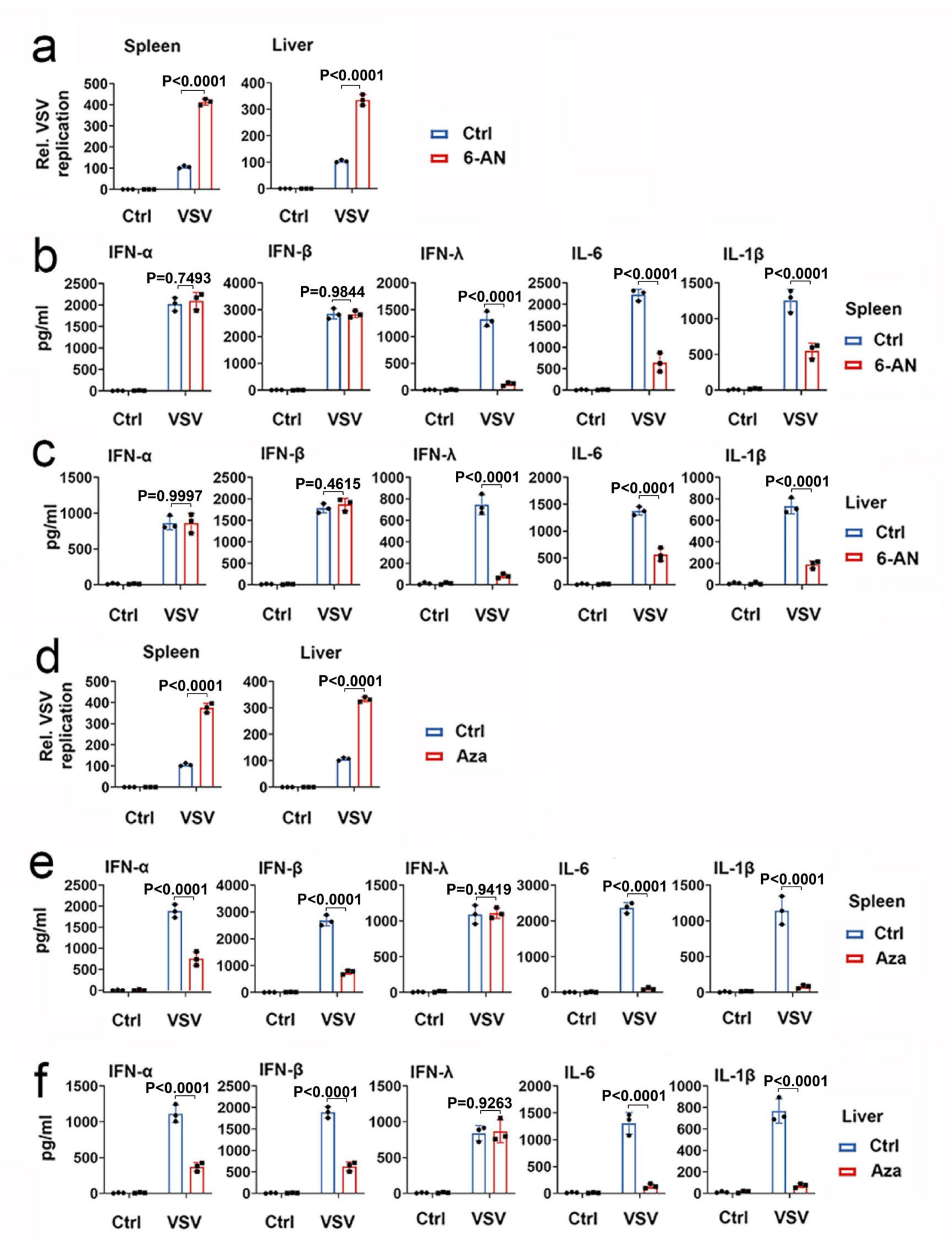

Supplementary Fig. 10. The PPP and the HBP are critical for the activation of antiviral immune signaling *in vivo*, Related to Fig 5.

(a) C57BL/6 mice were treated with PBS or 6-AN (4 mg/kg per day) for 24 h and infected with VSV (2×10^7 pfu/g) for 24 h, followed by an analysis of VSV RNA in the spleen and liver.

(b and c) C57BL/6 mice were treated with PBS or 6-AN (4 mg/kg per day) for 24 h and infected with VSV (2×10^7 pfu/g) for 24 h, followed by an analysis of levels of proinflammatory cytokines and IFN in the spleen (b) and liver (c).

(d-f) Experiments were performed as described in (a-c), except that azaserine (Aza) (2.5 mg/kg per day) was used. Data are presented as means ± SEMs, n = 3 mice per condition, two-way ANOVA. Source data are provided as a Source Data file.

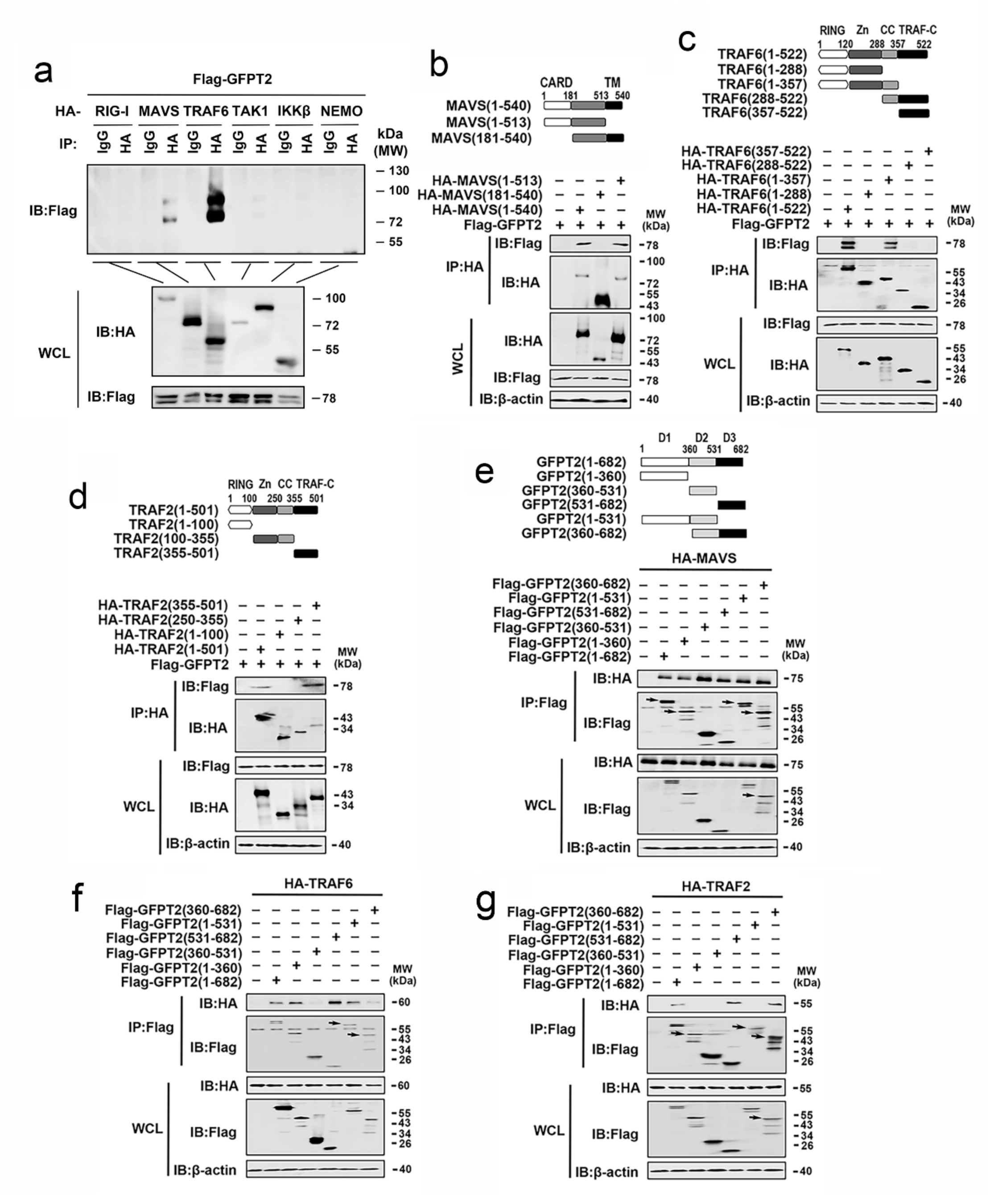

Supplementary Fig. 11. GFPT2 associates with MAVS/TRAF2/TRAF6 complex, Related to Fig 6.

(a) HEK293 cells were transfected with indicated plasmids for 48 h. Co-IP and immunoblot analyses were performed with the indicated antibodies.

(b) Schematic diagram of the full-length and truncated constructs of MAVS (upper panel). HEK293 cells were co-transfected with Flag-GFPT2 and the indicated truncated

MAVS constructs for 48 h. Co-IP and immunoblot analyses were performed with the indicated antibodies (lower panel).
(c and d) Experiments were performed similar to those in (b), except the indicated truncated constructs of TRAF6 (c) or TRAF2 (d) were used.
(e) Schematic diagram of the full-length and truncated constructs of GFPT2 (upper panel). HEK293 cells were co-transfected with HA-MAVS and the indicated truncated GFPT2 constructs for 48 h. Co-IP and immunoblot analyses were performed with the indicated antibodies (lower panel).
(f and g) Experiments were performed similar to those in (e), except the HA-TRAF6 (f) or HA-TRAF2 (g) were used.
All experiments were repeated at least three times. Source data are provided as a Source Data file.

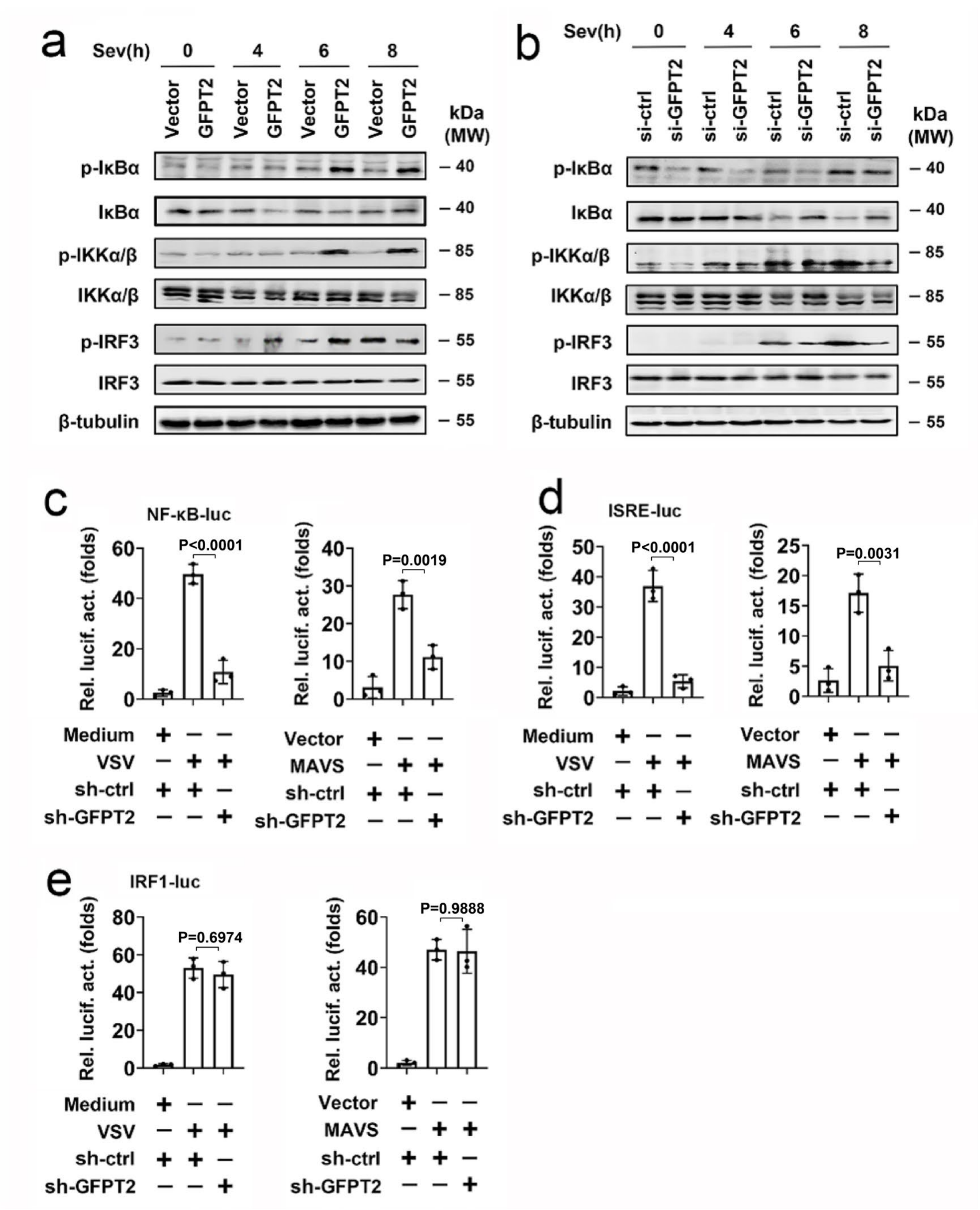

Supplementary Fig. 12. GFPT2 associates with MAVS/TRAF2/TRAF6 complex, Related to Fig 6.

(a and b) A549 cells were transfected with vector or GFPT2 expression plasmid for 24 h (a), sh-ctrl, or sh-GFPT2 for 36 h (b). The cells were then infected with SeV (MOI = 1) for the indicated times, followed by immunoblotting with the indicated antibodies. Experiments were repeated at least three times.

(c) A549 cells were transfected with NF-κB-luc, sh-ctrl, or sh-GFPT2 for 36 h and infected with VSV (MOI = 1) for 12 h before luciferase assays.

(d and e) Experiments were performed similar to those in (c), except ISRE-luc (d) or IRF1-luc (e) were used.

Data in (c)-(e) are repeated at least three times and presented as means ± SD, two-sided

Student’s t-test. Source data are provided as a Source Data file.

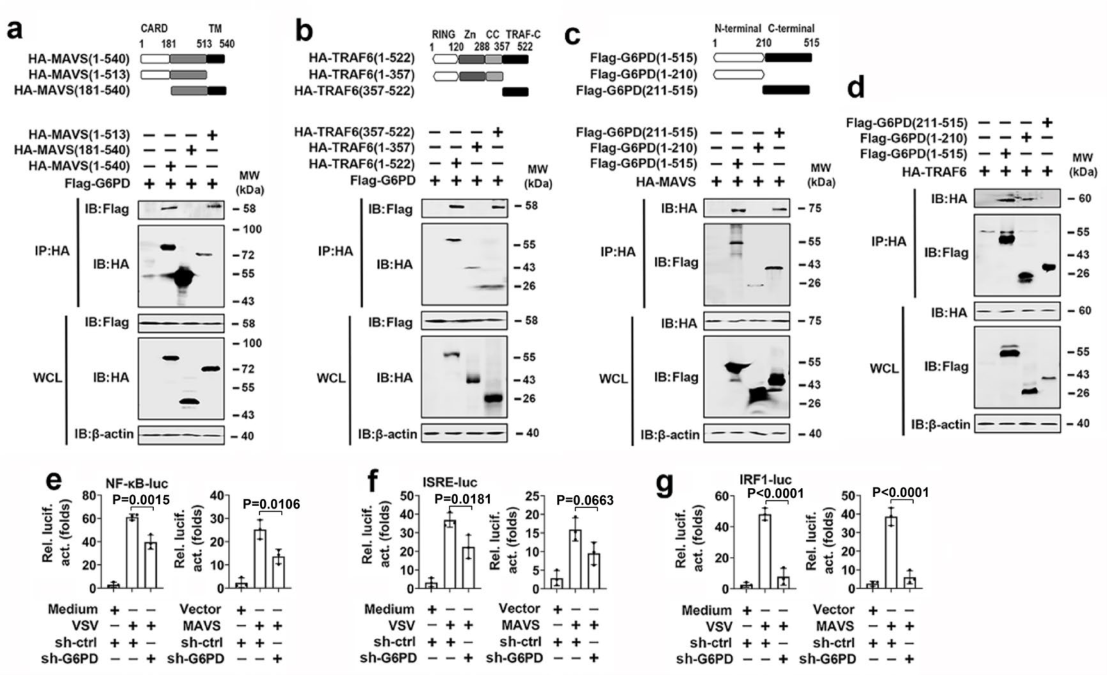

Supplementary Fig. 13. G6PD associates with MAVS/TRAF6 complex, Related to Fig 7.

(a) Schematic diagram of the full-length and truncated constructs of MAVS (upper panel). HEK293 cells were co-transfected with Flag-G6PD and the indicated truncated MAVS constructs for 48 h. Co-IP and immunoblot analyses were performed with the indicated antibodies (lower panel). Experiments were repeated at least three times.

(b-d) Experiments were performed similar to those in (a), except the indicated truncated constructs of TRAF6 (b) and G6PD (c and d) were used. Experiments were repeated at least three times.

(e) A549 cells were transfected with NF-κB-luc, sh-ctrl, or sh-G6PD for 36 h and infected with VSV (MOI = 1) for 12 h before luciferase assays.

(f and g) Experiments were performed similar to those in (e), except ISRE-luc (f) or IRF1-luc (g) were used.

Data in (e)-(g) are repeated at least three times and presented as means ± SD, two-sided Student’s t-test. Source data are provided as a Source Data file.

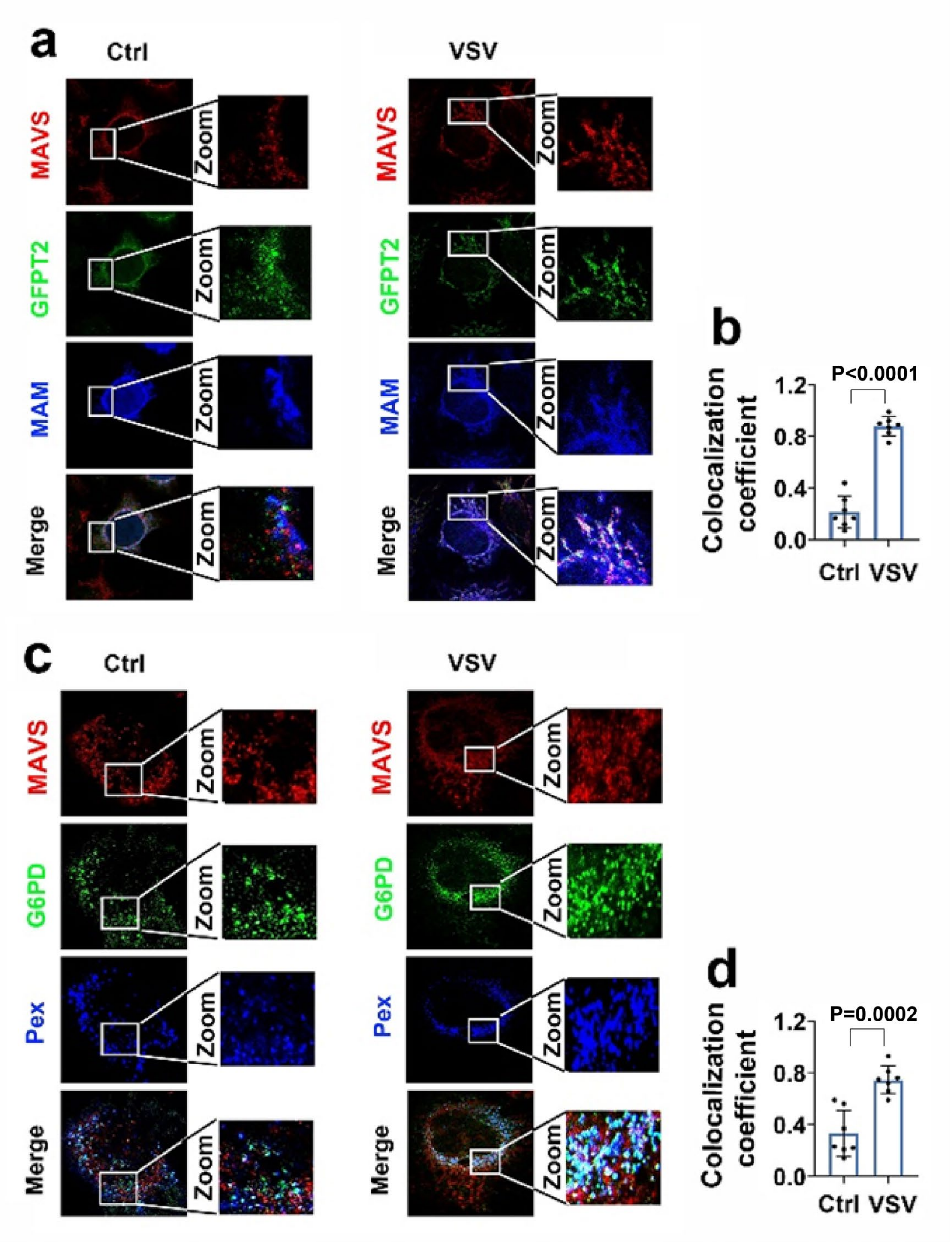

Supplementary Fig. 14. Fig 8. The specific subcellular localization of MAVS regulates different signaling pathways via the recruitment of GFPT2/TRAF6/TRAF2 or G6PD/TRAF6/IRF1, Related to Fig 8.

(a and b) Hela cells were transfected with Organelle-labeled plasmids (Turquoise2-MAM) for 24 hours, and infected with or without VSV (MOI=1) for 12 hours to conduct confocal microscopy assays (a). The colocalization of MAVS, GFPT2 and MAM was quantitated (b).

(c and d) Experiments were performed similar to those in (a) and (b), except

Turquoise2-Pex was used.

Data in (b, d) are presented as means ± SD, two-sided Student’s t-test. Single-cell images are representative of three experiments and 7 cells analyzed per experiment. Source data are provided as a Source Data file.

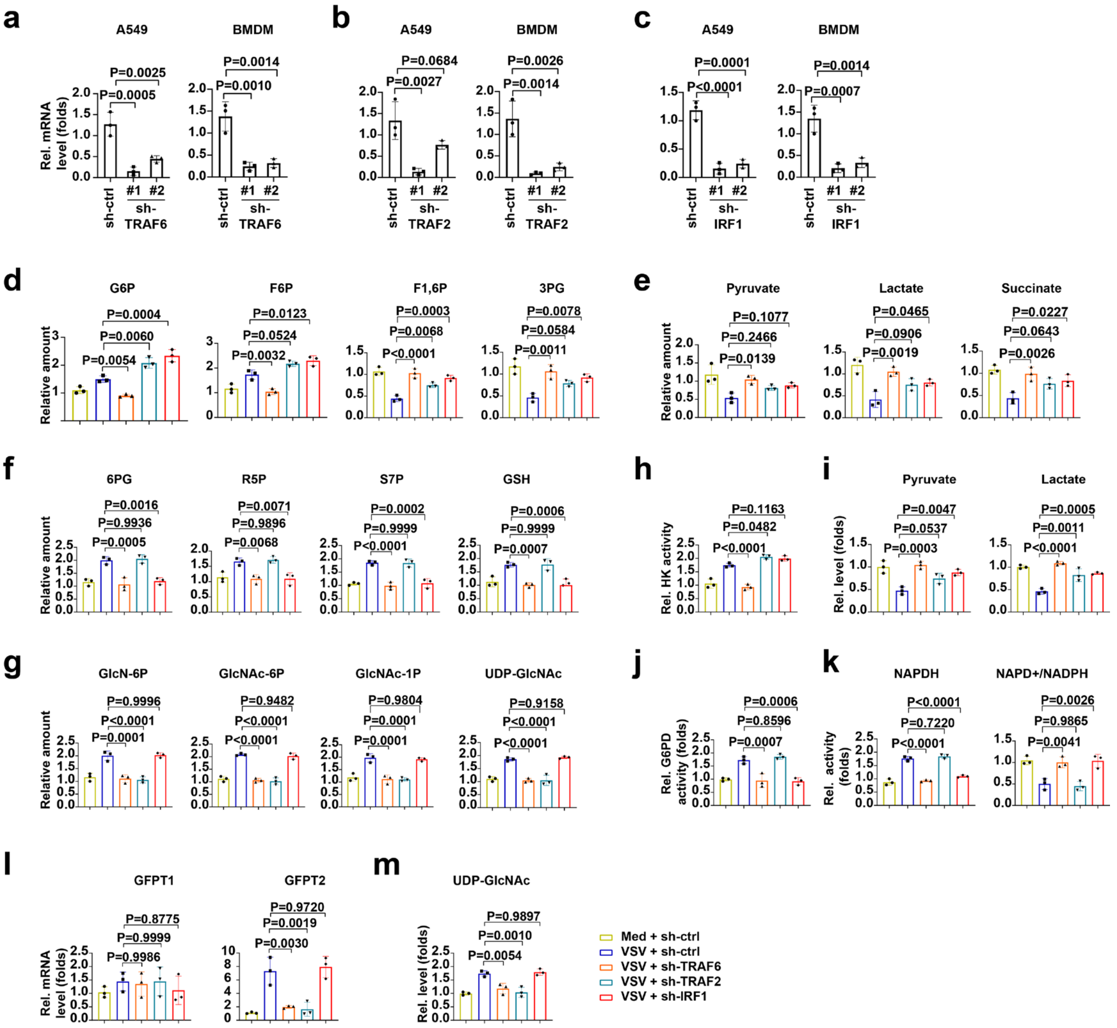

Supplementary Fig. 15. TRAF6, TRAF2, and IRF1's role in VSV-regulated glucose metabolism reprogramming.

(a) A549 cells (left panel) or BMDMs (right panel) were transfected with sh-ctrl or sh-TRAF6 for 48 h before qPCR.

(b and c) Experiments were performed similar to those in (a), except sh-TRAF2 (b) or sh-IRF1 (c) were used.

(d-g) A549 cells were transfected with sh-ctrl or indicated shRNAs for 36 h and infected with VSV (MOI = 1) for 6 h. Metabolites were quantified by metabolomics.

(h-k) A549 cells were transfected with sh-ctrl or indicated shRNAs for 36 h, infected with VSV (MOI = 1) for 6 h, followed by analysis of mitochondria HK activity (h), total pyruvate and lactate levels (i), G6PD activity (j), NADPH and $NADP^{+}$/NADPH ratio levels (k).

(l and m) A549 cells were transfected with sh-ctrl or indicated shRNAs for 36 h,

infected with VSV (MOI = 1) for 6 h, followed by analysis of GFPT1 and GFPT2 mRNA levels (l), and UDP-GlcNAc levels (m).
All data are repeated at least three times and presented as means ± SEM, two-sided Student's t-test. Source data are provided as a Source Data file.

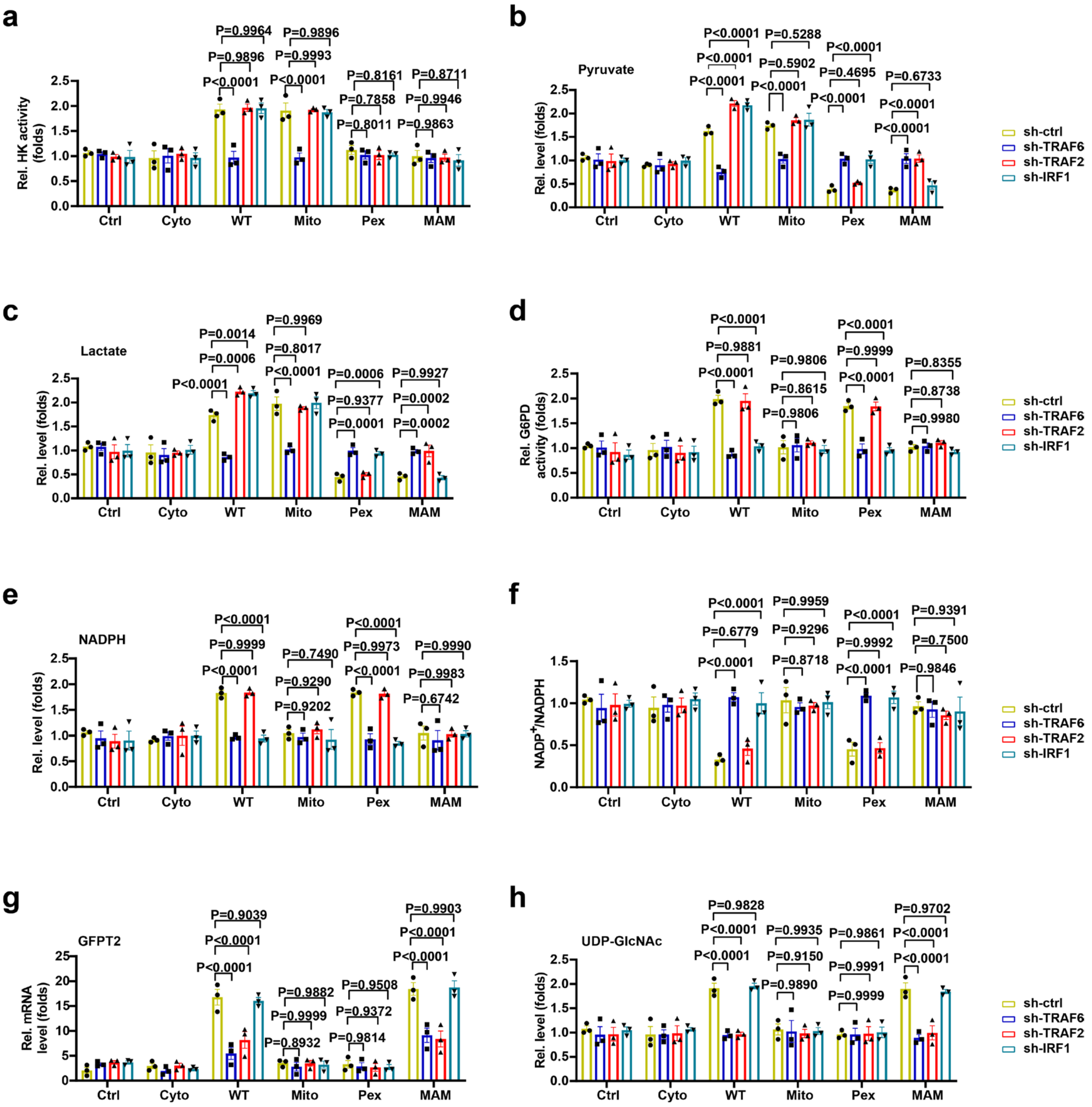

Supplementary Fig. 16. TRAF6/IRF1 complex mediates peroxisome-localized MAVS-induction of the PPP, while TRAF6/TRAF2 complex mediates MAMs-localized MAVS-induction of the HBP.

(a-h) WT and *Mavs*$^{-/-}$ BMDMs were transfected with control vector or indicated MAVS alleles for 36 h and infected with or without VSV (MOI = 1) for 6 h, followed by measurement of mitochondria HK activity (a), total pyruvate (b), lactate levels (c), G6PD activity (d), NADPH (e), NADP$^+$/NADPH ratio levels (f), GFPT2 mRNA level (g), and UDP-GlcNAc levels (h).
All data are presented as means ± SEMs, n = 3 mice per condition, two-way ANOVA. Source data are provided as a Source Data file.

Supplementary Table 1. Primers used in qPCR

Gene name	5'primer	3'primer
Glut1 (m)	5'-TCATTGTCGGCCTCCTCATT-3'	5'-TAGGGTGGCAGAACTTGAGG-3'
Glut4 (m)	5'-GCCATCGTCATTGGCATTCT-3'	5'-CTCCAGGTTCCGGATGATGT-3'
Gfpt1 (m)	5'-GAGACAGATTGCGGGGTTGA-3'	5'-CGGCAGTCGCTTCAGTCC-3'
Gfpt2 (m)	5'-GTATGATTGGCCGACCCTGG-3'	5'-ATGCTAGCCGGAGAGCTGAA-3'
IFN-β (h)	5'-AAAGAAGCAGCAATTTTCAGC-3'	5'-CCTTGGCCTTCAGGTAATGCA-3'
TNF-α (h)	5'-CTTCTCGAACCCCGAGTGAC-3'	5'- ATGAGGTACAGGCCCTCTGA-3'
IL-6 (h)	5'-TGGTGGATGTTCCCCCCGAG-3'	5'-TCCTGGGAATACTGGCACGG-3
TNF-α(m)	5'-GTCAGGTTGCCTCTGTCTCA-3'	5'- TCAGGGAAGAGTCTGGAAAG-3'
IL-6 (m)	5'-AGCCCACCAAGAACGATAGTCAA-3'	5'-TCATTTCCACGATTTCCCAGA-3'
IFN-β (m)	5'- CGTGGGAGATGTCCTCAACT-3'	5'- AGATCTCTGCTCGGACCACC-3'
GAPDH (h)	5'-GGAAGGTGAAGGTCGGAGTCA -3'	5'-CTCGCTCCTGGAAGATGGTGATGGG-3'
β-actin (m)	5'- GATCATTGCTCCTCCTGAGC -3'	5'- ACATCTGCTGGAAGGTGGAC -3'
IL-1β (m)	5'- TCGGACCCATATGAGCTGA-3'	5'- CCACAGGTATTTTGTCGTTGC-3'
IL-1β (h)	5'-CAGAAGTACCTGAGCTCGCC-3'	5'-CATGGCCACAACAACTGACG-3'
GFPT1 (h)	5'-GGATATGATTCTGCTGGTGTG-3'	5'-CCAACGGGTATGAGCTATTC-3'
GFPT2 (h)	5'-CAGTTGGAAGGTGCATTCGC-3'	5'-GGTCTGGATGGCTCGAGATG-3'
IFN-λ1(h)	5'-CTTCCAAGCCCACCCCAACT-3'	5'-GGCCTCCAGGACCTTCAGC-3'
IFN-λ2/3 (m)	5'-AGCTGCAGGCCTTCAAAAAG-3'	5'-TGGGAGTGAATGTGGCTCAG-3'

Abbreviations: H, human; m, mouse

Supplementary Table 2. shRNAs used in this study

Oligonucleotides	Source	Identifier
H: Sh-MAVS#1	Sigma-Aldrich	Cat# TRCN0000236029
H: Sh-MAVS#2	Sigma-Aldrich	Cat# TRCN0000236030
H: Sh-G6PD#1	Sigma-Aldrich	Cat# TRCN0000025817
H: Sh-G6PD #2	Sigma-Aldrich	Cat# TRCN0000025843
M: Sh-G6PD#1	Sigma-Aldrich	Cat# TRCN0000041430
M: Sh-G6PD #2	Sigma-Aldrich	Cat# TRCN0000041443
H: Sh-GFPT2#1	Sigma-Aldrich	Cat# TRCN0000075223
H: Sh-GFPT2#2	Sigma-Aldrich	Cat# TRCN0000075226
M: Sh-GFPT2#1	Sigma-Aldrich	Cat# TRCN0000031676
M: Sh-GFPT2#2	Sigma-Aldrich	Cat# TRCN0000031677
H: Sh-TRAF6#1	Sigma-Aldrich	Cat# TRCN0000007348
H: Sh-TRAF6#2	Sigma-Aldrich	Cat# TRCN0000007349
M: Sh-TRAF6#1	Sigma-Aldrich	Cat# TRCN0000040735
M: Sh-TRAF6#2	Sigma-Aldrich	Cat# TRCN0000040736
H: Sh-TRAF2#1	Sigma-Aldrich	Cat# TRCN0000004573
H: Sh-TRAF2#2	Sigma-Aldrich	Cat# TRCN0000004574
M: Sh-TRAF2#1	Sigma-Aldrich	Cat# TRCN0000077229
M: Sh-TRAF2#2	Sigma-Aldrich	Cat# TRCN0000077232
H: Sh-IRF1#1	Sigma-Aldrich	Cat# TRCN0000014671

H: Sh-IRF1#2	Sigma-Aldrich	Cat# TRCN0000014672
M: Sh-IRF1#1	Sigma-Aldrich	Cat# TRCN0000077440
M: Sh-IRF1#2	Sigma-Aldrich	Cat# TRCN0000077441

Abbreviations: H, human; m, mouse

Supplementary References

1. Yu H. et al. Major Vault Protein Promotes Hepatocellular Carcinoma Through Targeting Interferon Regulatory Factor 2 and Decreasing p53 Activity. *Hepatology* 2019;72: 518-534.

2. Zhou L. et al. Hepatitis B virus rigs the cellular metabolome to avoid innate immune recognition. *Nature Communications* 2021;12,98.

3. Wang Q. et al. O-GlcNAc Transferase Promotes Influenza A Virus-Induced Cytokine Storm by Targeting Interferon Regulatory Factor-5. *Science Advances* 2020; 6:16, eaaz7086.

Source Data

Fig 2g:

(i)-(iii): Because of similar molecular weight, the same samples were run on different gels and the membranes were incubated with the indicated antibodies.

Fig 2g

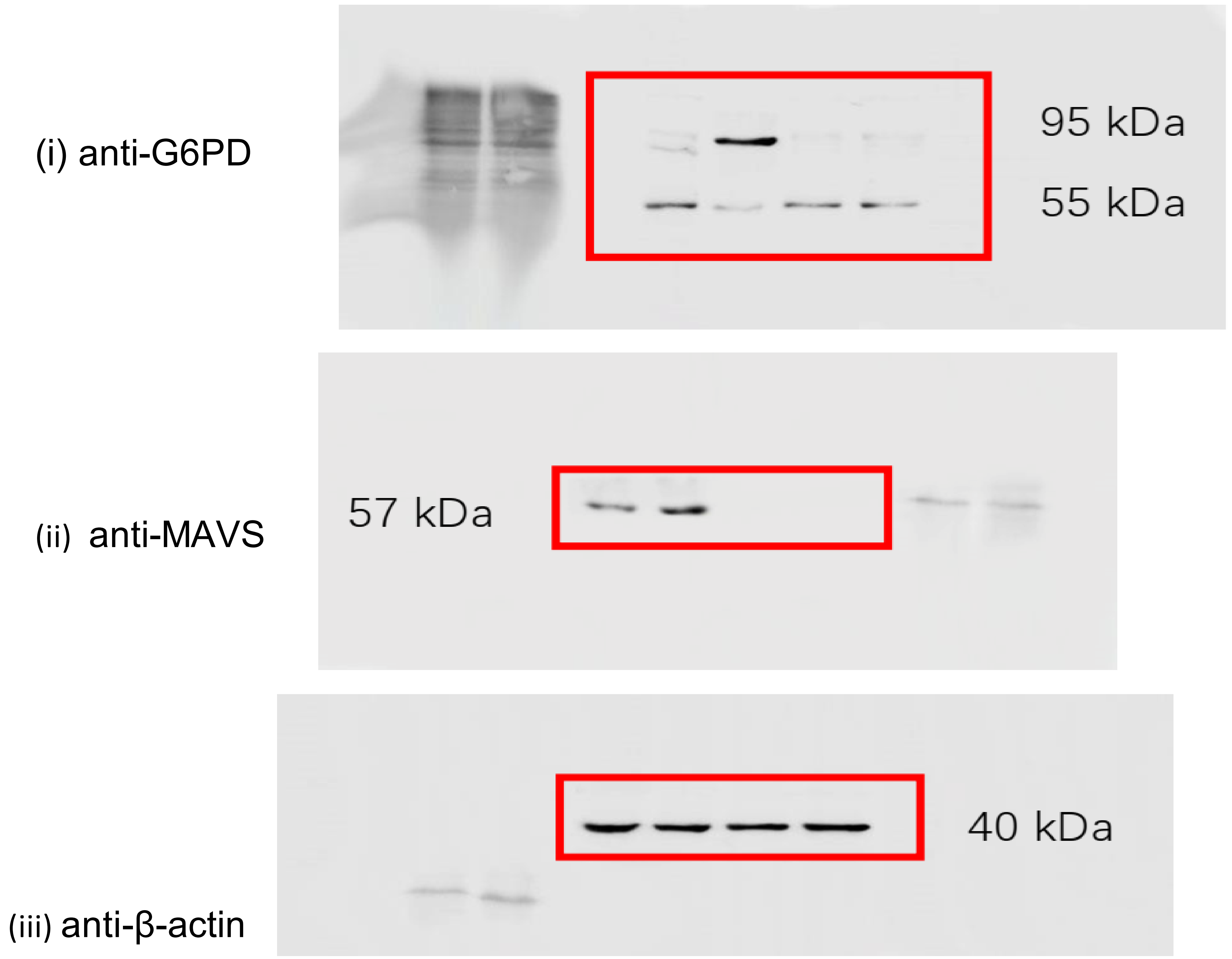

Fig 6a
(i) ,(ii):): Because of similar molecular weight, the same samples were separated on two gels, and the membranes were cut and incubated with the indicated antibodies.
(iii),(iv): Another set of the same samples were separated on one gel, and the membrane was cut and incubated with the indicated antibodies.

Fig 6b
(i) ,(ii): Because of similar molecular weight, the same samples were separated on two gels, and the membranes was cut and incubated with the indicated antibodies.
(iii) (iv),(v): Because of similar molecular weight, another set of the same samples were separated on two gels, and the membranes were cut and incubated with the indicated antibodies.

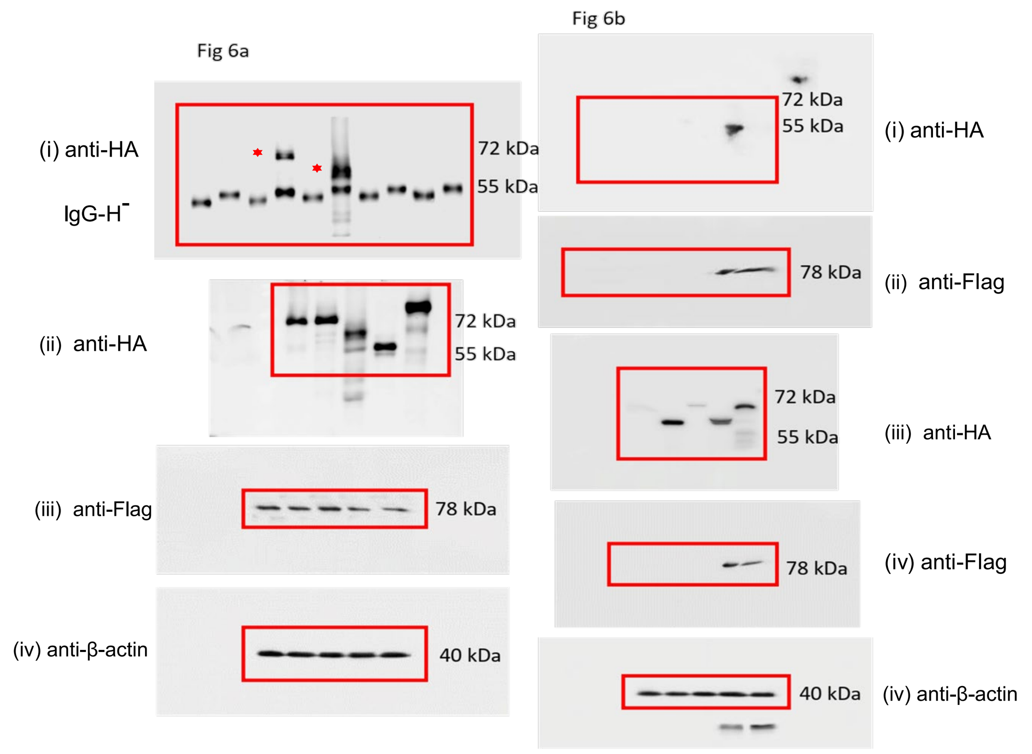

Fig 6c
(i)-(v):Because of similar molecular weight, the same samples were separated on two gels, and the membranes were cut and incubated with the indicated antibodies.
(vi)-(xi): Because of similar molecular weight, another set of the same samples were separated on three gels, and the membranes were cut and incubated with the indicated antibodies.

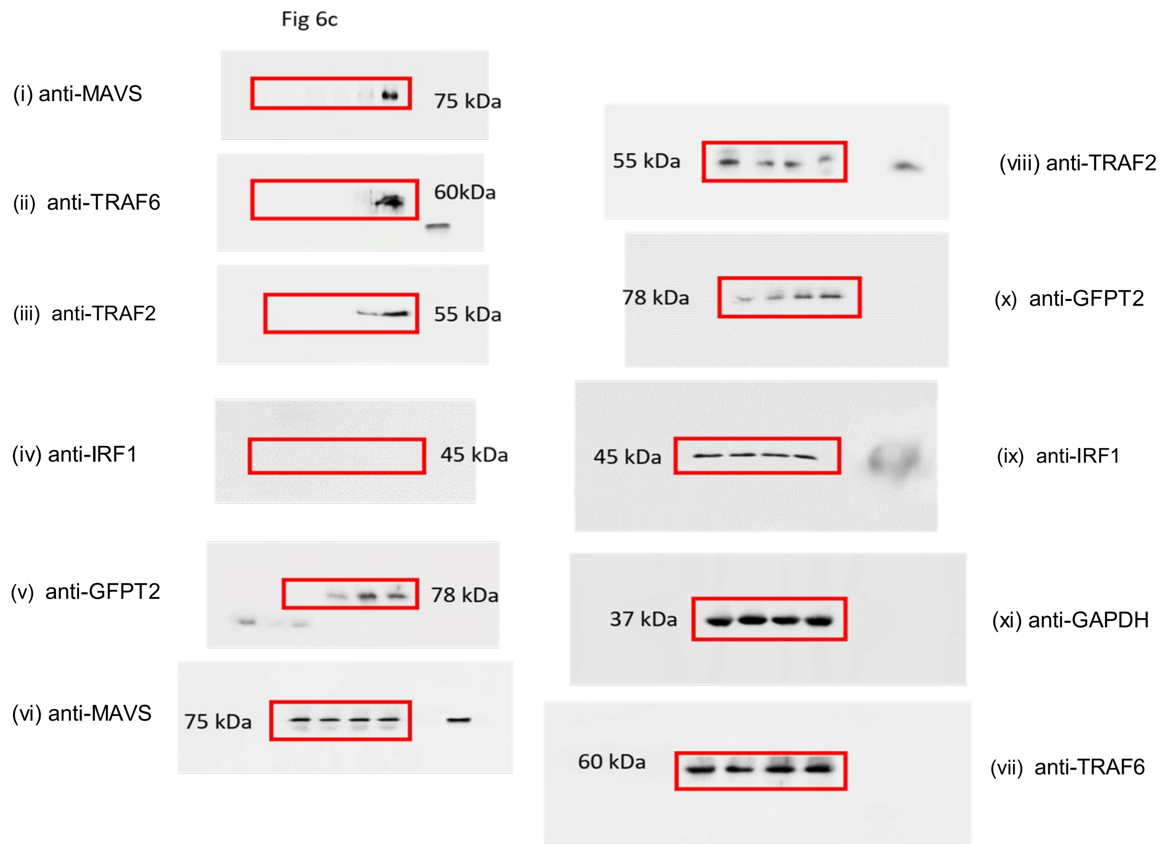

Fig 6d
(i)-(iv): The same samples were separated on two gels, and the membranes were cut and incubated with the indicated antibodies.
(v)-(ix): Another set of the same samples were separated on two gels, and the membranes were cut and incubated with the indicated antibodies.
Fig 6e
(i)-(iv):): The same samples were separated on two gels, and the membranes were cut and incubated with the indicated antibodies.
(v)-(ix): Another set of the same samples were separated on two gels, and the membranes were cut and incubated with the indicated antibodies.

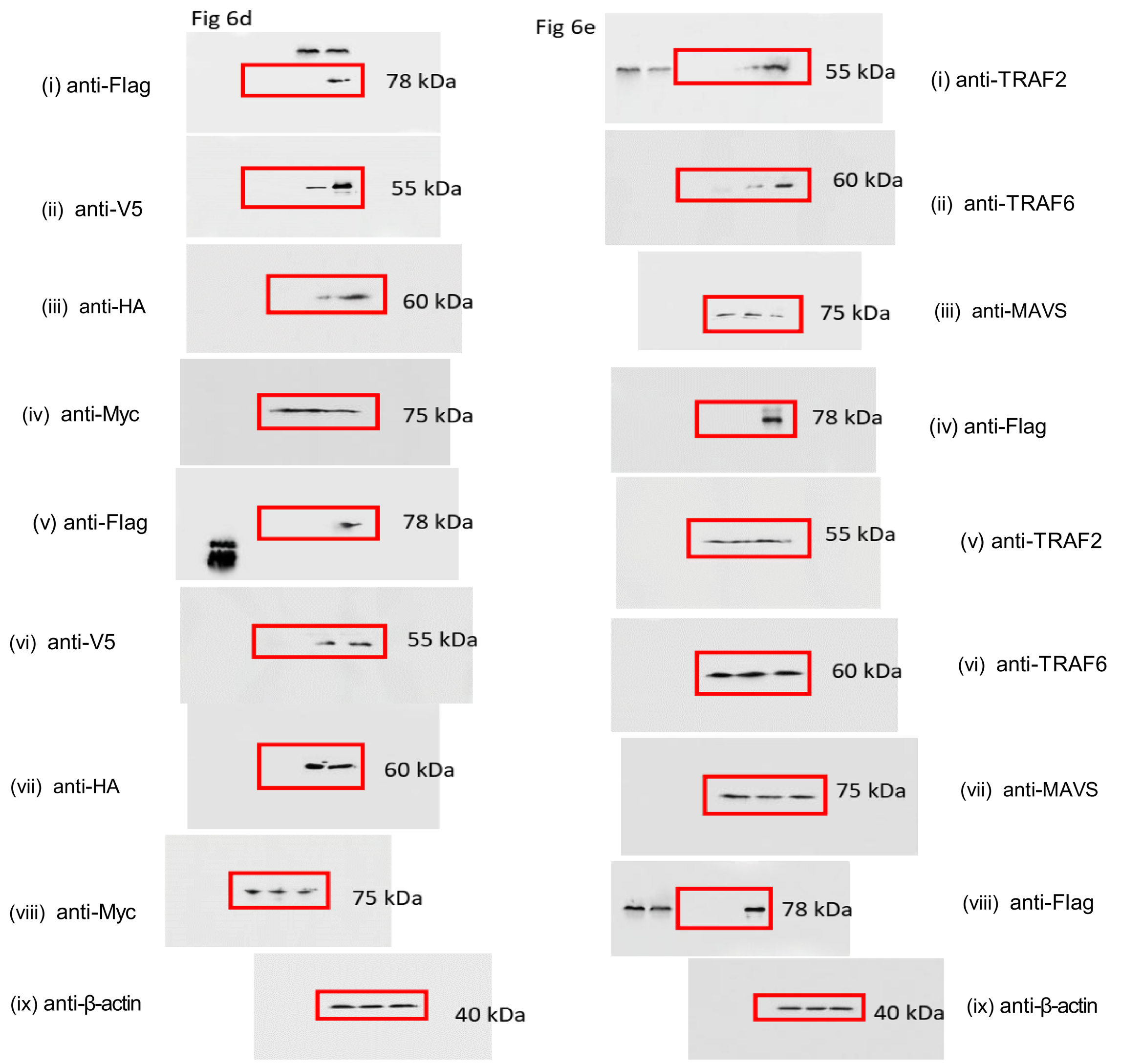

Fig 6f
(i)-(iii): The same samples were separated on three gels, the membranes were cut and incubated with the indicated antibodies.
(iv)-(vi): Another set of the same samples were separated on two gels, and the membranes were cut and incubated with the indicated antibodies.
Fig 6g
(i)-(iii): The same samples were separated on three gels, the membranes were cut and incubated with the indicated antibodies.
(iv)-(vi): Another set of the same samples were separated on two gels, and the membranes were cut and incubated with the indicated antibodies.

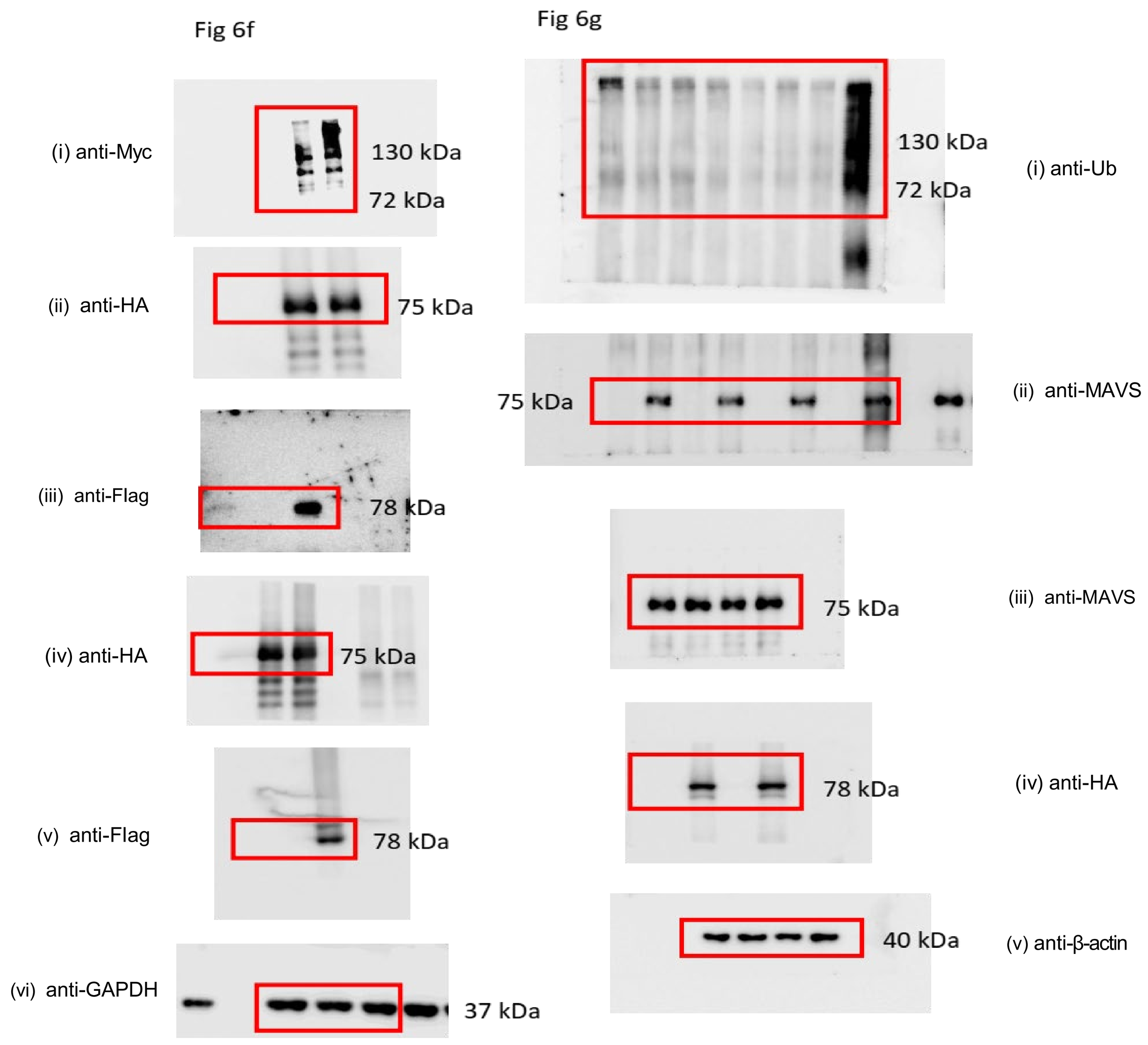

Fig 6h
(i) ,(ii): The same samples were separated on three gels, the membranes were cut and incubated with the indicated antibodies.
(iii)-(v): Another set of the same samples were separated on two gels, and the membranes were cut and incubated with the indicated antibodies.
Fig 6i
(i) ,(ii): The same samples were separated on three gels, the membranes were cut and incubated with the indicated antibodies.
(iii)-(v): Another set of the same samples were separated on two gels, and the membranes were cut and incubated with the indicated antibodies.

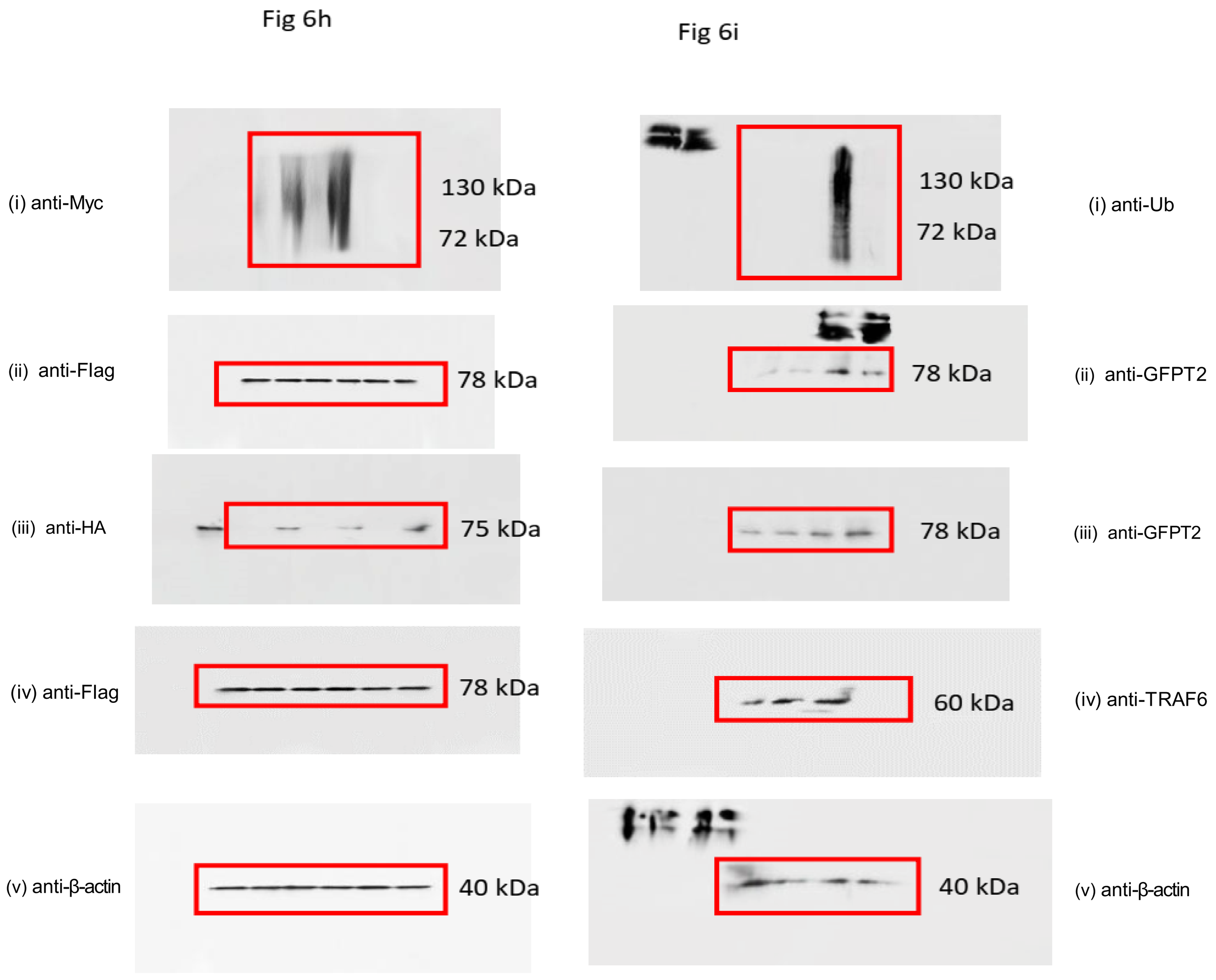

Fig 7a

Group ①: The samples were separated on two gels, and the membranes were cut and incubated with the indicated antibodies. (i) and (ii) are one set of same samples. (iii),(iv) and (v) are another set of same samples.

Group ②: The samples were separated on two gels, and the membranes were cut and incubated with the indicated antibodies. (i) and (ii) are one set of same samples. (iii),(iv) and (v) are another set of same samples.

Group③ : The samples were separated on two gels, and the membranes were cut and incubated with the indicated antibodies. (i) and (ii) are one set of same samples. (iii),(iv) and (v) are another set of same samples.

Group④ : The samples were separated on two gels, and the membranes were cut and incubated with the indicated antibodies. (i) and (ii) are one set of same samples. (iii),(iv) and (v) are another set of same samples.

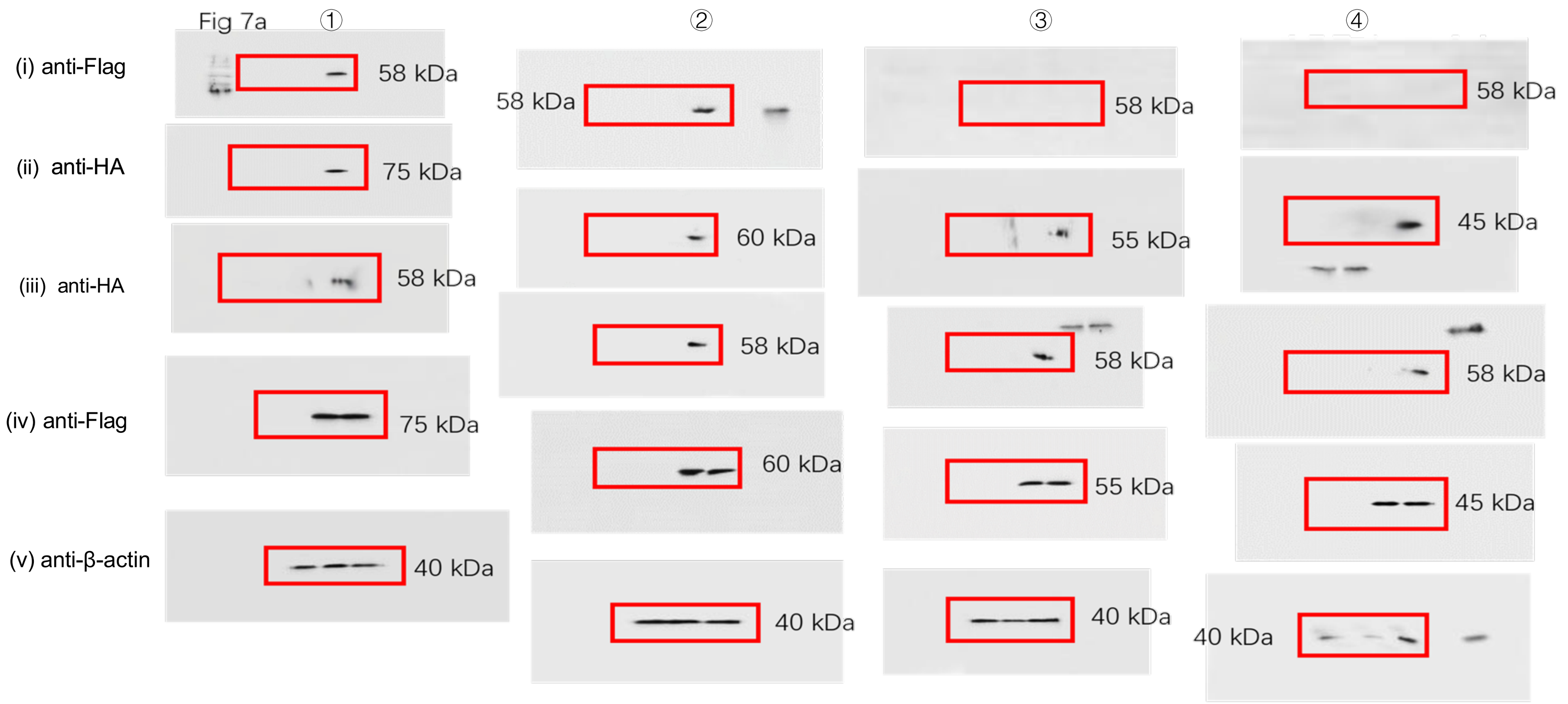

Fig 7b

(i)-(v): The same samples were separated on two gels, and the membranes were cut and incubated with the indicated antibodies.

(vi)-(xi): Another set of the same samples were separated on three gels, and the membrane were cut and incubated with the indicated antibodies.

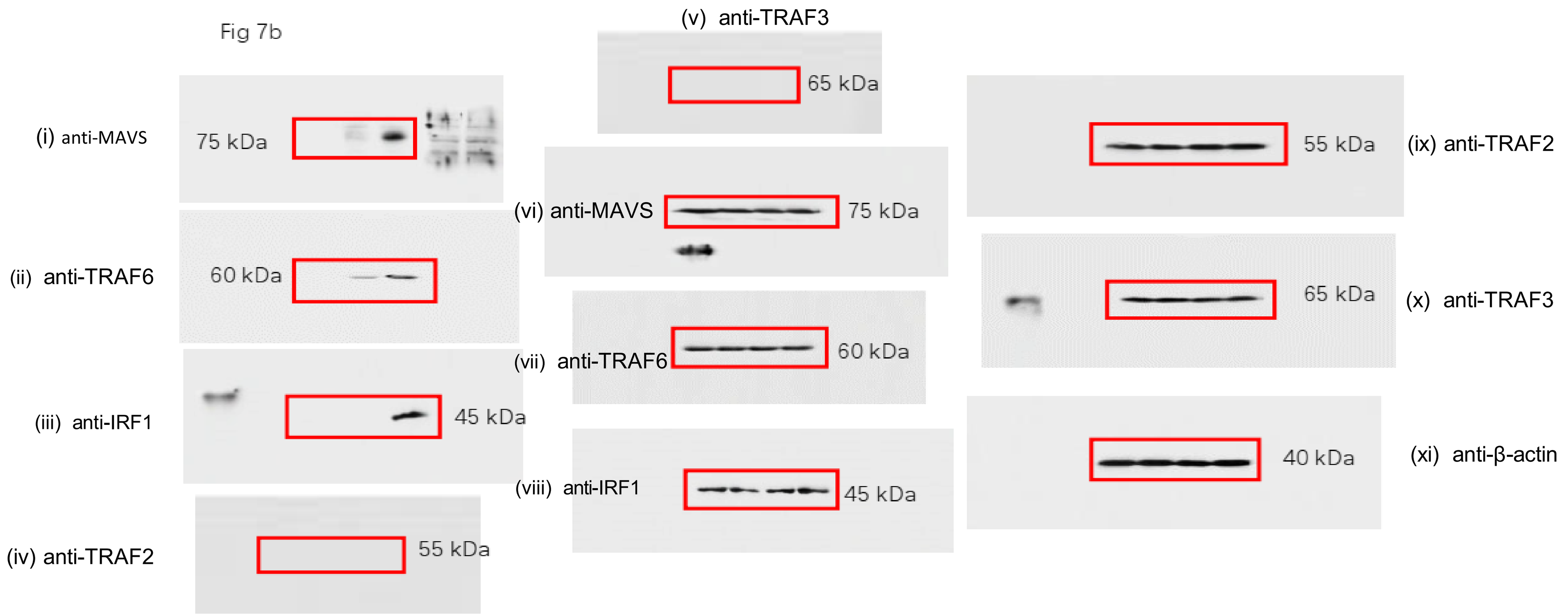

Fig 7c

(i)-(iv): The same samples were separated on three gels, and the membrane were cut and incubated with the indicated antibodies.

(v)-(ix): Another set of the same samples were separated on four gels, and the membranes were cut and incubated with the indicated antibodies.

Fig 7d

(i)-(iv): The same samples were separated on three gels, and the membrane were cut and incubated with the indicated antibodies.

v)-(ix): Another set of the same samples were separated on three gels, and the membranes were cut and incubated with the indicated antibodies.

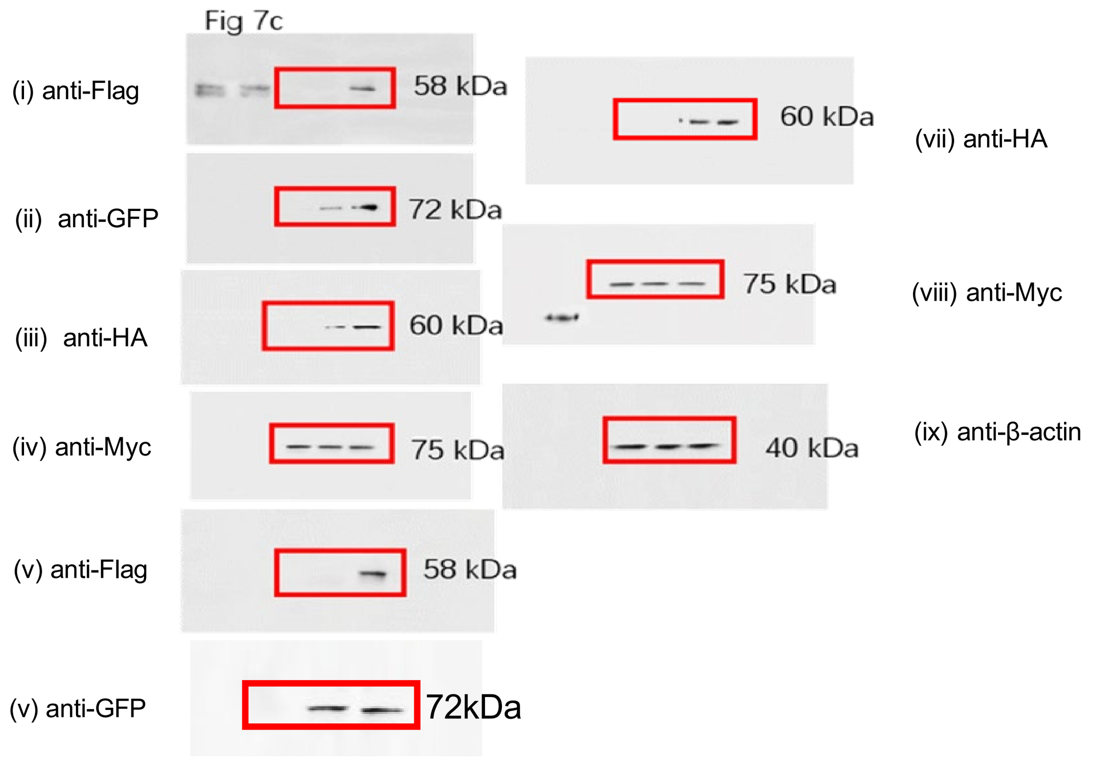

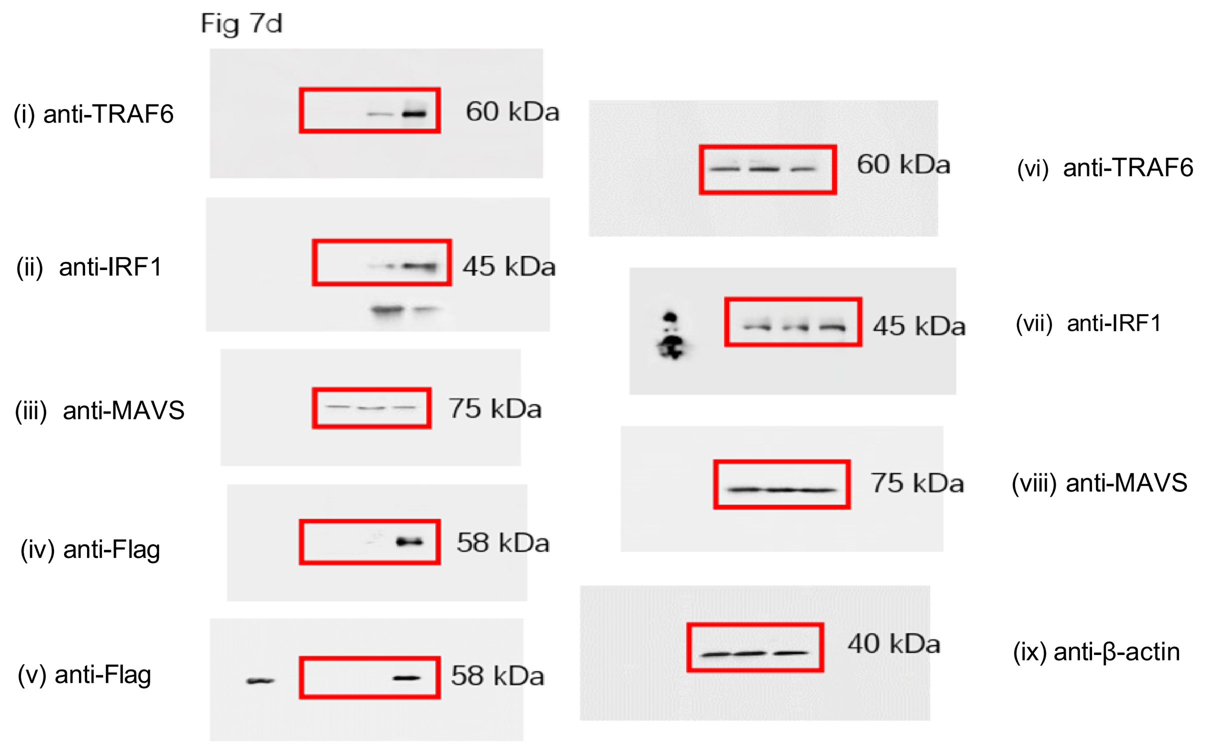

Fig 7e

(i)-(ii): The same samples were separated on two gels, and the membranes were cut and incubated with the indicated antibodies.

(iii)-(v): Another set of the same samples were separated on three gels, and the membranes were cut and incubated with the indicated antibodies.

Fig 7f

(i)-(ii): The same samples were separated on two gels, and the membranes were cut and incubated with the indicated antibodies.

(iii)-(vi):): Another set of the same samples were separated on three gels, and the membranes were cut and incubated with the indicated antibodies

Fig 7g

(i)-(iv): The same samples were separated on two gels, and the membranes were cut and incubated with the indicated antibodies.

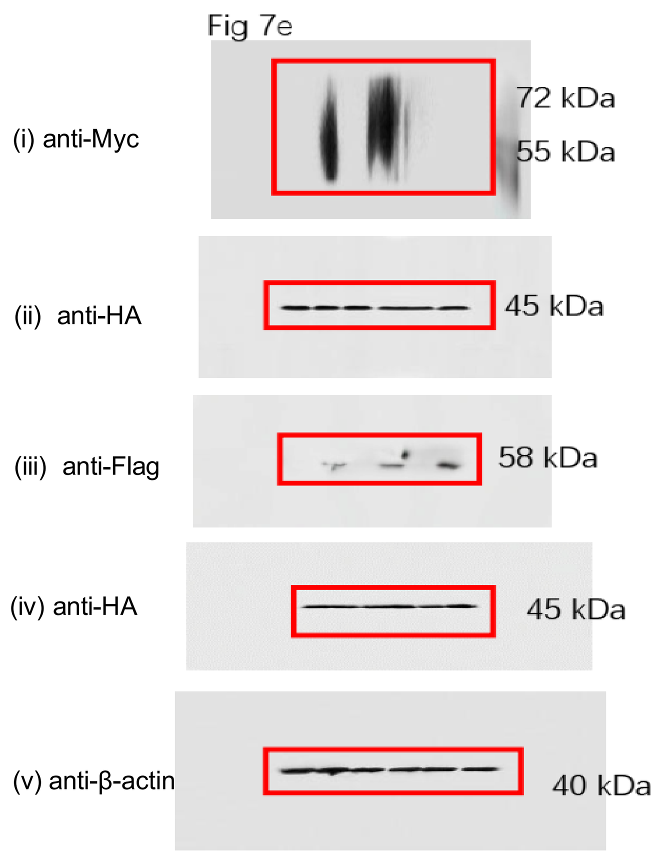

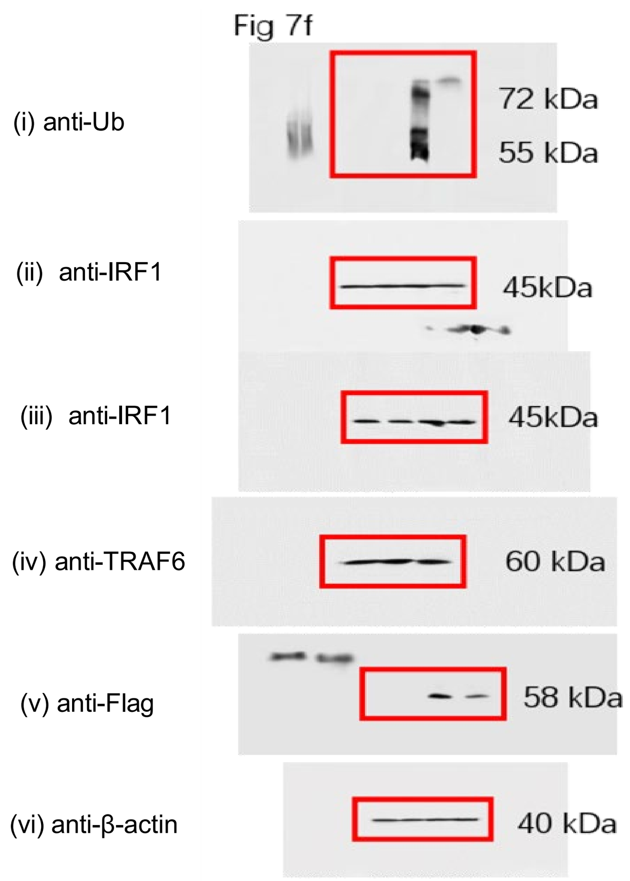

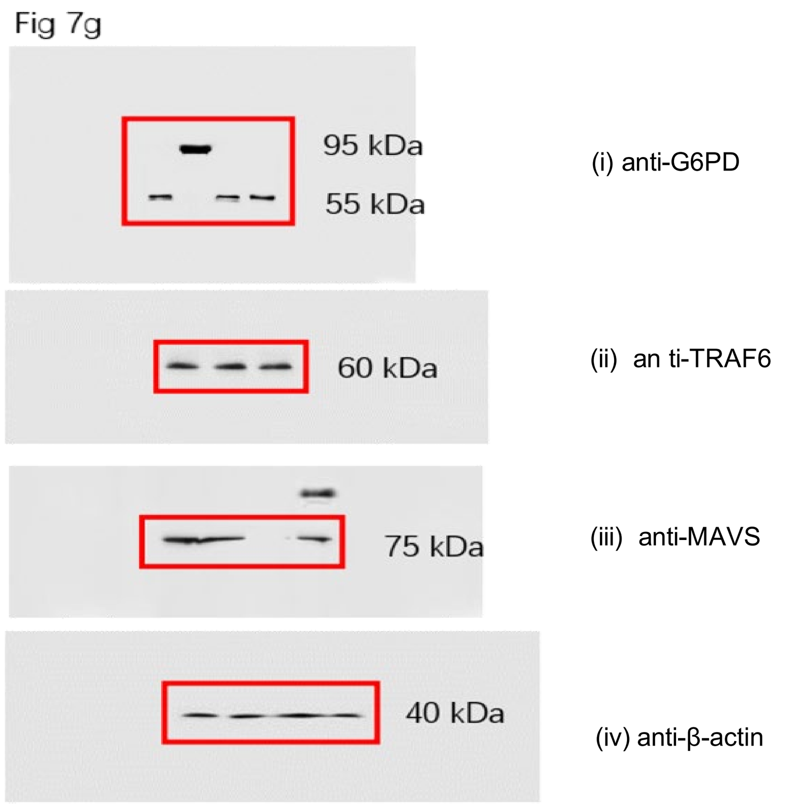

Fig 8a

(i)-(vii): The same samples were separated on three gels, and the membranes were cut and incubated with the indicated antibodies.

(viii)-(xv): Another set of the same samples were separated on four gel, and the membranes were cut and incubated with he indicated antibodies.

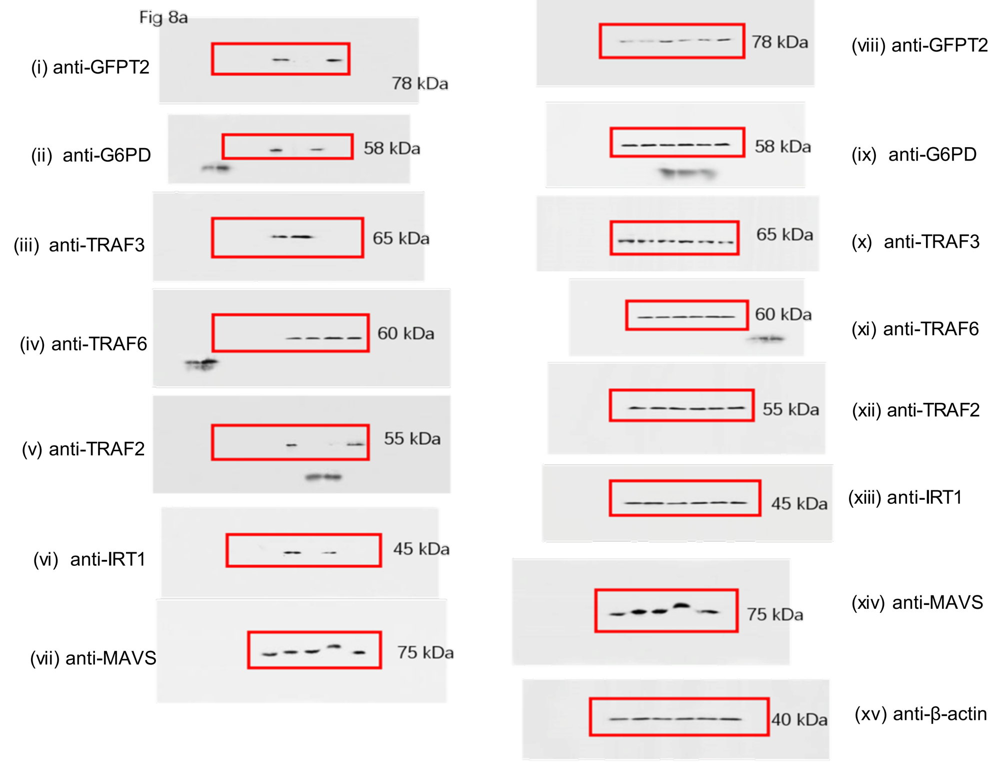

Fig 8b

(i): The same samples were separated on one gel, and the membranes were cut and incubated with the indicated antibodies.

(ii)-(iii): The same samples were separated one gel, and the membrane was cut and incubated with the indicated antibodies.

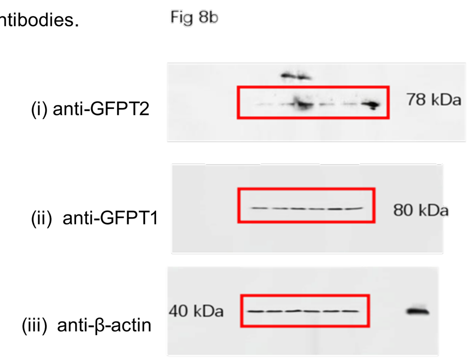

Fig 8c

(i)-(ii): The same samples were separated on two gel, and the membranes were cut and incubated with the indicated antibodies.

(iii)-(v): The same samples were separated two gel, and the membrane were cut and incubated with the indicated antibodies.

Fig 8e

(i)-(ii): The same samples were separated one gel, and the membrane was cut and incubated with the indicated antibodies.

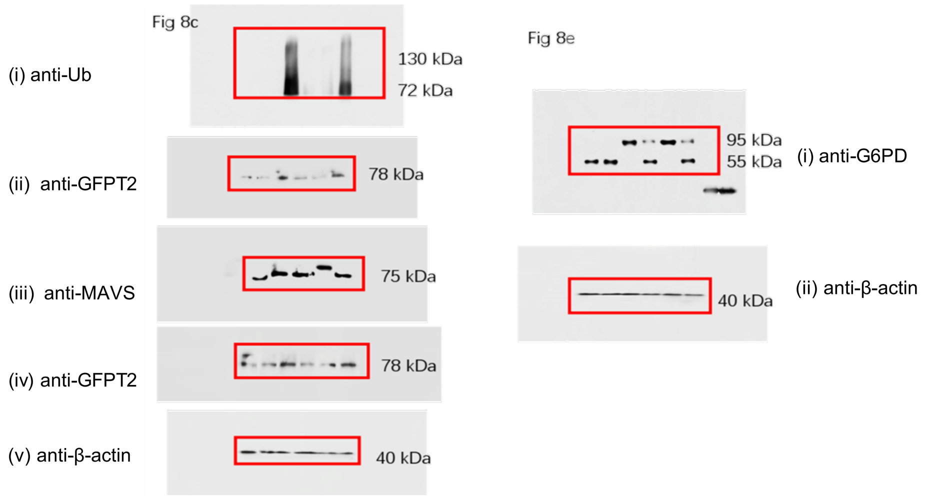

Fig 8f group1-4

Group 1 Mitochondria

(i)-(xi) :The same samples were separated five gel, and the membrane was cut and incubated with the indicated antibodies.

Group 2 MAM

(i)-(xi) :The same samples were separated five gel, and the membrane was cut and incubated with the indicated antibodies.

Group 3 Peroxisome

(i)-(xi) :The same samples were separated five gel, and the membrane was cut and incubated with the indicated antibodies.

Group 4 Cytosol

(i)-(xi) :The same samples were separated five gel, and the membrane was cut and incubated with the indicated antibodies.

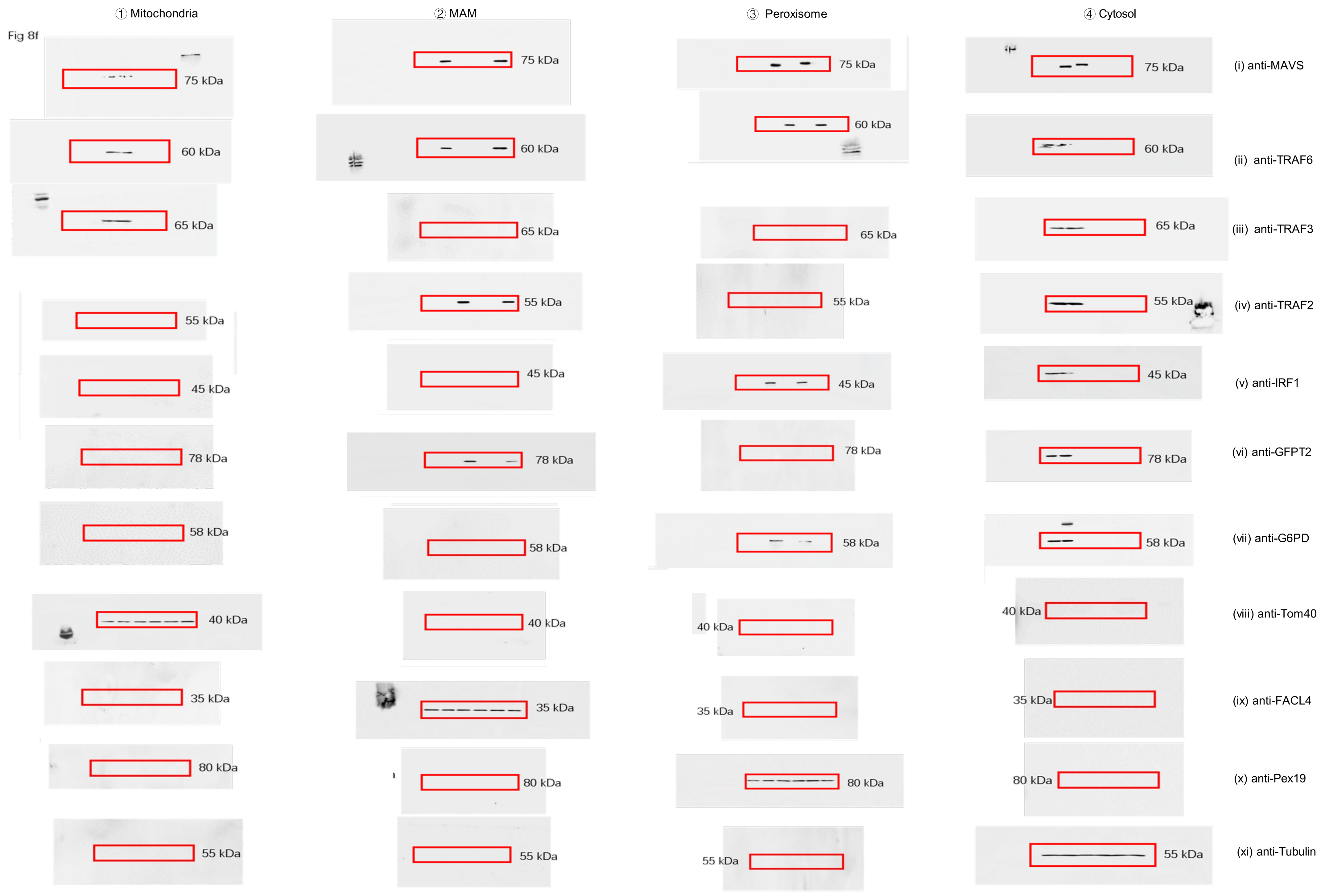

Fig s1a-b

(i)-(ii) :The samples were separated one gel, and the membrane was cut and incubated with the indicated antibodies.

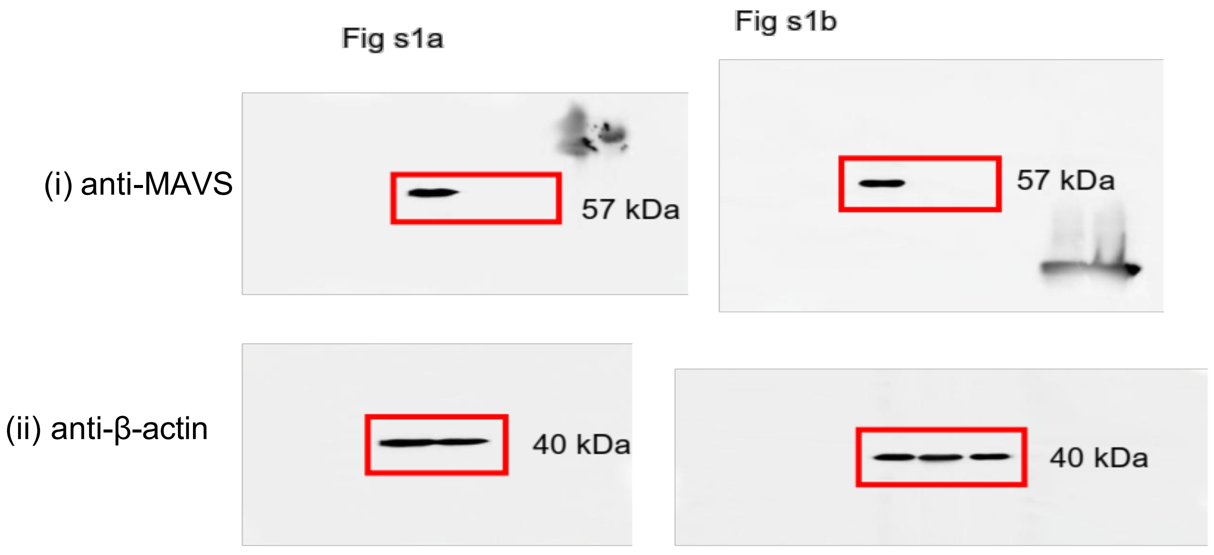

Fig s3n

(i)-(iii) :The samples were separated two gel, and the membrane was cut and incubated with the indicated antibodies.

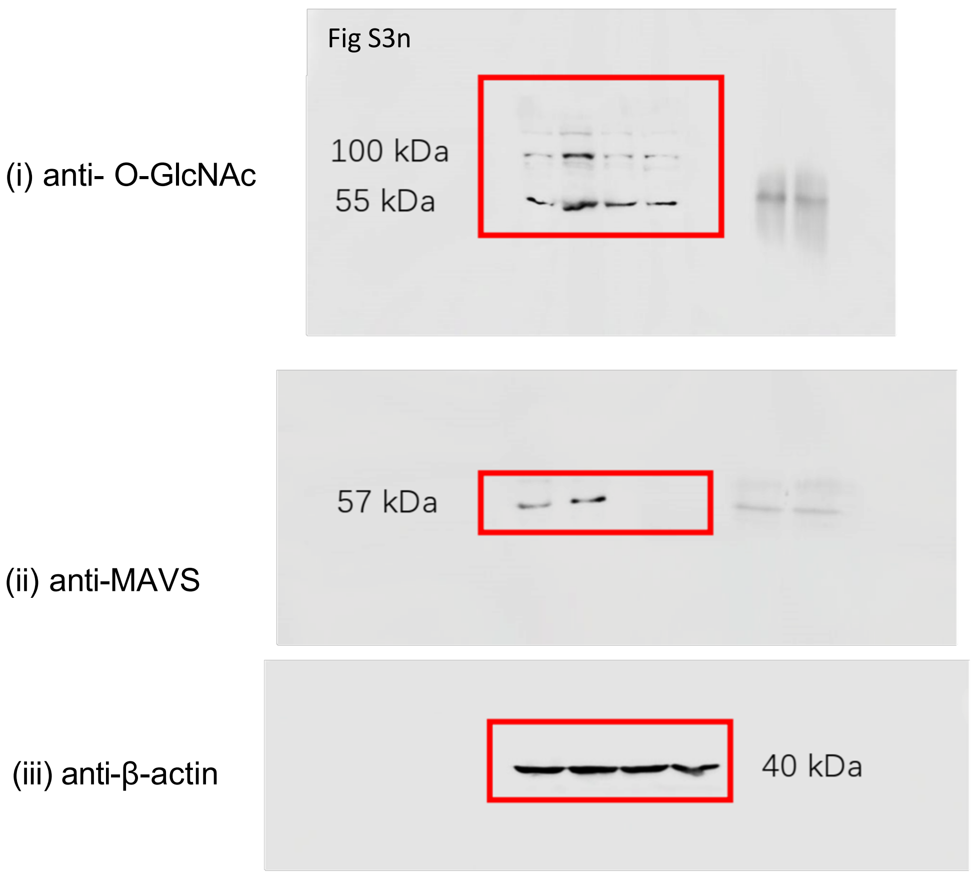

Figs4c group1-4

Group 1 Mitochondria

(i)-(v) : The same samples were separated two gel, and the membranes were cut and incubated with the indicated antibodies.

Group 2 Peroxisome

(i)-(v) : The same samples were separated two gel, and the membranes were cut and incubated with the indicated antibodies.

Group 3 MAM

(i)-(v) : The same samples were separated two gel, and the membranes were cut and incubated with the indicated antibodies.

Group 4 Cytosol

(i)-(v) : The same samples were separated two gel, and the membranes were cut and incubated with the indicated antibodies.

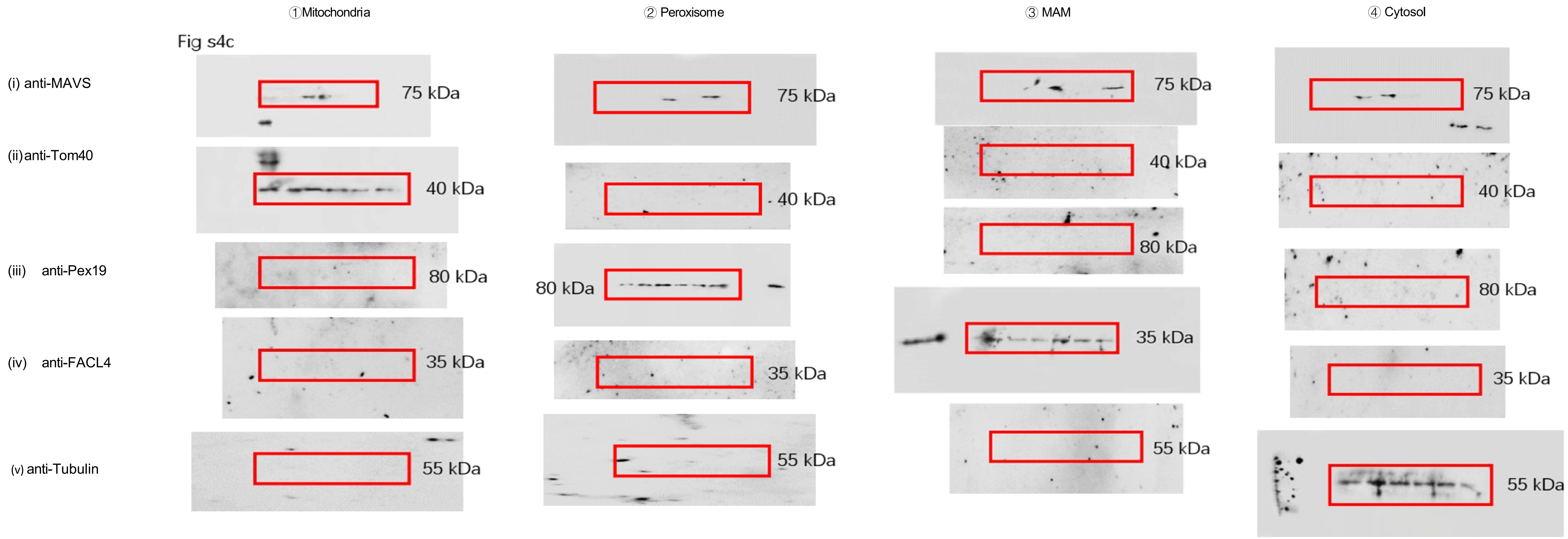

Fig s7C

(i)-(V) :The samples were separated two gels, and the membranes were cut and incubated with the indicated antibodies.

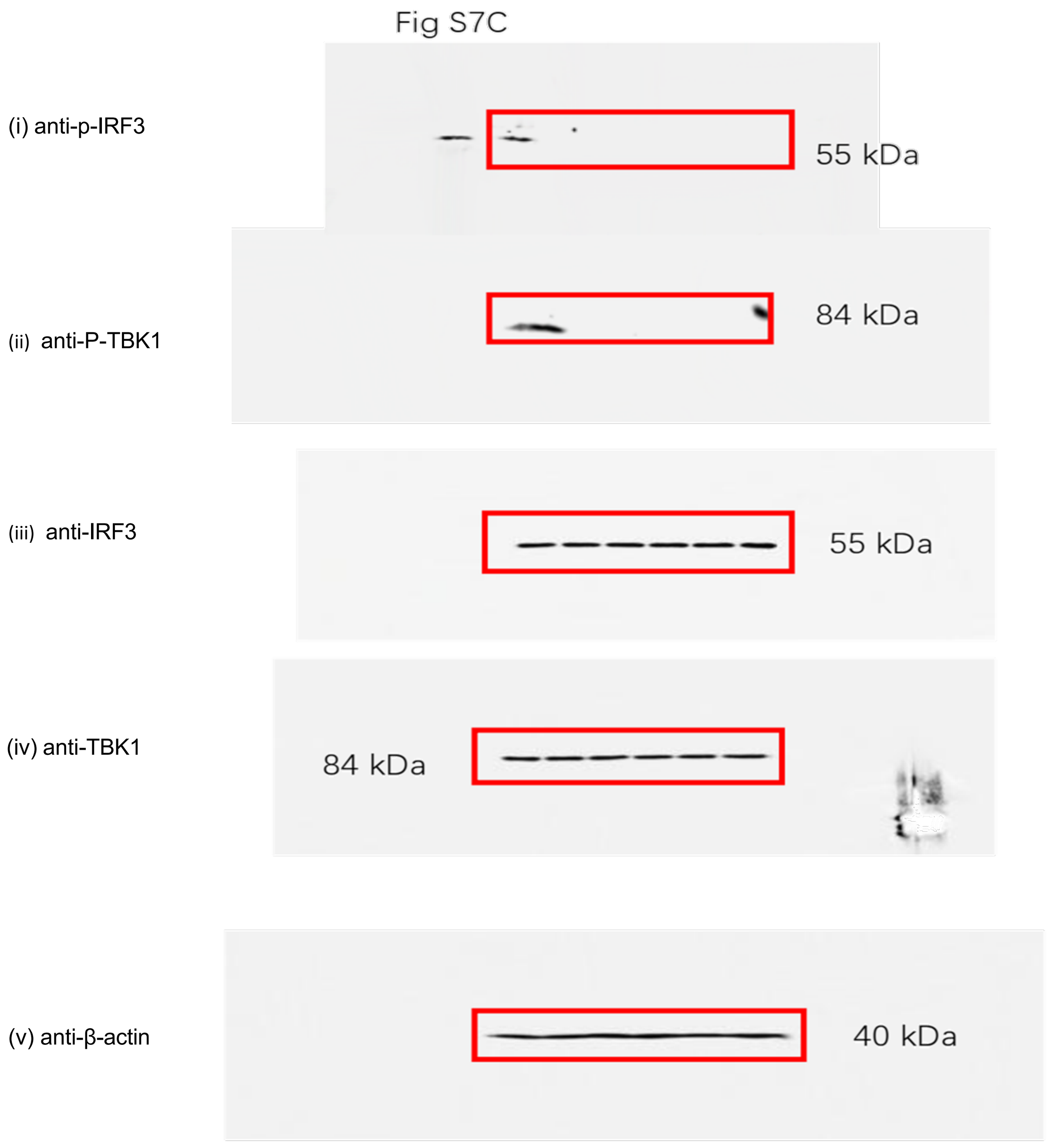

Fig s9a-b

(i)-(ii) :The samples were separated one gel, and the membrane was cut and incubated with the indicated antibodies.

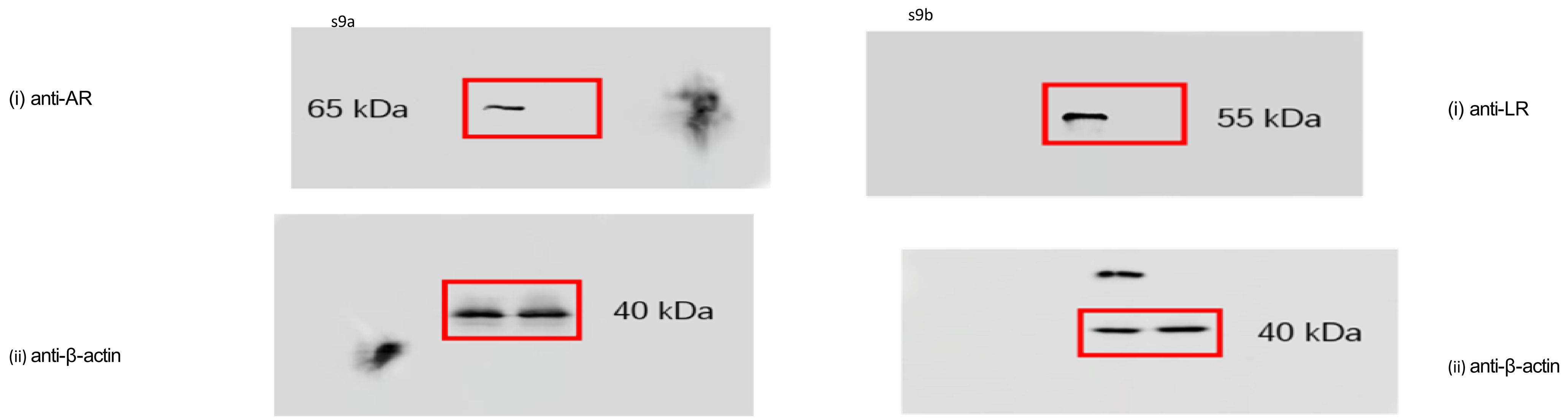

Fig s11a

(i):The samples were separated one gel, and the membrane was incubated with the indicated antibodies.

(ii)-(iii) :The same samples were separated two gels, and the membranes were cut and incubated with the indicated antibodies.

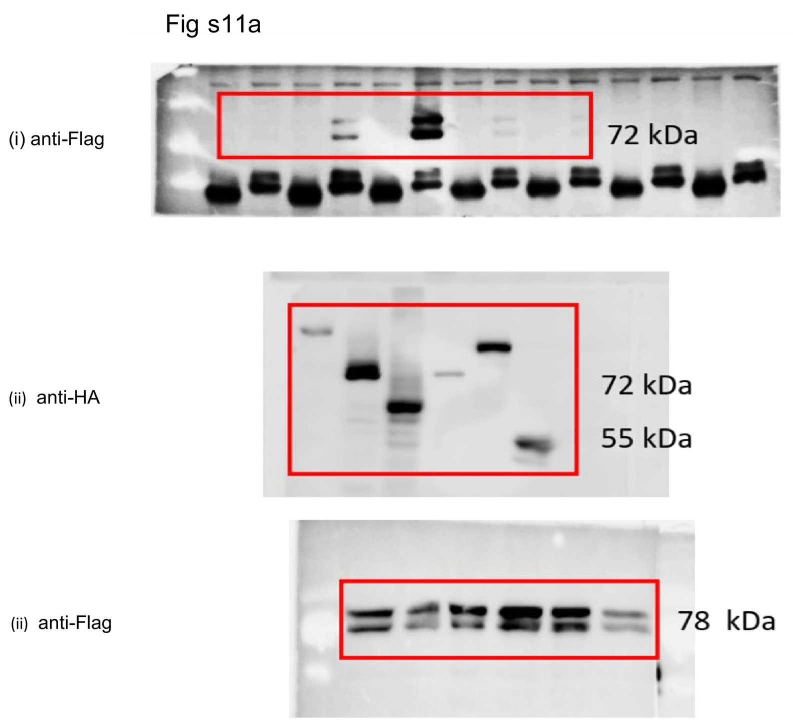

Fig s11b

(i) ,(ii): The same samples were separated two gels, and the membranes were cut and incubated with the indicated antibodies.

(iii)(iv),(v): Another set of the same samples were separated two gels, and the membranes were cut and incubated with the indicated antibodies

Fig s11c

(i) ,(ii): The same samples were separated two gels, and the membranes were cut and incubated with the indicated antibodies.

(iii)(iv),(v): Another set of the same samples were separated two gels, and the membranes were cut and incubated with the indicated antibodies

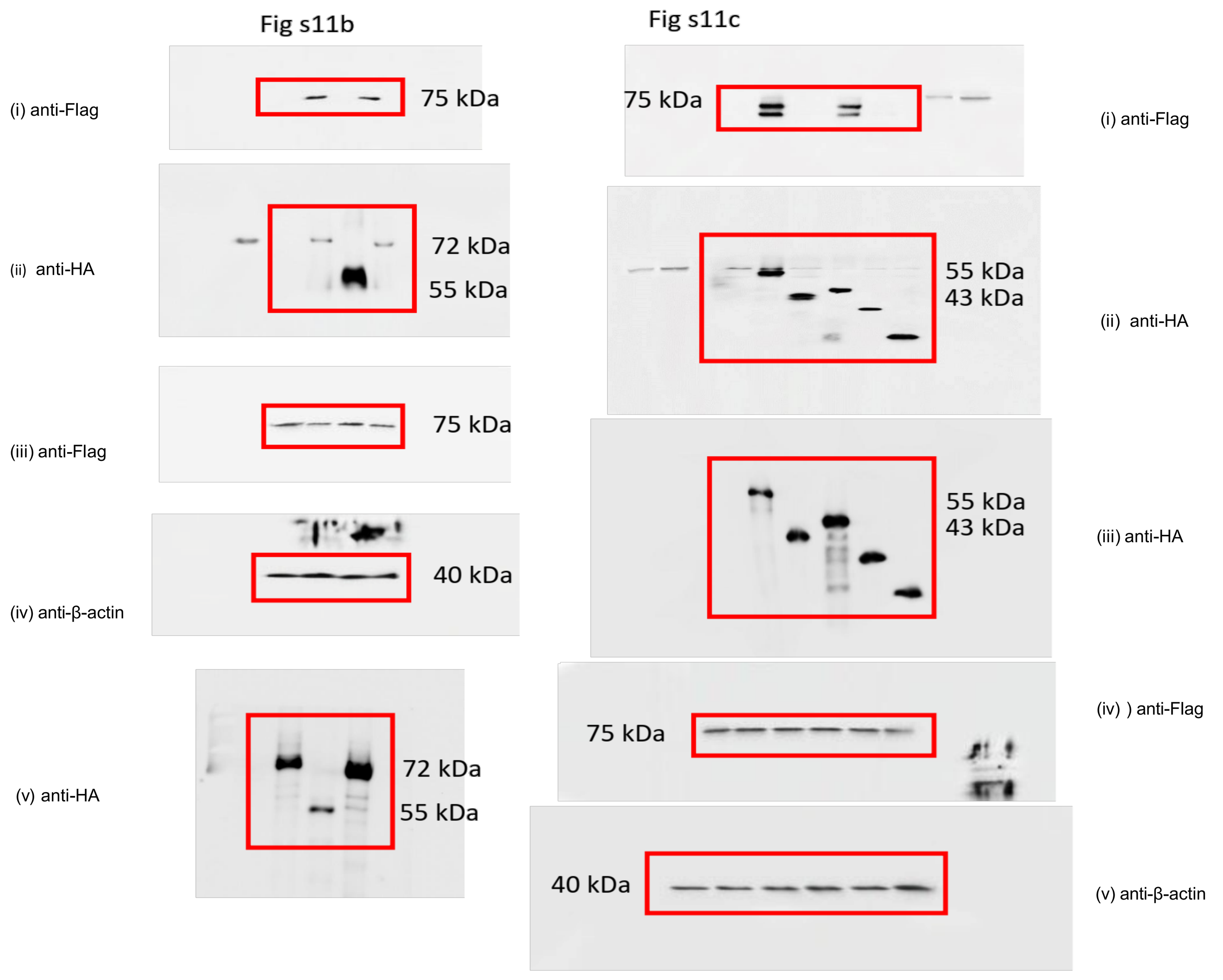

Fig s11d

(i) ,(ii): The same samples were separated on one gel, and the membrane was cut and incubated with the indicated antibodies.

(iii)(iv),(v): Another set of the same samples were separated on two gels, and the membranes were cut and incubated with the indicated antibodies.

Fig s11e

(i) ,(ii): The same samples were separated on one gel, and the membrane was cut and incubated with the indicated antibodies.

(iii)(iv),(v): Another set of the same samples were separated on two gels, and the membranes were cut and incubated with the indicated antibodies.

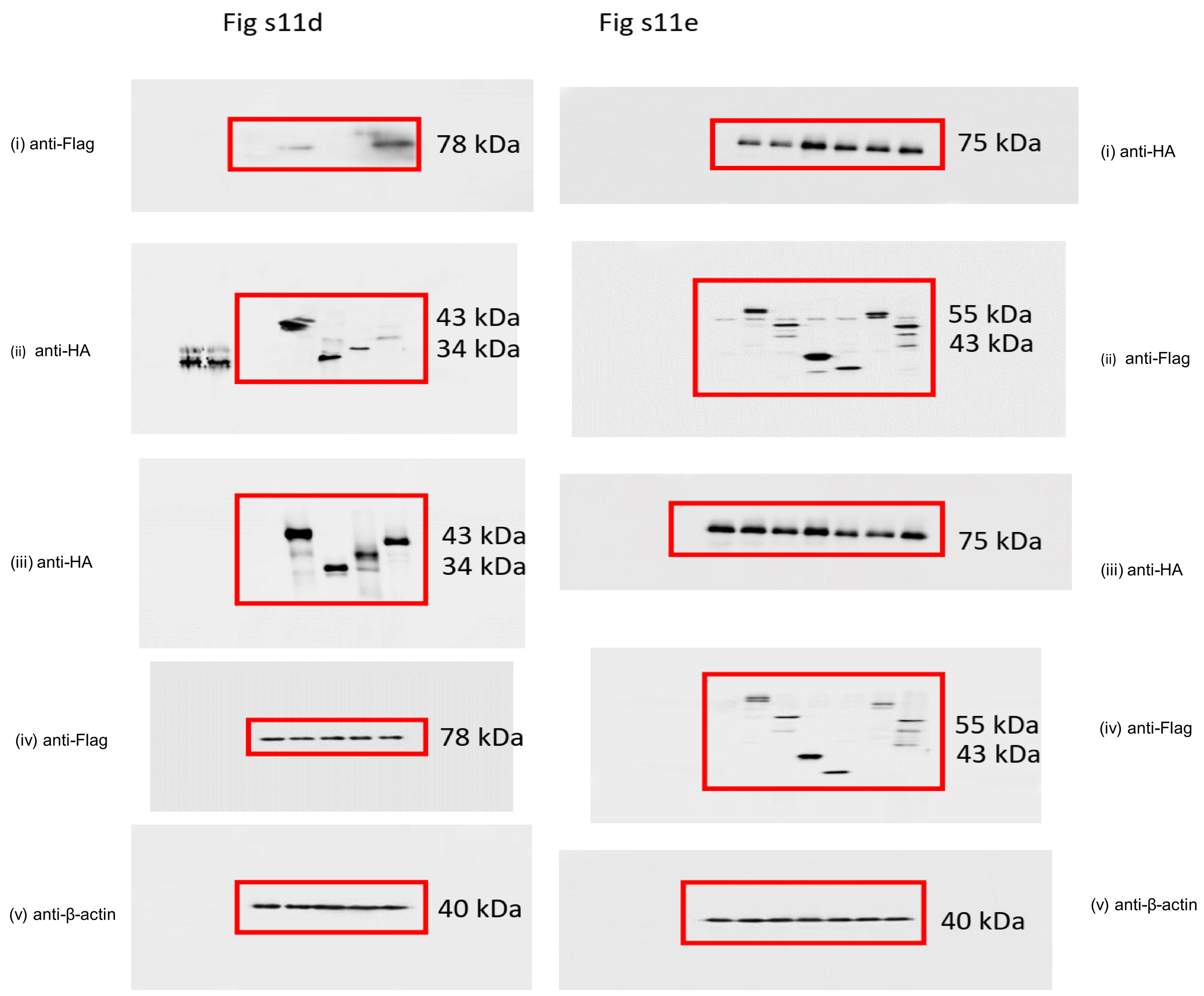

Fig s11f

(i) ,(ii): The same samples were separated on two gels, and the membranes were cut and incubated with the indicated antibodies.

(iii)(iv),(v): Another set of the same samples were separated on two gels, and the membranes were cut and incubated with the indicated antibodies.

Fig s11g

(i) ,(ii): The same samples were separated on two gels, and the membranes were cut and incubated with the indicated antibodies.

(iii)(iv),(v): Another set of the same samples were separated on two gels, and the membranes were cut and incubated with the indicated antibodies.

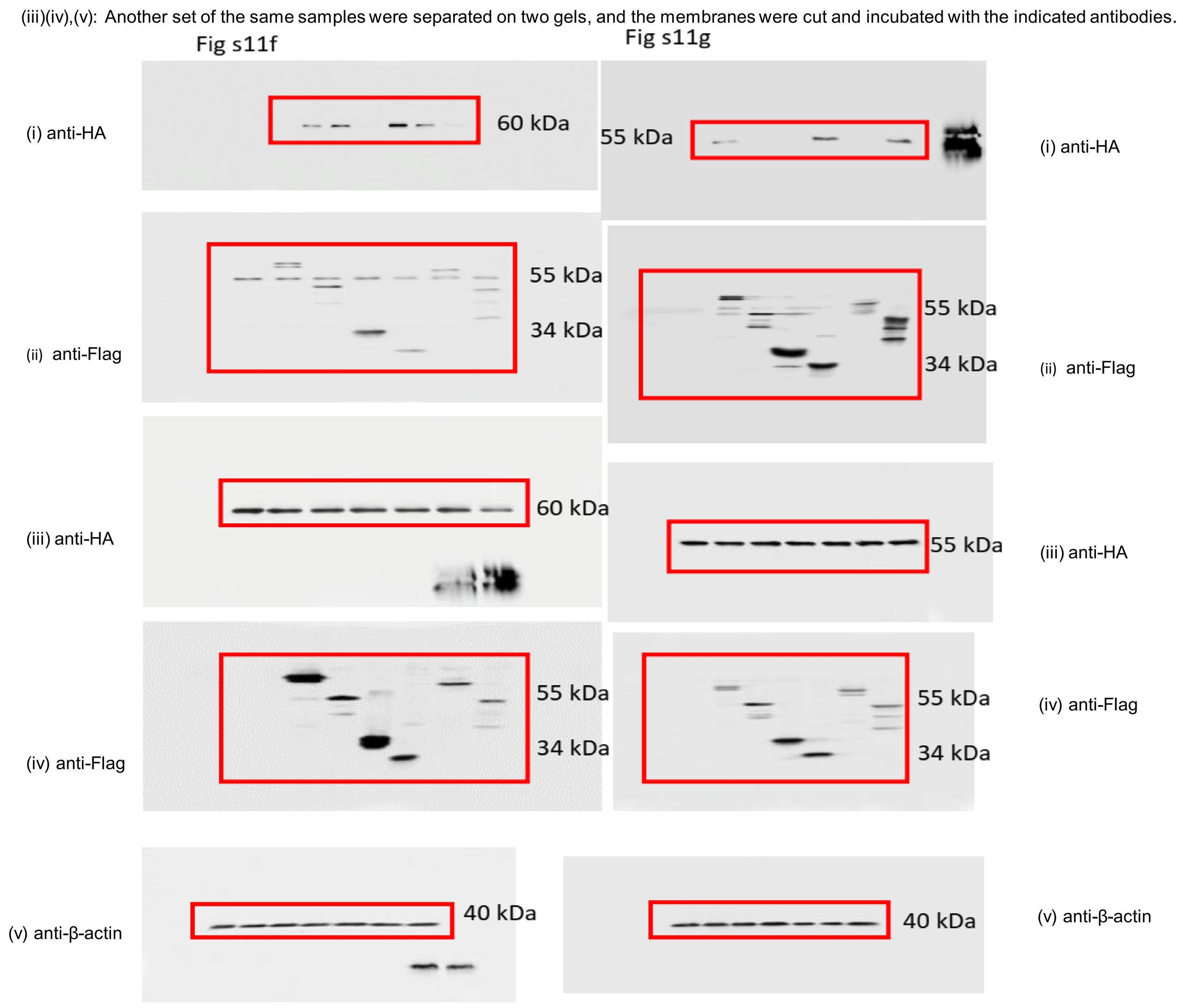

Fig s12a

(i)-(vii): The same samples were separated on five gels, and the membranes were cut and incubated with the indicated antibodies.

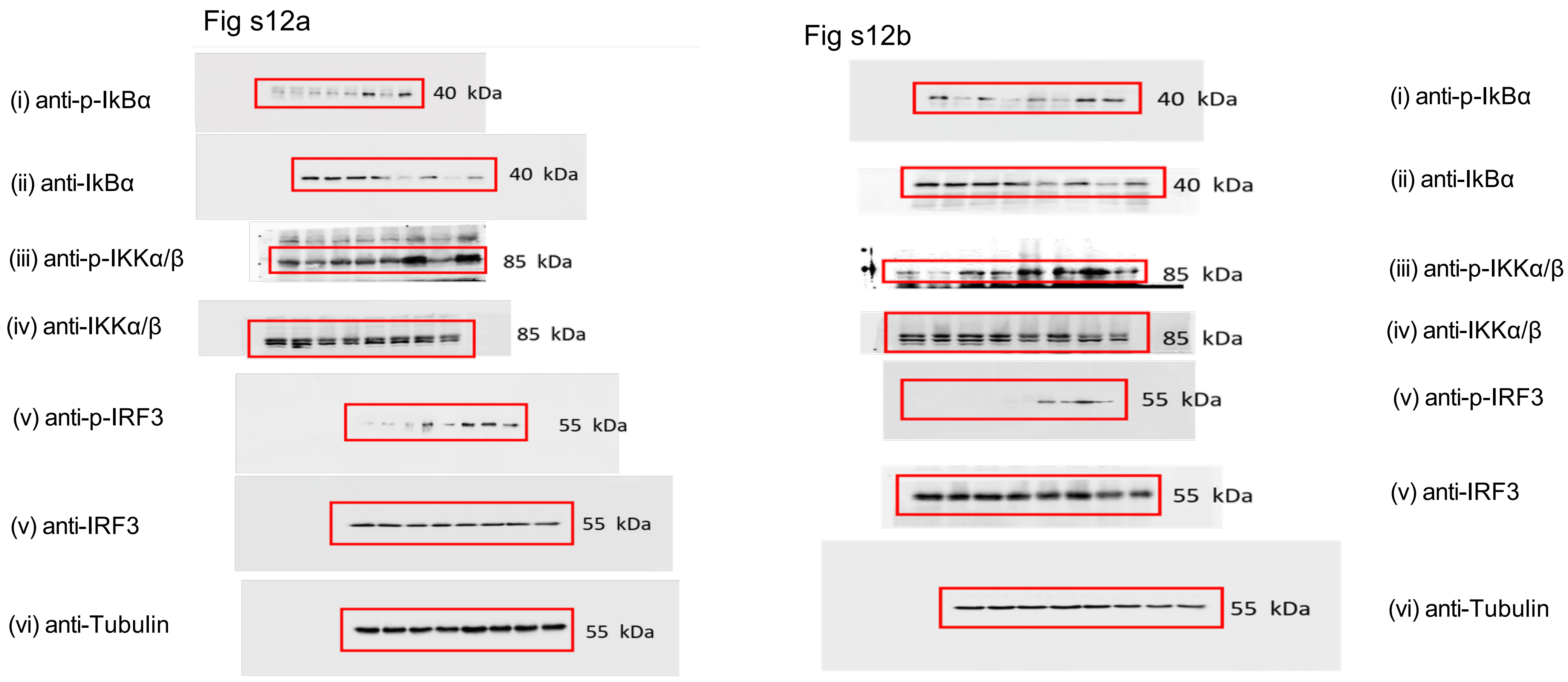

Fig s13a

(i) ,(ii): The same samples were separated on two gels, and the membranes were cut and incubated with the indicated antibodies.

(iii)(iv),(v): Another set of the same samples were separated on two gels, and the membranes were cut and incubated with the indicated antibodies.

Fig s13b

(i) ,(ii): The same samples were separated on two gels, and the membranes were cut and incubated with the indicated antibodies.

Fig s13c

(i) ,(ii): The same samples were separated on two gels, and the membranes were cut and incubated with the indicated antibodies.

(iii)(iv),(v): Another set of the same samples were separated on two gels, and the membranes were cut and incubated with the indicated antibodies.

Fig s13d

(i) ,(ii): The same samples were separated on two gels, and the membranes were cut and incubated with the indicated antibodies.

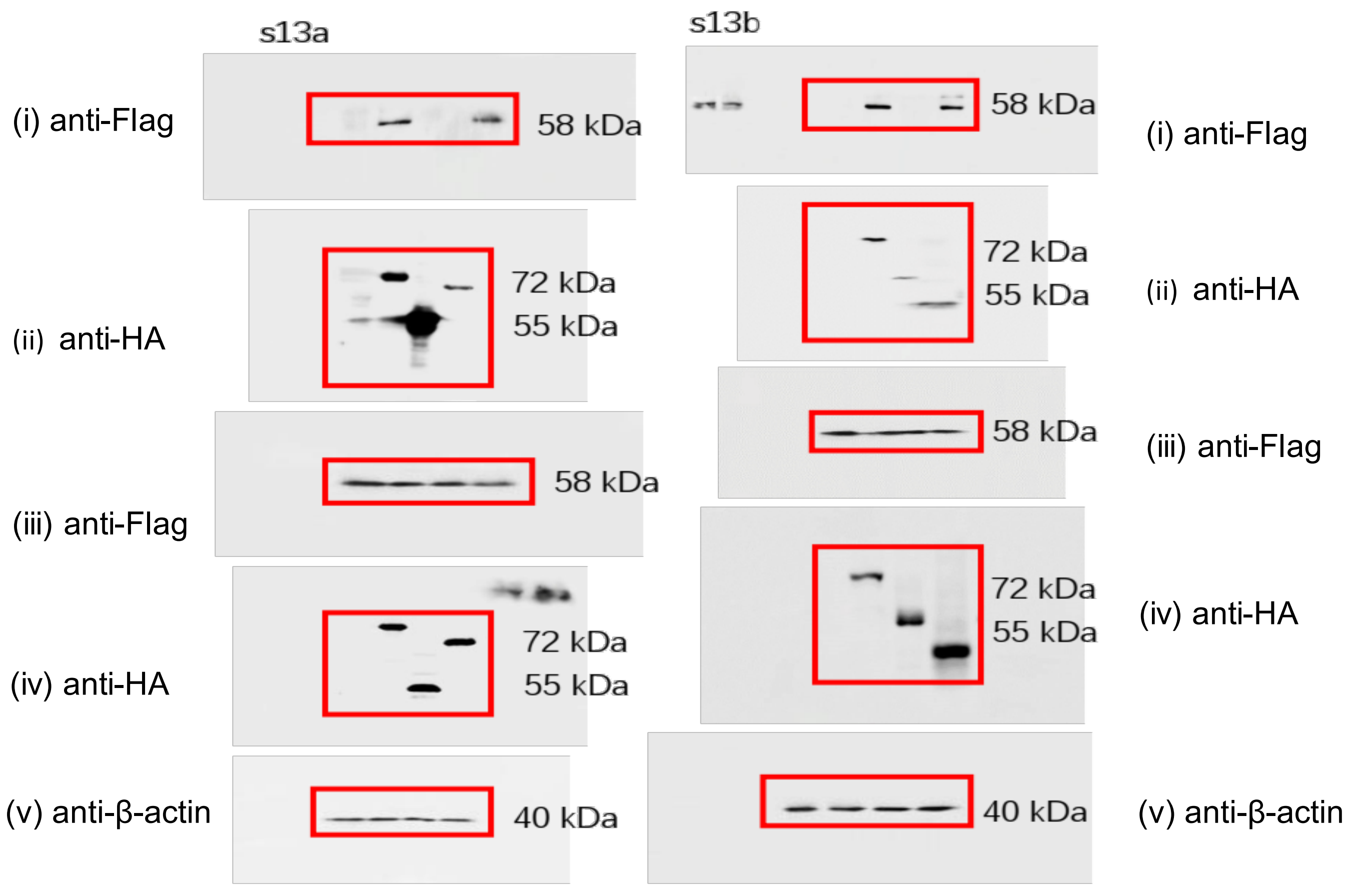

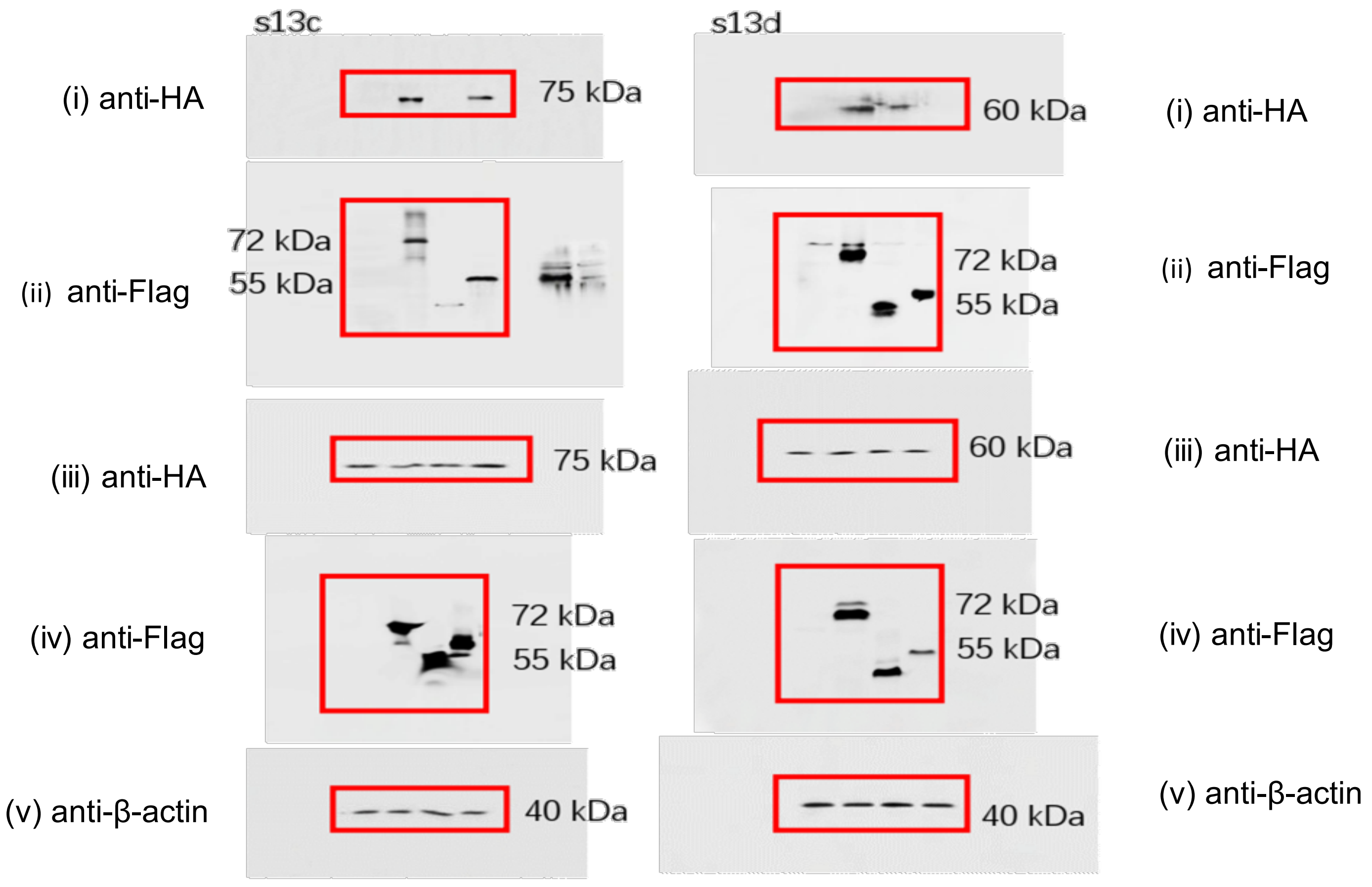

Supplement: Supplementary file 1 — Supplementary Information [file 41467_2023_41028_MOESM1_ESM.pdf]
