# Supplementary material for: MAVS integrates glucose metabolism and RIG-I-like receptor signaling
Source: Nat Commun. 2023 Sep 2;14:5343. doi: 10.1038/s41467-023-41028-9 (PMC10475032; doi:10.1038/s41467-023-41028-9)
Supplement: Supplementary file 3 — Description of Additional Supplementary Files [file 41467_2023_41028_MOESM3_ESM.pdf]

Description of Additional Supplementary Files

File name: Supplementary Data 1

Description: $^{13}C_6$-glucose tracing glycolytic metabolites after VSV stimulation. Related to Figure 1b-f.

File name: Supplementary Data 2

Description: $^{13}C_6$-glucose tracing glycolytic metabolites after transfected with a control vector or indicated MAVS alleles. Related to Figure 3a-3e.

File name: Supplementary Data 3

Description: 1,2-^{13}C-glucose tracing glycolytic metabolites, PPP, nucleotides, and TCA cycle after VSV stimulation. Related to Supplementary Figure 1h-i.

File name: Supplementary Data 4

Description: γ-^{15}N -glucose tracing glycolytic metabolites in different cell type after VSV or poly(I:C) stimulation. Related to Supplementary Figure 2b-2j.

File name: Supplementary Data 5

Description: 1,2-^{13}C-glucose tracing glycolytic metabolites, PPP, nucleotides, and TCA cycle after transfected with a control vector or indicated MAVS alleles. Related to Supplementary Figure 5a-5i.

File name: Supplementary Data 6

Description: γ-^{15}N-glucose tracing metabolites after transfected with a control vector or indicated MAVS alleles. Related to Supplementary Figure 6a-6b.

File name: Supplementary Data 7

Description: γ-^{15}N-glucose tracing metabolites after transfected and stimulation. Related to Supplementary Figure 15a-15g.
