# Supplementary material for: MAVS integrates glucose metabolism and RIG-I-like receptor signaling
Source: Nat Commun. 2023 Sep 2;14:5343. doi: 10.1038/s41467-023-41028-9 (PMC10475032; doi:10.1038/s41467-023-41028-9)
Supplement: Supplementary file 12 — Source Data [file 41467_2023_41028_MOESM12_ESM.pdf]

Fig1b: upper glycolysis metabolism

			M0					M6				
			1	2	3	AVE	STDEV	1	2	3	AVE	STDEV
G6P	wt	Ctrl	25.39	28.64	32.07	28.70	3.34	70.15	73.11	72.64	71.97	1.59
		VSV	5.33	12.16	4.18	7.22	4.31	92.35	94.11	90.17	92.21	1.97
	mamv-/-	Ctrl	28.29	30.14	32.74	30.39	2.24	70.16	68.17	72.15	70.16	1.99
		VSV	27.19	29.36	31.24	29.26	2.03	67.26	71.28	73.66	70.73	3.23
F6P	wt	Ctrl	25.39	27.18	29.33	27.30	1.97	73.26	75.26	77.09	75.20	1.92
		VSV	3.08	15.09	7.11	8.43	6.11	92.14	94.77	96.26	94.39	2.09
	mamv-/-	Ctrl	28.39	29.31	30.74	29.48	1.18	70.26	72.19	75.96	72.80	2.90
		VSV	27.44	29.06	32.61	29.70	2.64	71.84	74.99	72.05	72.96	1.76
F1,6P	wt	Ctrl	13.91	15.08	17.01	15.33	1.57	83.26	85.91	84.17	84.45	1.35
		VSV	49.26	48.33	51.22	49.60	1.48	53.21	55.09	50.32	52.87	2.40
	mamv-/-	Ctrl	19.62	20.37	18.77	19.59	0.80	80.26	81.24	82.99	81.50	1.38
		VSV	18.09	20.36	21.44	19.96	1.71	81.74	83.61	82.19	82.51	0.98

Fig1c: lower glycolysis metabolism

			M0					M1					M2					M3				
			1	2	3	AVE	STDEV	1	2	3	AVE	STDEV	1	2	3	AVE	STDEV	1	2	3	AVE	STDEV
2,3-PG	wt	Ctrl	28.19	29.07	31.71	29.66	1.83	/	/	/	/	/	/	/	/	/	/	72.36	70.16	74.16	72.23	2.00
		VSV	57.29	54.29	59.11	56.90	2.43	/	/	/	/	/	/	/	/	/	/	42.38	44.95	47.27	44.87	2.45
	mamv-/-	Ctrl	25.39	22.17	27.09	24.88	2.50	/	/	/	/	/	/	/	/	/	/	73.69	77.25	72.36	74.43	2.53
		VSV	27.26	26.33	24.07	25.89	1.64	/	/	/	/	/	/	/	/	/	/	70.36	72.68	74.11	72.38	1.89
Pyruvate	wt	Ctrl	31.26	28.36	30.17	29.93	1.46	5.26	6.38	4.26	5.30	1.06	5.33	4.11	4.26	4.57	0.67	63.14	60.14	63.26	62.18	1.77
		VSV	62.85	63.11	67.19	64.38	2.43	4.18	6.35	4.15	4.89	1.26	5.26	6.98	3.15	5.13	1.92	31.26	28.77	25.94	28.66	2.66
	mamv-/-	Ctrl	30.28	22.19	32.66	28.38	5.49	3.05	7.26	4.18	4.83	2.18	5.18	6.36	4.11	5.22	1.13	65.95	64.18	63.26	64.46	1.37
		VSV	25.39	28.19	27.44	27.01	1.45	5.39	6.33	4.47	5.40	0.93	6.33	4.08	4.71	5.04	1.16	60.74	62.37	65.22	62.78	2.27
Lactate	wt	Ctrl	34.25	51.26	45.28	43.60	8.63	4.25	9.36	1.25	4.95	4.10	5.36	1.05	6.39	4.27	2.83	43.05	45.50	50.89	46.48	4.01
		VSV	68.94	62.36	71.29	67.53	4.63	3.05	7.15	4.15	4.78	2.12	4.28	3.06	7.26	4.87	2.16	18.28	21.85	27.63	22.59	4.72
	mamv-/-	Ctrl	55.96	44.18	47.92	49.35	6.02	5.96	3.36	1.36	3.56	2.31	4.15	2.06	6.37	4.19	2.16	40.96	44.95	49.33	45.08	4.19
		VSV	44.96	50.39	51.33	48.89	3.44	4.18	6.38	3.26	4.61	1.60	2.09	8.99	1.06	4.05	4.31	41.93	47.26	50.36	46.52	4.26

Fig1d: PPP metabolism

			M0					M6				
			1	2	3	AVE	STDEV	1	2	3	AVE	STDEV
6PG	wt	Ctrl	92.06	95.38	88.36	91.93	3.51	5.26	12.36	7.01	8.21	3.70
		VSV	78.29	73.26	85.11	78.89	5.95	23.25	28.36	20.18	23.93	4.13
	mamv-/-	Ctrl	96.35	90.36	89.30	92.00	3.80	9.36	2.38	7.26	6.33	3.58
		VSV	92.11	96.35	94.04	94.17	2.12	4.26	6.33	7.02	5.87	1.44
			M0					M4				
			1	2	3	AVE	STDEV	1	2	3	AVE	STDEV
S7P	wt	Ctrl	50.26	59.35	66.38	58.66	8.08	43.29	47.95	42.36	44.53	3.00
		VSV	26.35	30.41	22.58	26.45	3.92	72.36	75.19	77.95	75.17	2.80
	mamv-/-	Ctrl	52.36	55.97	58.36	55.56	3.02	45.26	47.95	40.39	44.53	3.83
		VSV	50.86	53.77	56.08	53.57	2.62	46.93	51.24	48.36	48.84	2.20

Fig1e: HBP metabolism

			M0					M6					M11				
			1	2	3	AVE	STDEV	1	2	3	AVE	STDEV	1	2	3	AVE	STDEV
UDP-GlcNac	wt	Ctrl	21.25	23.65	17.25	20.72	3.23	53.26	49.14	48.36	50.25	2.63	32.96	28.19	33.02	31.39	2.77
		VSV	1.26	3.36	2.25	2.29	1.05	55.26	63.22	64.26	60.91	4.92	47.28	43.66	42.36	44.43	2.55
	mamv-/-	Ctrl	22.36	24.15	16.02	20.84	4.27	51.74	47.27	53.06	50.69	3.03	36.26	24.18	32.69	31.04	6.21
		VSV	21.33	17.18	29.07	22.53	6.03	52.74	54.15	46.01	50.97	4.35	25.75	34.22	33.17	31.05	4.62

Fig1f: TCA cycle metabolism

			M0					M1									
			1	2	3	AVE	STDEV	1	2	3	AVE	STDEV					
aKG	wt	Ctrl	78.47	80.25	86.14	81.62	4.01	19.36	22.38	14.96	18.90	3.73					
		VSV	95.38	93.24	91.07	93.23	2.16	4.26	9.37	7.25	6.96	2.57					
	mamv-/-	Ctrl	82.36	76.15	84.25	80.92	4.24	15.97	23.05	21.71	20.24	3.76					
		VSV	87.54	82.38	74.24	81.39	6.71	18.09	16.34	24.07	19.50	4.05					
Succinate	wt	Ctrl	78.25	88.04	80.36	82.22	5.15	15.26	21.08	17.63	17.99	2.93					
		VSV	96.35	94.14	92.35	94.28	2.00	4.26	3.11	10.74	6.04	4.11					
	mamv-/-	Ctrl	73.36	77.18	82.33	77.62	4.50	22.38	19.36	20.04	20.59	1.58					
		VSV	71.17	75.28	85.08	77.18	7.15	23.74	18.26	21.33	21.11	2.75					
			M0					M2					M4				
			1	2	3	AVE	STDEV	1	2	3	AVE	STDEV	1	2	3	AVE	STDEV
Citrate	wt	Ctrl	68.96	55.24	60.74	61.65	6.90	23.86	29.36	35.26	29.49	5.70	8.26	12.36	14.11	11.58	3.00
		VSV	80.36	88.96	85.22	84.85	4.31	12.36	15.96	18.96	15.76	3.30	4.26	3.26	1.05	2.86	1.64
	mamv-/-	Ctrl	67.26	63.34	65.85	65.48	1.99	30.28	27.96	25.33	27.86	2.48	7.28	14.26	10.31	10.62	3.50
		VSV	70.15	63.25	61.74	65.05	4.48	31.75	26.39	25.74	27.96	3.30	6.78	11.25	15.96	11.33	4.59

Fig2a: mRNA level							
			1	2	3	AVE	STDEV
Glut1	wt	Ctrl	1.00	1.36	3.84	2.07	1.55
		VSV	5.54	6.36	4.25	5.38	1.06
	mamv-/-	Ctrl	0.96	1.24	0.83	1.01	0.21
		VSV	1.26	1.99	1.75	1.67	0.37
Glut4	wt	Ctrl	1.00	0.95	1.11	1.02	0.08
		VSV	6.74	7.07	10.77	8.19	2.24
	mamv-/-	Ctrl	1.07	1.39	0.93	1.13	0.24
		VSV	2.33	3.25	2.71	2.76	0.46

Fig2b: HK activity					
	1	2	3	AVE	STDEV
Ctrl	1.00	1.05	0.94	1.00	0.06
MAVS	1.56	1.48	1.68	1.57	0.10
sh-Ctrl	1.00	1.05	0.93	0.99	0.06
sh-MAVS	0.54	0.47	0.63	0.55	0.08

Fig2c: Pyruvate level							
			1	2	3	AVE	STDEV
Pyruvate	wt	Ctrl	1.00	0.95	1.14	1.03	0.10
		VSV	0.63	0.57	0.61	0.60	0.03
	mamv-/-	Ctrl	1.17	1.02	0.95	1.05	0.11
		VSV	0.97	0.99	1.06	1.01	0.05

Fig2d: Lactate level							
			1	2	3	AVE	STDEV
Lactate	wt	Ctrl	1.00	0.96	1.07	1.01	0.06
		VSV	0.53	0.57	0.63	0.58	0.05
	mamv-/-	Ctrl	0.99	1.06	1.11	1.05	0.06
		VSV	0.95	1.02	1.09	1.02	0.07

Fig2e: Succinate level							
			1	2	3	AVE	STDEV
Succinate	wt	Ctrl	1.00	0.95	1.07	1.01	0.06
		VSV	0.73	0.77	0.68	0.73	0.05
	mamv-/-	Ctrl	1.03	0.91	0.89	0.94	0.08
		VSV	1.14	0.95	0.93	1.01	0.12

Fig2f: G6PD activity							
			1	2	3	AVE	STDEV
G6PD	wt	Ctrl	1.00	0.93	1.13	1.02	0.10
		VSV	2.03	2.14	1.83	2.00	0.16
	mamv-/-	Ctrl	0.95	1.06	1.02	1.01	0.06
		VSV	0.93	0.98	1.09	1.00	0.08

Fig2h: NADPH level							
			1	2	3	AVE	STDEV
NADPH	wt	Ctrl	1.00	0.96	1.04	1.00	0.04
		VSV	1.58	1.63	1.44	1.55	0.10
	mamv-/-	Ctrl	0.95	0.93	1.09	0.99	0.09
		VSV	1.02	1.06	1.10	1.06	0.04

Fig 2g:

(i)-(iii): Because of similar molecular weight, the same samples were run on two gels and the membranes were incubated with the indicated antibodies.

Fig 2g

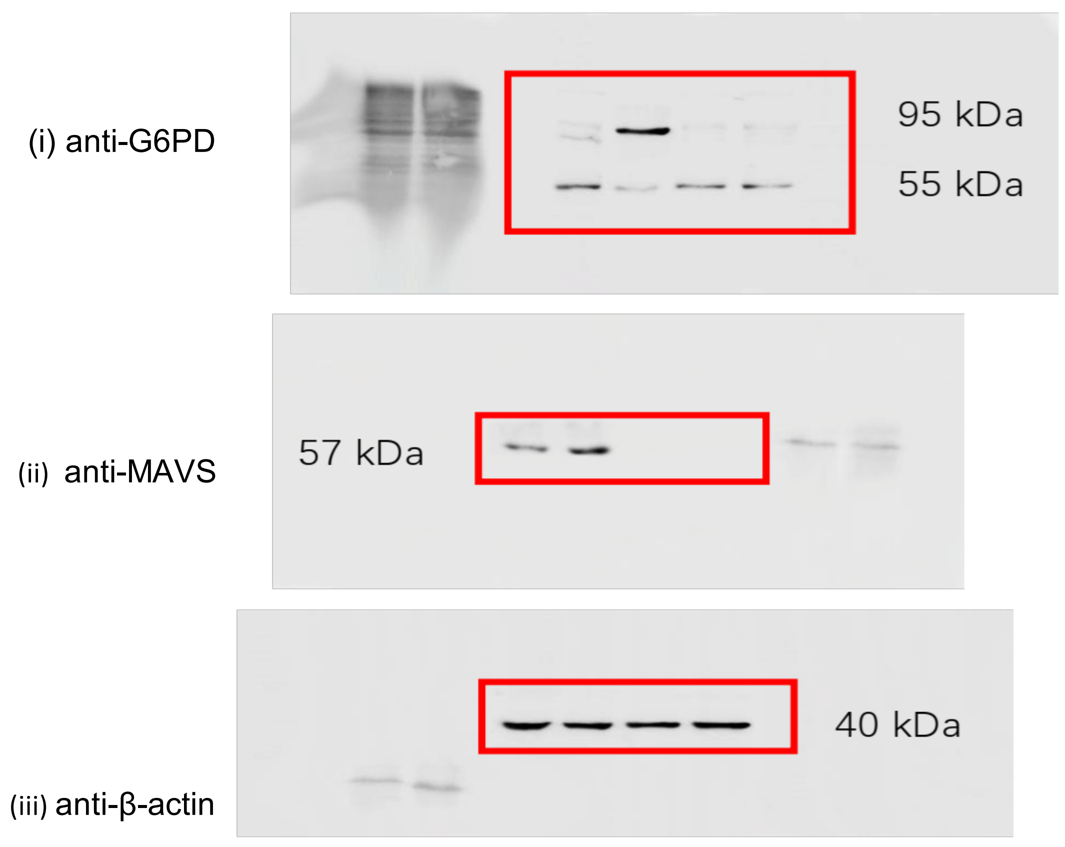

Fig2i: NADP+/NADPH							
			1	2	3	AVE	STDEV
NADP+/NADPH	wt	Ctrl	1.00	0.86	1.24	1.03	0.19
		VSV	0.54	0.47	0.59	0.53	0.06
	mamv-/-	Ctrl	1.05	0.93	0.91	0.96	0.08
		VSV	1.08	0.96	0.92	0.99	0.08

Fig2j: mRNA level							
			1	2	3	AVE	STDEV
GFPT1	wt	Ctrl	1.00	1.26	1.48	1.25	0.24
		VSV	1.35	0.95	0.84	1.05	0.27
	mamv-/-	Ctrl	1.47	1.53	0.74	1.25	0.44
		VSV	0.87	1.38	1.77	1.34	0.45
GFPT2	wt	Ctrl	1.00	1.17	0.89	1.02	0.14
		VSV	6.35	7.18	4.09	5.87	1.60
	mamv-/-	Ctrl	0.95	1.16	0.83	0.98	0.17
		VSV	1.24	0.87	0.93	1.01	0.20

Fig2k: UDP-GlcNac level							
			1	2	3	AVE	STDEV
UDP-GlcNac	wt	Ctrl	1	1.15	0.83	0.99	0.16
		VSV	1.67	1.79	1.85	1.77	0.09
	mamv-/-	Ctrl	0.94	0.89	1.19	1.01	0.16
		VSV	0.86	0.92	0.99	0.92	0.07

Fig3a: upper glycolysis metabolism

		M0					M6				
		1	2	3	AVE	STDEV	1	2	3	AVE	STDEV
G6P	ctrl	32.06	28.26	34.55	31.62	3.17	69.68	65.26	67.25	67.40	2.21
	cyto	27.15	29.33	35.04	30.51	4.07	64.26	68.02	70.26	67.51	3.03
	wt	8.36	5.29	7.18	6.94	1.55	92.14	96.35	90.58	93.02	2.98
	mito	12.63	7.26	9.34	9.74	2.71	85.26	88.93	94.26	89.48	4.53
	pex	53.29	58.69	52.36	54.78	3.42	41.25	48.59	46.35	45.40	3.76
	mam	32.04	25.99	27.91	28.65	3.09	72.26	68.27	65.33	68.62	3.48
F6P	ctrl	27.18	30.96	24.85	27.66	3.08	74.25	78.29	70.62	74.39	3.84
	cyto	24.99	28.17	33.11	28.76	4.09	68.29	75.66	74.92	72.96	4.06
	wt	5.74	7.29	8.33	7.12	1.30	92.58	96.38	95.17	94.71	1.94
	mito	11.04	6.89	13.05	10.33	3.14	86.35	88.92	94.27	89.85	4.04
	pex	53.29	57.18	59.33	56.60	3.06	42.69	44.85	47.95	45.16	2.64
	mam	57.25	63.04	54.77	58.35	4.24	45.04	47.26	40.36	44.22	3.52
F1,6P	ctrl	30.47	34.28	28.33	31.03	3.01	68.25	70.29	62.96	67.17	3.78
	cyto	27.26	31.55	39.74	32.85	6.34	65.74	69.04	61.84	65.54	3.60
	wt	5.29	7.20	3.17	5.22	2.02	92.85	95.74	96.33	94.97	1.86
	mito	10.27	8.29	7.14	8.57	1.58	84.27	93.24	90.77	89.43	4.63
	pex	58.36	52.17	55.02	55.18	3.10	40.25	42.96	37.25	40.15	2.86
	mam	60.85	57.04	54.11	57.33	3.38	45.26	33.68	38.26	39.07	5.83

Fig3b: lower glycolysis metabolism

		M0					M6				
		1	2	3	AVE	STDEV	1	2	3	AVE	STDEV
2,3-PG	ctrl	35.29	36.19	30.11	33.86	3.28	62.57	65.11	70.14	65.94	3.85
	cyto	31.78	36.26	37.89	35.31	3.16	65.08	69.24	60.24	64.85	4.50
	wt	9.28	11.47	14.57	11.77	2.66	92.15	88.25	83.26	87.89	4.46
	mito	14.57	18.26	11.36	14.73	3.45	82.36	87.15	80.15	83.22	3.58
	pex	62.24	68.14	63.78	64.72	3.06	32.25	39.35	30.57	34.06	4.66
	mam	72.78	65.34	67.18	68.43	3.88	28.24	25.18	31.25	28.22	3.04

		M0					M1					M2					M3				
		1	2	3	AVE	STDEV	1	2	3	AVE	STDEV	1	2	3	AVE	STDEV	1	2	3	AVE	STDEV
Pyruvate	ctrl	38.25	34.17	30.38	34.27	3.94	2.71	6.98	8.06	5.92	2.83	4.25	2.36	9.91	5.51	2.57	49.68	52.36	57.08	53.04	3.75
	cyto	37.18	35.22	31.05	34.48	3.13	1.57	8.27	6.33	5.39	3.45	4.27	5.56	6.33	5.39	1.04	54.11	50.36	57.91	54.13	3.78
	wt	8.47	2.58	3.08	4.71	3.27	3.04	2.87	8.60	4.84	3.26	5.36	7.95	2.33	5.21	2.81	82.57	89.36	81.33	84.42	4.32
	mito	11.74	15.96	13.04	13.58	2.16	4.26	5.33	7.91	5.83	1.88	2.74	6.38	7.09	5.40	2.33	73.29	75.26	70.81	73.12	2.23
	pex	65.28	69.33	61.01	65.21	4.16	1.09	7.28	8.23	5.53	3.88	4.18	5.69	5.93	5.27	0.95	24.17	20.96	26.33	23.82	2.70
	mam	62.96	68.18	63.6	64.91	2.85	3.57	5.96	6.91	5.48	1.72	4.05	7.88	8.24	6.72	2.32	23.37	26.35	18.24	22.65	4.10
Lactate	ctrl	33.28	34.96	39.36	35.87	3.14	4.26	2.36	8.26	4.96	3.01	2.36	8.11	7.09	5.85	3.07	52.38	58.68	50.28	53.78	4.37
	cyto	30.96	38.25	36.31	35.17	3.78	2.36	7.26	6.24	5.29	2.59	1.25	5.36	7.99	4.87	3.40	53.98	55.07	59.36	56.14	2.84
	wt	5.36	9.35	2.07	5.59	3.65	8.26	4.25	2.36	4.96	3.01	1.98	6.38	7.12	5.16	2.78	82.57	80.33	87.69	83.53	3.77
	mito	1.07	6.35	4.27	3.90	2.66	2.14	3.69	8.92	4.92	3.55	4.28	3.26	7.18	4.91	2.03	88.29	87.96	83.29	86.51	2.80
	pex	63.26	70.14	67.92	67.11	3.51	1.70	5.26	6.75	4.57	2.59	4.28	6.74	8.11	6.38	1.94	21.36	23.88	26.91	24.05	2.78
	mam	62.05	66.35	71.05	66.48	4.50	5.69	6.39	9.15	7.08	1.83	4.38	7.15	6.31	5.95	1.42	20.17	24.14	23.66	22.66	2.17

Fig3c: TCA cycle metabolism

		M0					M2				
		1	2	3	AVE	STDEV	1	2	3	AVE	STDEV
αKG	ctrl	80.26	84.26	82.35	82.29	2.00	10.96	21.36	14.25	15.52	5.32
	cyto	88.17	82.06	83.09	84.44	3.27	9.36	14.25	20.36	14.66	5.51
	wt	72.85	60.47	68.29	67.20	6.26	35.96	23.85	31.26	30.36	6.11
	mito	55.26	61.25	62.37	59.63	3.82	32.89	37.25	44.47	38.20	5.85
	pex	89.26	95.07	97.36	93.90	4.18	1.74	3.29	6.36	3.80	2.35
	mam	96.39	90.04	92.55	92.99	3.20	4.27	2.96	2.33	3.19	0.99
Succinate	ctrl	86.39	93.06	81.27	86.91	5.91	12.93	8.29	15.47	12.23	3.64
	cyto	92.17	88.29	79.14	86.53	6.69	10.92	17.26	13.26	13.81	3.21
	wt	68.29	62.25	70.26	66.93	4.17	32.06	28.19	36.29	32.18	4.05
	mito	64.25	57.26	63.95	61.82	3.95	36.39	33.96	47.14	39.16	7.01
	pex	93.25	97.05	91.74	94.01	2.74	6.35	7.26	4.25	5.95	1.54
	mam	95.36	88.19	92.52	92.02	3.61	3.08	6.35	7.18	5.54	2.17

		M0					M2					M4				
		1	2	3	AVE	STDEV	1	2	3	AVE	STDEV	1	2	3	AVE	STDEV
Citrate	ctrl	65.26	68.92	77.14	70.44	6.08411	23.59	22.92	29.36	25.29	3.54	6.35	11.74	2.36	6.82	4.71
	cyto	72.05	69.33	73.91	71.76333	2.303418	19.38	24.15	24.96	22.83	3.02	8.36	1.95	13.71	8.01	5.89
	wt	22.36	23.55	29.36	25.09	3.745491	62.28	45.19	50.29	52.59	8.77	29.38	21.26	23.69	24.78	4.17
	mito	27.26	31.92	25.78	28.32	3.20431	49.05	43.26	47.95	46.75	3.07	24.25	28.33	25.97	26.18	2.05
	pex	86.39	89.29	85.35	87.01	2.041862	8.87	13.69	11.95	11.50	2.44	4.98	2.39	2.36	3.24	1.50
	mam	82.68	84.98	86.19	84.61667	1.782984	10.98	13.58	16.98	13.85	3.01	2.38	5.29	1.68	3.12	1.91

Fig3d: HBP metabolism

		M0					M6				
		1	2	3	AVE	STDEV	1	2	3	AVE	STDEV
GlcN-6P	ctrl	53.69	58.29	50.41	54.13	3.96	42.36	44.96	47.25	44.86	2.45
	cyto	55.09	52.31	57.08	54.83	2.40	49.05	40.25	43.26	44.19	4.47
	wt	32.05	29.35	24.69	28.70	3.72	75.26	70.26	71.24	72.25	2.65
	mito	51.22	58.36	57.14	55.57	3.82	38.29	46.39	48.02	44.23	5.21
	pex	53.02	52.96	59.36	55.11	3.68	39.26	47.02	46.33	44.20	4.29
	mam	25.36	20.36	28.41	24.71	4.06	68.29	75.29	77.29	73.62	4.73
GlcNAc-6P	ctrl	53.22	57.26	51.08	53.85	3.14	32.14	48.26	51.26	43.89	10.28
	cyto	55.39	59.36	50.74	55.16	4.31	42.16	37.95	47.26	42.46	4.66
	wt	12.36	18.29	14.51	15.05	3.00	78.26	82.35	85.36	81.99	3.56
	mito	53.95	49.6	58.26	53.94	4.33	41.96	45.85	47.09	44.97	2.68
	pex	54.27	58.29	48.69	53.75	4.82	38.92	44.84	49.35	44.37	5.23
	mam	20.35	18.47	16.33	18.38	2.01	80.68	83.29	76.39	80.12	3.48
GlcNAc-1P	ctrl	52.36	57.18	60.35	56.63	4.02	46.96	38.36	41.85	42.39	4.33
	cyto	61.24	50.47	52.11	54.61	5.80	43.29	45.36	40.11	42.92	2.64
	wt	21.04	19.38	16.33	18.92	2.39	78.29	75.36	82.37	78.67	3.52
	mito	61.24	51.24	53.26	55.25	5.29	43.68	39.58	46.77	43.34	3.61
	pex	59.36	52.35	54.11	55.27	3.65	38.22	42.68	46.31	42.40	4.05
	mam	15.24	21.25	11.07	15.85	5.12	83.26	80.69	85.26	83.07	2.29

		M0					M6					M11				
		1	2	3	AVE	STDEV	1	2	3	AVE	STDEV	1	2	3	AVE	STDEV
UDP-GlcNA	ctrl	32.08	36.95	42.35	37.13	5.14	31.22	37.24	31.04	33.17	3.53	29.35	20.25	32.14	27.25	6.22
	cyto	41.99	32.69	35.78	36.82	4.74	32.32	37.18	35.04	34.85	2.44	23.05	31.27	22.91	25.74	4.79
	wt	3.25	6.87	2.95	4.36	2.18	51.74	46.33	48.19	48.75	2.75	42.35	44.15	47.04	44.51	2.37
	mito	33.89	36.95	30.47	33.77	3.24	27.18	36.36	31.04	31.53	4.61	20.39	38.57	37.66	32.21	10.24
	pex	41.25	34.78	38.44	38.16	3.24	31.33	32.14	21.78	28.42	5.76	21.09	37.25	36.93	31.76	9.24
	mam	8.19	3.69	4.71	5.53	2.36	47.29	44.11	45.96	45.79	1.60	43.36	45.96	50.05	46.46	3.37

Fig3e: PPP metabolism

		M0					M6				
		1	2	3	AVE	STDEV	1	2	3	AVE	STDEV
6PG	ctrl	78.69	75.24	80.28	78.07	2.58	21.25	17.28	23.71	20.75	3.24
	cyto	73.29	79.38	75.17	75.95	3.12	24.68	27.25	20.27	24.07	3.53
	wt	53.29	57.18	60.17	56.88	3.45	42.58	47.28	40.29	43.38	3.56
	mito	82.36	86.29	81.26	83.30	2.64	17.28	15.69	13.05	15.34	2.14
	pex	53.26	46.92	40.24	46.81	6.51	51.07	57.29	49.18	52.51	4.24
	mam	84.27	76.92	80.17	80.45	3.68	20.39	22.74	17.29	20.14	2.73

		M0					M4				
		1	2	3	AVE	STDEV	1	2	3	AVE	STDEV
S7P	ctrl	43.05	48.25	34.29	41.86	7.06	56.39	58.35	62.04	58.93	2.87
	cyto	40.17	43.11	39.24	40.84	2.02	62.32	59.24	56.32	59.29	3.00
	wt	83.26	89.36	80.26	84.29	4.64	17.25	12.38	19.36	16.33	3.58
	mito	38.26	35.29	41.26	38.27	2.99	66.26	59.36	62.28	62.63	3.46
	pex	80.36	78.26	75.26	77.96	2.56	22.36	26.38	18.04	22.26	4.17
	mam	42.39	47.26	40.96	43.54	3.30	56.39	52.06	59.31	55.92	3.65

Fig4b: mRNA level

			1	2	3	AVE	STDEV
IFN-β	Medium	Ctrl	1.00	8.26	4.18	4.48	3.64
		VSV	359.65	452.05	384.22	398.64	47.86
	G6PDi	Ctrl	2.96	7.18	12.09	7.41	4.57
		VSV	367.29	412.99	346.28	375.52	34.11
IFN-λ1	Medium	Ctrl	1.85	3.85	9.24	4.98	3.82
		VSV	136.29	125.96	184.22	148.82	31.09
	G6PDi	Ctrl	2.36	4.28	11.04	5.89	4.56
		VSV	15.27	8.92	23.04	15.74	7.07
IL6	Medium	Ctrl	1.00	5.28	7.15	4.48	3.15
		VSV	263.14	207.93	227.85	232.97	27.96
	G6PDi	Ctrl	7.89	11.95	4.82	8.22	3.58
		VSV	56.38	78.14	31.08	55.20	23.55
TNFα	Medium	Ctrl	1.00	6.77	16.35	8.04	7.75
		VSV	85.24	104.28	66.84	85.45	18.72
	G6PDi	Ctrl	14.25	10.26	7.41	10.64	3.44
		VSV	26.35	36.17	45.27	35.93	9.46
IL-1β	Medium	Ctrl	1.00	7.15	4.25	4.13	3.08
		VSV	128.68	162.77	217.09	169.51	44.59
	G6PDi	Ctrl	3.26	7.15	11.04	7.15	3.89
		VSV	59.35	42.15	33.09	44.86	13.34

Fig4c: mRNA level

			1	2	3	AVE	STDEV
IFN-β	Medium	Ctrl	1.00	5.95	2.33	3.09	2.56
		VSV	418.36	432.02	386.22	412.20	23.51
	G6PDi	Ctrl	2.69	7.22	4.15	4.69	2.31
		VSV	125.63	141.02	165.22	143.96	19.96
IFN-λ1	Medium	Ctrl	1.00	1.68	3.36	2.01	1.21
		VSV	135.26	148.26	169.33	150.95	17.19
	G6PDi	Ctrl	4.52	1.98	8.24	4.91	3.15
		VSV	147.29	130.25	127.92	135.15	10.58
IL6	Medium	Ctrl	1.00	2.36	5.36	2.91	2.23
		VSV	214.25	235.63	245.36	231.75	15.91
	G6PDi	Ctrl	4.25	3.69	7.78	5.24	2.22
		VSV	7.26	23.64	14.27	15.06	8.22
TNFα	Medium	Ctrl	1.00	6.39	14.27	7.22	6.67
		VSV	81.24	102.36	75.36	86.32	14.20
	G6PDi	Ctrl	12.36	9.14	14.25	11.92	2.58
		VSV	33.28	12.35	23.68	23.10	10.48
IL-1β	Medium	Ctrl	1.00	3.36	4.25	2.87	1.68
		VSV	135.74	154.29	169.33	153.12	16.83
	G6PDi	Ctrl	5.26	9.35	7.33	7.31	2.05
		VSV	12.54	23.85	17.24	17.88	5.68

Fig4d: ifn-β mRNA level

		1	2	3	AVE	STDEV
ui	Medium	0.00	0.00	0.00	0.00	0.00
	G6PDi	0.00	0.00	0.00	0.00	0.00
ctrl	Medium	1.00	1.03	1.13	2.38	0.55
	G6PDi	1.22	1.06	0.95	2.39	0.62
cyto	Medium	2.35	1.85	2.95	12.26	2.01
	G6PDi	1.69	2.85	2.63	13.49	1.77
wt	Medium	12.06	10.36	14.36	6.42	1.40
	G6PDi	15.36	13.25	11.85	6.25	1.06
mito	Medium	6.35	5.06	7.86	3.60	0.55
	G6PDi	7.26	6.35	5.15	6.25	1.06
pex	Medium	3.58	4.15	3.06	3.60	0.55
	G6PDi	3.95	4.48	2.86	3.76	0.83
mam	Medium	2.96	3.26	2.06	2.76	0.62
	G6PDi	2.14	2.42	2.88	2.48	0.37

Fig4e: ifn-λ1 mRNA level

		1	2	3	AVE	STDEV
ui	Medium	0.00	0.00	0.00	0.00	0.00
	G6PDi	0.00	0.00	0.00	0.00	0.00
ctrl	Medium	1.00	1.26	1.09	1.12	0.13
	G6PDi	0.86	0.73	1.96	1.18	0.68
cyto	Medium	1.25	0.86	0.77	0.96	0.26
	G6PDi	0.92	0.88	1.75	1.18	0.49
wt	Medium	6.95	7.69	9.47	8.04	1.30
	G6PDi	1.28	2.68	1.74	1.90	0.71
mito	Medium	3.68	4.85	3.77	4.10	0.65
	G6PDi	3.05	4.98	3.82	3.95	0.97
pex	Medium	5.36	4.78	6.98	5.71	1.14
	G6PDi	1.65	1.18	0.96	1.26	0.35
mam	Medium	1.25	0.86	1.49	1.20	0.32
	G6PDi	0.93	1.69	0.88	1.17	0.45

Fig4f: il-6 mRNA level

		1	2	3	AVE	STDEV
ui	Medium	0.00	0.00	0.00	0.00	0.00
	G6PDi	0.00	0.00	0.00	0.00	0.00
ctrl	Medium	1.25	1.00	1.09	1.11	0.13
	G6PDi	0.91	1.57	1.22	1.23	0.33
cyto	Medium	1.35	0.78	1.69	1.27	0.46
	G6PDi	0.71	0.69	1.89	1.10	0.69
wt	Medium	12.63	10.25	9.35	10.74	1.69
	G6PDi	4.26	3.25	5.96	4.49	1.37
mito	Medium	9.35	7.25	4.98	7.19	2.19
	G6PDi	4.06	9.15	7.89	7.03	2.65
pex	Medium	5.36	4.15	3.29	4.27	1.04
	G6PDi	2.05	1.47	2.11	1.88	0.35
mam	Medium	11.35	8.26	9.25	9.62	1.58
	G6PDi	10.47	9.02	9.79	9.76	0.73

Fig4g: tnf-α mRNA level

		1	2	3	AVE	STDEV
ui	Medium	0.00	0.00	0.00	0.00	0.00
	G6PDi	0.00	0.00	0.00	0.00	0.00
ctrl	Medium	1.74	1.00	0.89	1.21	0.46
	G6PDi	0.99	1.17	0.83	1.00	0.17
cyto	Medium	0.81	1.36	1.20	1.12	0.28
	G6PDi	0.79	0.96	1.33	1.03	0.28
wt	Medium	5.36	6.33	4.71	5.47	0.82
	G6PDi	1.96	2.36	1.77	2.03	0.30
mito	Medium	3.05	3.96	2.86	3.29	0.59
	G6PDi	4.25	3.06	2.55	3.29	0.87
pex	Medium	4.15	4.22	3.69	4.02	0.29
	G6PDi	1.74	1.19	1.23	1.39	0.31
mam	Medium	7.26	6.19	6.77	6.74	0.54
	G6PDi	8.86	7.47	5.44	7.26	1.72

Fig4h: il-1β mRNA level

		1	2	3	AVE	STDEV
ui	Medium	0.00	0.00	0.00	0.00	0.00
	G6PDi	0.00	0.00	0.00	0.00	0.00
ctrl	Medium	1.00	1.52	1.96	1.26	0.37
	G6PDi	0.86	1.74	1.35	0.96	0.26
cyto	Medium	1.65	0.91	1.22	8.29	1.96
	G6PDi	0.74	1.24	0.89	3.36	0.80
wt	Medium	8.26	10.26	6.35	5.13	0.92
	G6PDi	4.26	3.06	2.75	5.76	1.51
mito	Medium	5.26	4.15	5.98	5.00	0.63
	G6PDi	6.26	4.06	6.96	1.61	0.51
pex	Medium	5.59	4.33	5.09	11.12	1.68
	G6PDi	1.63	2.11	1.09	11.05	2.88
mam	Medium	9.38	11.25	12.74	11.12	1.68
	G6PDi	13.96	10.99	8.21	11.05	2.88

Fig4i: ifn-β mRNA level

		1	2	3	AVE	STDEV
ui	Medium	0.00	0.00	0.00	0.00	0.00
	Aza	0.00	0.00	0.00	0.00	0.00
ctrl	Medium	1.25	1.00	1.68	1.31	0.34
	Aza	0.88	1.68	1.74	1.43	0.48
cyto	Medium	2.14	2.62	2.05	2.27	0.31
	Aza	1.75	2.93	3.14	2.61	0.75
wt	Medium	11.85	9.26	8.04	9.72	1.95
	Aza	4.89	3.28	7.26	5.14	2.00
mito	Medium	6.25	5.17	5.03	5.48	0.67
	Aza	6.91	5.36	4.26	5.51	1.33
pex	Medium	3.27	3.96	4.15	3.79	0.46
	Aza	4.52	2.99	3.79	3.77	0.77
mam	Medium	3.95	2.75	2.66	3.12	0.72
	Aza	1.14	0.83	0.15	0.71	0.51

Fig4j: ifn-λ1 mRNA level

		1	2	3	AVE	STDEV
ui	Medium	0.00	0.00	0.00	0.00	0.00
	Aza	0.00	0.00	0.00	0.00	0.00
ctrl	Medium	1.58	1.00	1.06	1.21	0.32
	Aza	1.22	0.89	0.74	0.95	0.25
cyto	Medium	1.32	0.92	0.81	1.02	0.27
	Aza	1.17	0.81	0.77	0.92	0.22
wt	Medium	6.75	7.93	5.28	6.65	1.33
	Aza	6.31	6.78	5.96	6.35	0.41
mito	Medium	3.05	2.96	3.38	3.13	0.22
	Aza	3.78	2.86	2.74	3.13	0.57
pex	Medium	4.39	4.58	5.29	4.75	0.47
	Aza	5.38	5.24	3.08	4.57	1.29
mam	Medium	1.28	0.96	1.74	1.33	0.39
	Aza	0.82	1.18	1.64	1.21	0.41

Fig4k: il-6 mRNA level

		1	2	3	AVE	STDEV
ui	Medium	0.00	0.00	0.00	0.00	0.00
	Aza	0.00	0.00	0.00	0.00	0.00
ctrl	Medium	1.00	1.25	1.84	1.36	0.43
	Aza	0.86	1.99	1.03	1.29	0.61
cyto	Medium	0.84	1.16	1.29	1.10	0.23
	Aza	0.91	1.36	1.18	1.15	0.23
wt	Medium	12.06	9.35	9.63	10.35	1.49
	Aza	5.29	4.95	6.93	5.72	1.06
mito	Medium	6.34	5.29	7.15	6.26	0.93
	Aza	6.98	5.14	6.71	6.28	0.99
pex	Medium	5.27	4.96	5.96	5.40	0.51
	Aza	4.66	5.71	6.04	5.47	0.72
mam	Medium	11.75	12.96	13.25	12.65	0.80
	Aza	2.36	1.74	3.27	2.46	0.77

Fig4l: tnf-α mRNA level

		1	2	3	AVE	STDEV
ui	Medium	0.00	0.00	0.00	0.00	0.00
	Aza	0.00	0.00	0.00	0.00	0.00
ctrl	Medium	1.00	1.17	1.34	1.17	0.17
	Aza	0.87	1.14	1.52	1.18	0.33
cyto	Medium	1.05	0.79	1.17	1.00	0.19
	Aza	1.36	1.24	0.72	1.11	0.34
wt	Medium	5.26	4.24	5.97	5.16	0.87
	Aza	2.36	1.24	2.74	2.11	0.78
mito	Medium	3.29	3.74	4.15	3.73	0.43
	Aza	3.06	4.08	3.88	3.67	0.54
pex	Medium	3.18	4.24	3.06	3.49	0.65
	Aza	2.98	4.22	3.67	3.62	0.62
mam	Medium	7.29	8.18	6.38	7.28	0.90
	Aza	1.05	0.93	2.44	1.47	0.84

Fig4m: il-1β mRNA level

		1	2	3	AVE	STDEV
ui	Medium	0.00	0.00	0.00	0.00	0.00
	Aza	0.00	0.00	0.00	0.00	0.00
ctrl	Medium	1.00	1.14	1.36	1.17	0.18
	Aza	0.85	1.45	1.68	1.33	0.43
cyto	Medium	0.93	1.18	1.32	1.14	0.20
	Aza	0.87	1.04	1.34	1.08	0.24
wt	Medium	8.27	9.26	10.74	9.42	1.24
	Aza	4.25	3.14	3.77	3.72	0.56
mito	Medium	4.25	5.36	4.01	4.54	0.72
	Aza	3.87	4.57	4.69	4.38	0.44
pex	Medium	3.05	3.69	3.74	3.49	0.38
	Aza	2.89	3.24	4.12	3.42	0.63
mam	Medium	9.77	10.78	8.05	9.53	1.38
	Aza	1.46	2.37	2.58	2.14	0.60

Fig5c: VSV replication							
			1	2	3	AVE	STDEV
Lung	Medium	Ctrl	0.00	0.00	0.00	0.00	0.00
		VSV	100.00	106.33	101.05	102.46	3.39
	6-AN	Ctrl	0.00	0.00	0.00	0.00	0.00
		VSV	256.26	287.26	227.14	256.89	30.06

Fig5g: VSV replication							
			1	2	3	AVE	STDEV
Lung	Medium	Ctrl	0.00	0.00	0.00	0.00	0.00
		VSV	100.00	105.28	112.74	106.01	6.40
	Aza	Ctrl	0.00	0.00	0.00	0.00	0.00
		VSV	241.75	268.26	224.84	244.95	21.89

Fig5d: proinflammatory cytokines and IFN level							
			1	2	3	AVE	STDEV
IFN-α	Medium	Ctrl	1.96	8.69	24.51	11.72	11.58
		VSV	875.26	754.81	648.06	759.38	113.67
	6-AN	Ctrl	12.06	5.24	19.04	12.11	6.90
		VSV	775.96	647.26	692.34	705.19	65.30
IFN-β	Medium	Ctrl	5.28	9.33	17.25	10.62	6.09
		VSV	1274.26	1396.25	1308.95	1326.49	62.86
	6-AN	Ctrl	2.06	8.91	17.84	9.60	7.91
		VSV	1478.26	1214.96	1133.25	1275.49	180.29
IFN-λ	Medium	Ctrl	5.29	9.35	17.24	10.63	6.08
		VSV	586.36	715.24	507.95	603.18	104.66
	6-AN	Ctrl	11.74	24.81	6.04	14.20	9.62
		VSV	52.74	19.35	37.21	36.43	16.71
IL-6	Medium	Ctrl	2.86	15.99	23.74	14.20	10.55
		VSV	781.24	690.75	974.51	815.50	144.95
	6-AN	Ctrl	28.24	2.96	4.77	11.99	14.10
		VSV	224.62	349.36	267.21	280.40	63.41
IL-1β	Medium	Ctrl	5.29	16.35	7.15	9.60	5.92
		VSV	627.84	596.37	715.28	646.50	61.61
	6-AN	Ctrl	2.39	18.04	4.78	8.40	8.43
		VSV	289.36	247.62	272.36	269.78	20.99

Fig5h: proinflammatory cytokines and IFN level							
			1	2	3	AVE	STDEV
IFN-α	Medium	Ctrl	8.91	14.24	20.78	14.64	5.95
		VSV	814.27	689.26	972.04	825.19	141.71
	Aza	Ctrl	8.26	1.58	17.25	9.03	7.86
		VSV	248.36	293.34	335.04	292.25	43.35
IFN-β	Medium	Ctrl	5.96	1.85	14.95	7.59	6.70
		VSV	1420.36	1281.99	1241.36	1314.57	93.84
	Aza	Ctrl	8.98	14.75	19.02	14.25	5.04
		VSV	405.86	529.36	375.26	436.83	81.58
IFN-λ	Medium	Ctrl	2.57	8.26	14.57	8.47	6.00
		VSV	589.36	684.22	749.68	674.42	80.61
	Aza	Ctrl	6.04	19.28	2.11	9.14	9.00
		VSV	614.85	775.36	541.90	644.04	119.44
IL-6	Medium	Ctrl	5.39	6.91	11.24	7.85	3.04
		VSV	682.57	669.37	874.21	742.05	114.64
	Aza	Ctrl	8.95	12.35	23.04	14.78	7.35
		VSV	52.35	33.78	81.24	55.79	23.92
IL-1β	Medium	Ctrl	7.19	9.36	12.35	9.63	2.59
		VSV	578.36	682.24	654.78	638.46	53.83
	Aza	Ctrl	24.15	6.38	7.18	12.57	10.04
		VSV	34.28	41.75	86.32	54.12	28.14

Fig 6a

(i): The samples were separated on one gels, and the membrane was cut and incubated with the indicated antibodies.

(ii)-(iv): Because of similar molecular weight, another set of the same samples were separated on one gel, and the membrane was cut and incubated with the indicated antibodies.

Fig 6b

(i) ,(ii): Because of similar molecular weight, the same samples were separated on two gels, and the membranes was cut and incubated with the indicated antibodies.

(iii)-(v): Because of similar molecular weight, another set of the same samples were separated on two gels, and the membranes were cut and incubated with the indicated antibodies.

Fig 6c

(i)-(v):Because of similar molecular weight, the same samples were separated on two gels, and the membranes were cut and incubated with the indicated antibodies.

(vi)-(xi): Because of similar molecular weight, another set of the same samples were separated on three gels, and the membranes were cut and incubated with the indicated antibodies.

Fig 6a

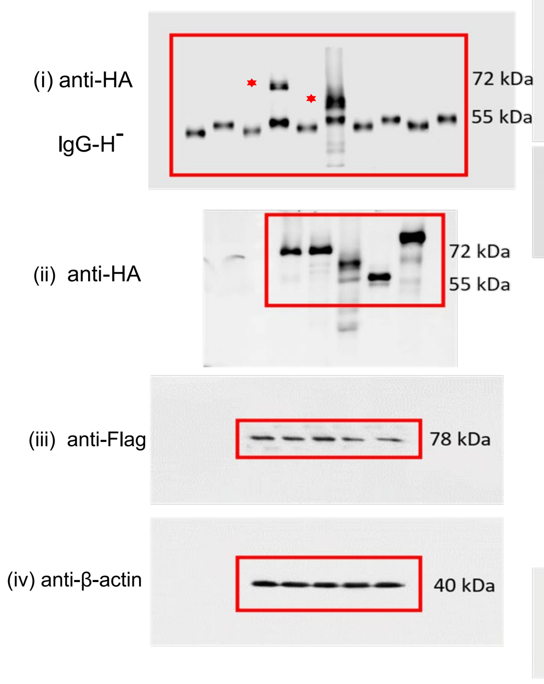

Fig 6b

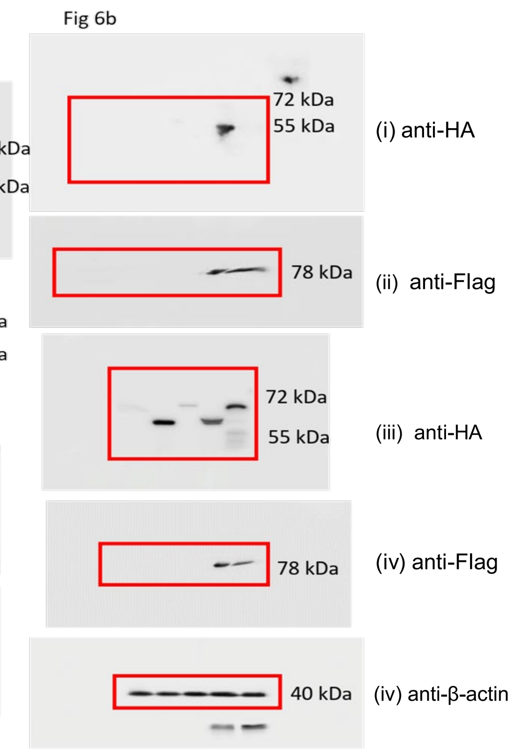

Fig 6c

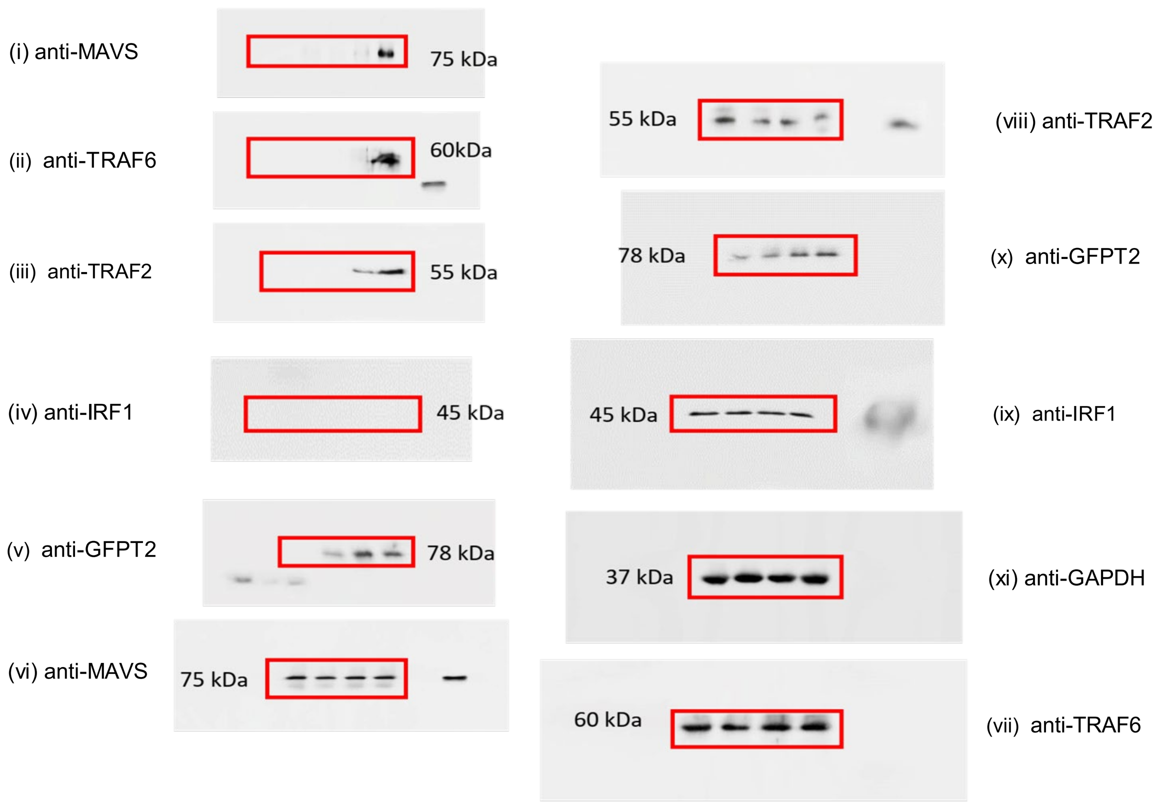

Fig 6d

(i)-(iv): The same samples were separated on two gels, and the membranes were cut and incubated with the indicated antibodies.

(v)-(ix): Another set of the same samples were separated on two gels, and the membranes were cut and incubated with the indicated antibodies.

Fig 6e

(i)-(iv):): The same samples were separated on two gels, and the membranes were cut and incubated with the indicated antibodies.

(v)-(ix): Another set of the same samples were separated on two gels, and the membranes were cut and incubated with the indicated antibodies.

Fig 6f

(i)-(iii): The same samples were separated on three gels, the membranes were cut and incubated with the indicated antibodies.

(iv)-(vi): Another set of the same samples were separated on two gels, and the membranes were cut and incubated with the indicated antibodies.

Fig 6g

(i), (ii): The same samples were separated on two gels, the membranes were cut and incubated with the indicated antibodies.

(iii)-(v): Because of similar molecular weight, another set of the same samples were separated on two gels, and the membranes were cut and incubated with the indicated antibodies.

Fig 6d

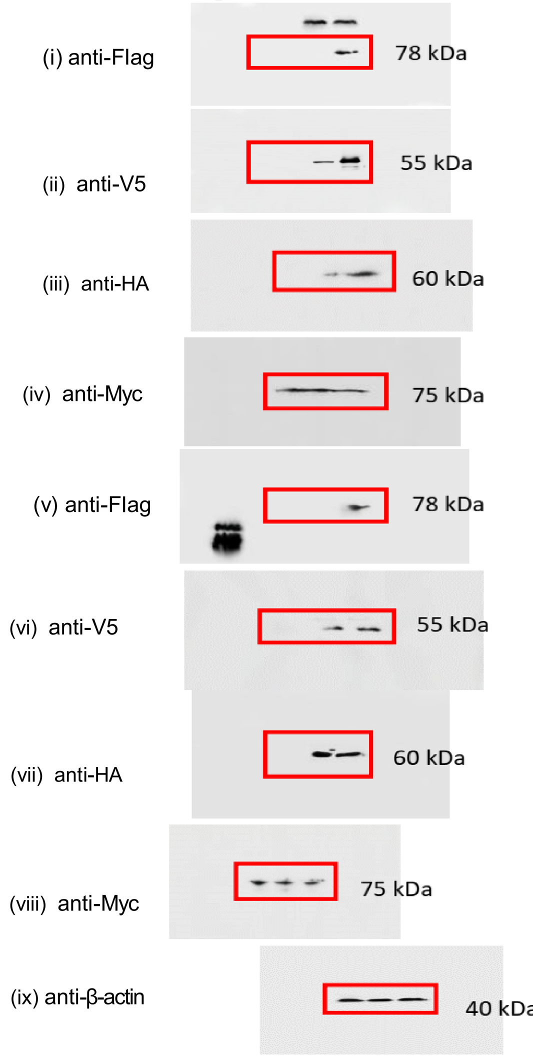

Fig 6e

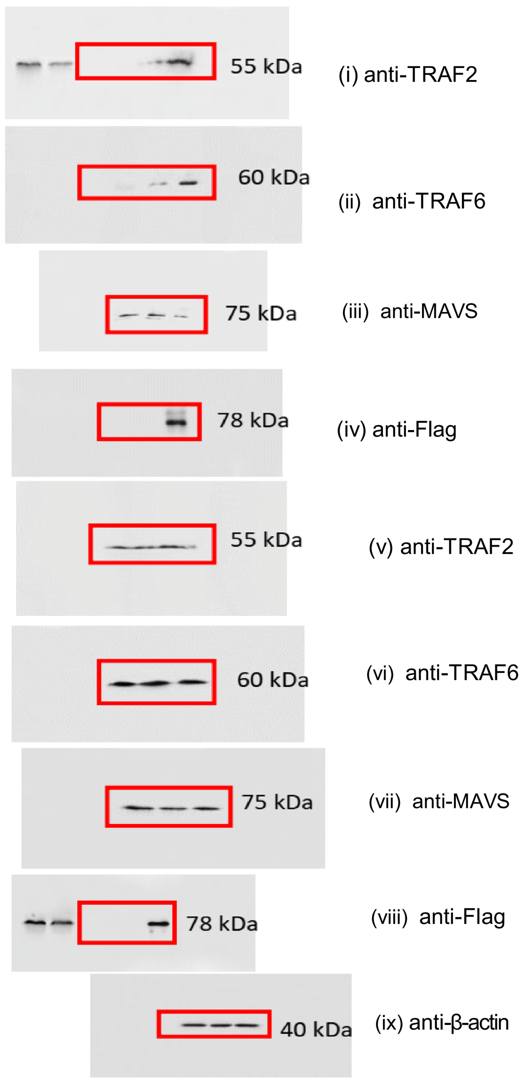

Fig 6f

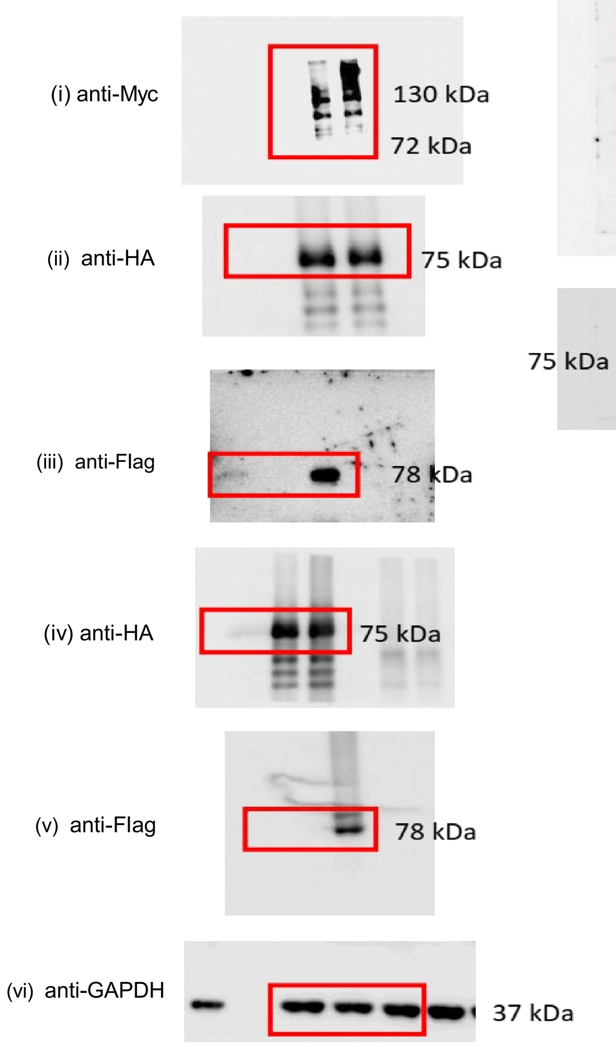

Fig 6g

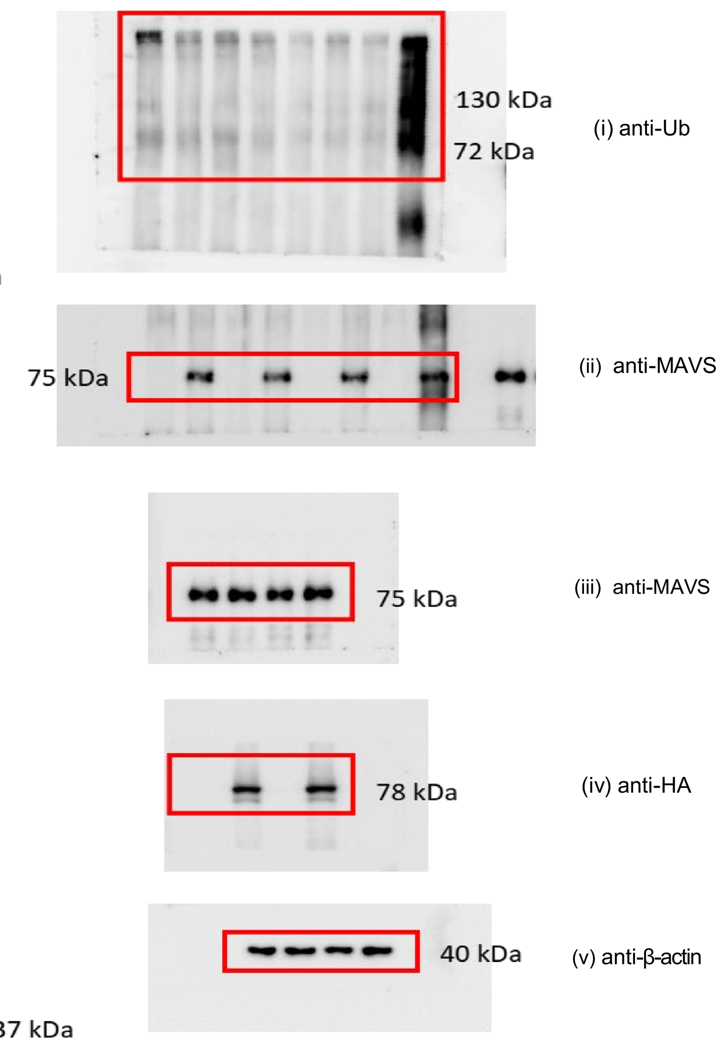

Fig 6h

(i) ,(ii): The same samples were separated on two gels, the membranes were cut and incubated with the indicated antibodies.

(iii)-(v): Another set of the same samples were separated on two gels, and the membranes were cut and incubated with the indicated antibodies.

Fig 6i

(i) ,(ii): The same samples were separated on two gels, the membranes were cut and incubated with the indicated antibodies.

(iii)-(v): Another set of the same samples were separated on two gels, and the membranes were cut and incubated with the indicated antibodies.

Fig 6h

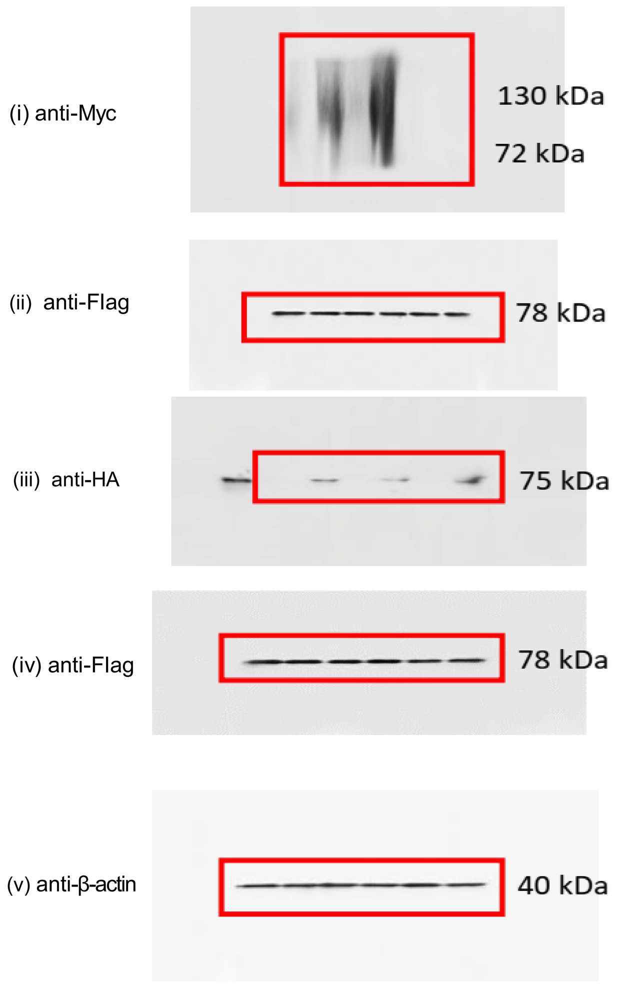

Fig 6i

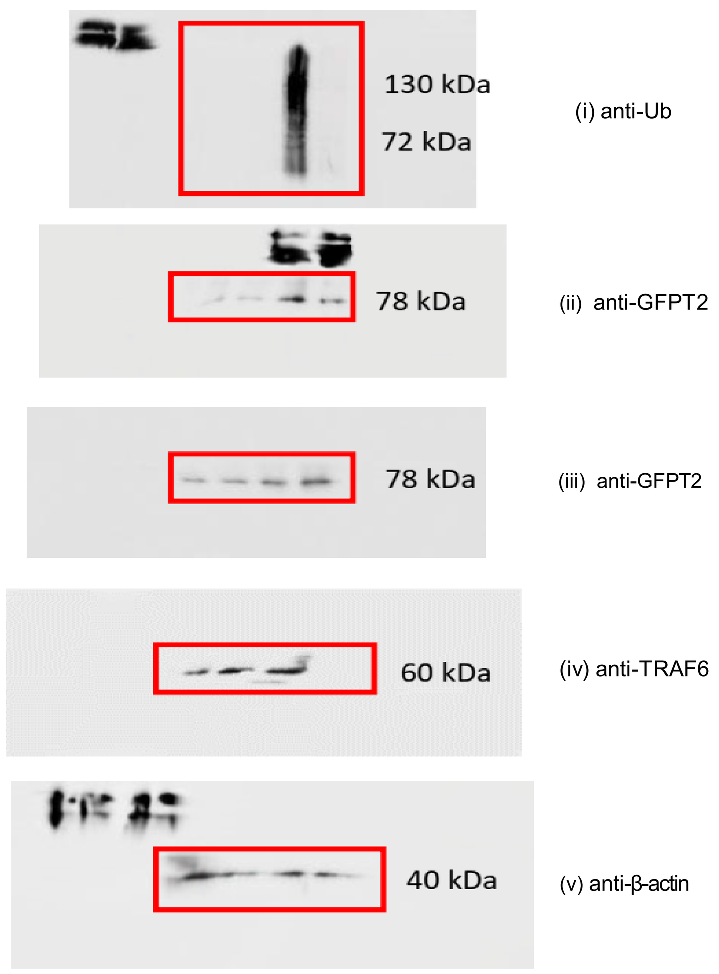

Fig 7a

Group ①: The samples were separated on two gels, and the membranes were cut and incubated with the indicated antibodies. (i) and (ii) are one set of same samples. (iii),(iv) and (v) are another set of same samples.

Group ②: The samples were separated on two gels, and the membranes were cut and incubated with the indicated antibodies. (i) and (ii) are one set of same samples. (iii),(iv) and (v) are another set of same samples.

Group③ : The samples were separated on two gels, and the membranes were cut and incubated with the indicated antibodies. (i) and (ii) are one set of same samples. (iii),(iv) and (v) are another set of same samples.

Group④ : The samples were separated on two gels, and the membranes were cut and incubated with the indicated antibodies. (i) and (ii) are one set of same samples. (iii),(iv) and (v) are another set of same samples.

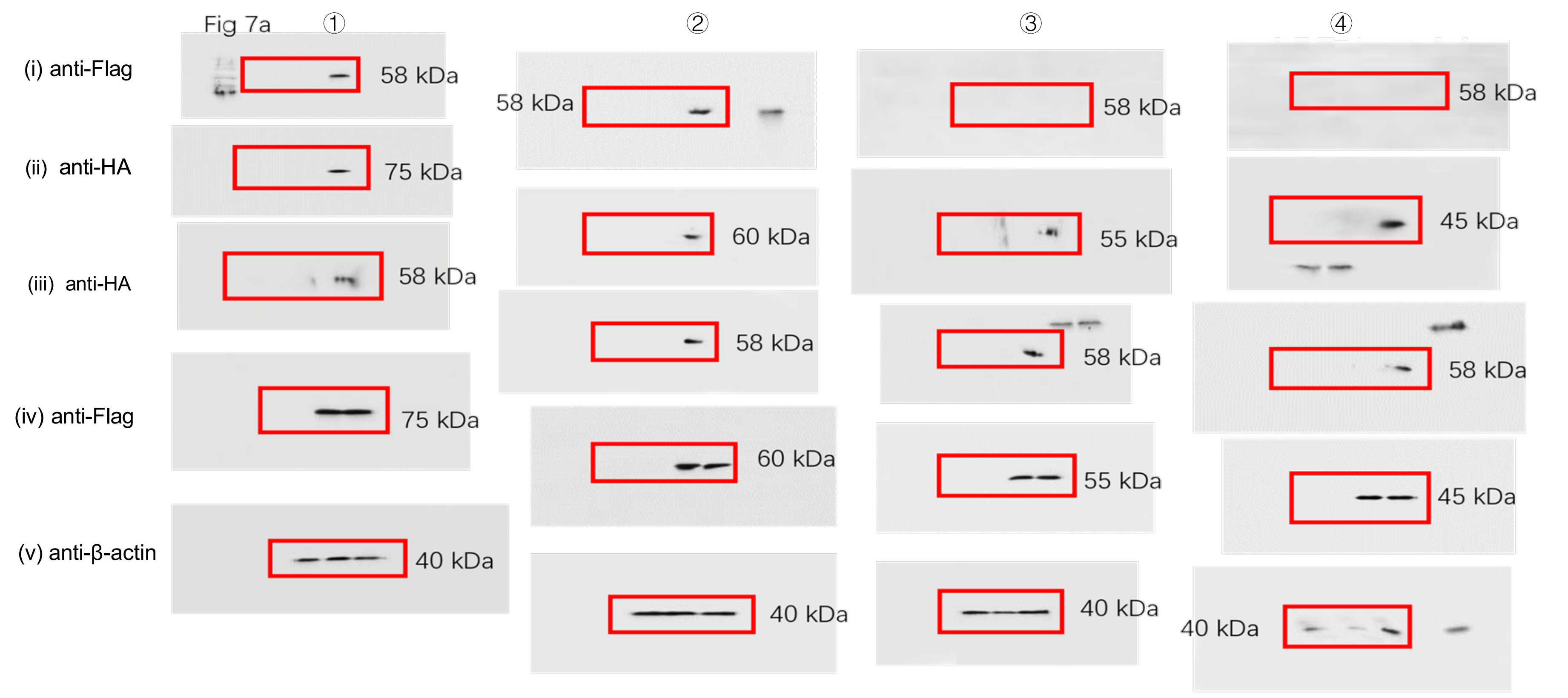

Fig 7b

(i)-(v): The same samples were separated on two gels, and the membranes were cut and incubated with the indicated antibodies.

(vi)-(xi): Another set of the same samples were separated on three gels, and the membrane were cut and incubated with the indicated antibodies.

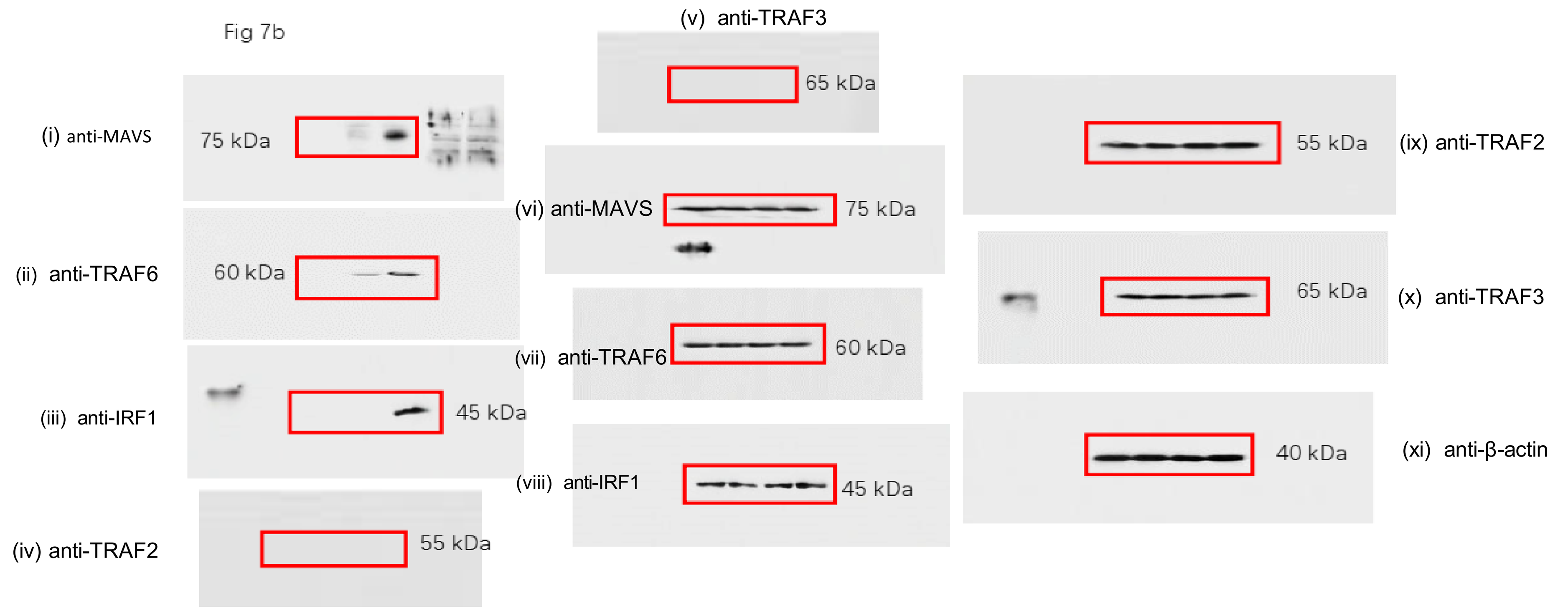

Fig 7c

(i)-(iv): The same samples were separated on three gels, and the membrane were cut and incubated with the indicated antibodies.

(v)-(ix): Another set of the same samples were separated on four gels, and the membranes were cut and incubated with the indicated antibodies.

Fig 7d

(i)-(iv): The same samples were separated on three gels, and the membrane were cut and incubated with the indicated antibodies.

(v)-(ix): Another set of the same samples were separated on three gels, and the membranes were cut and incubated with the indicated antibodies.

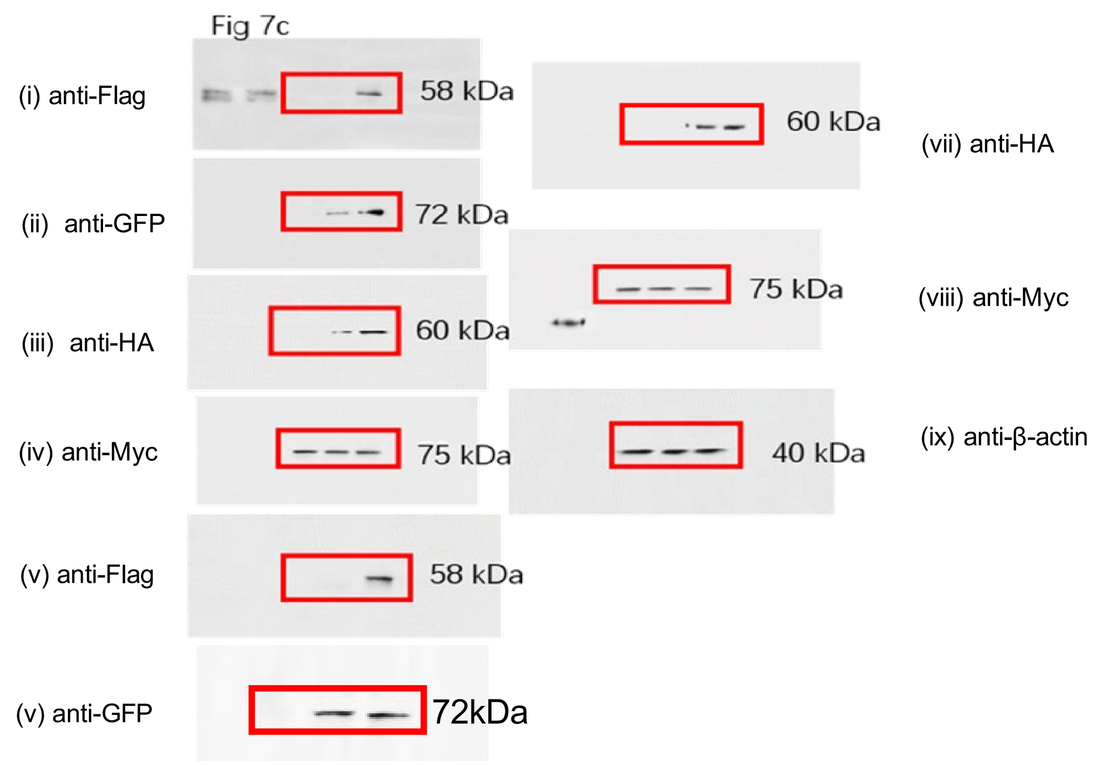

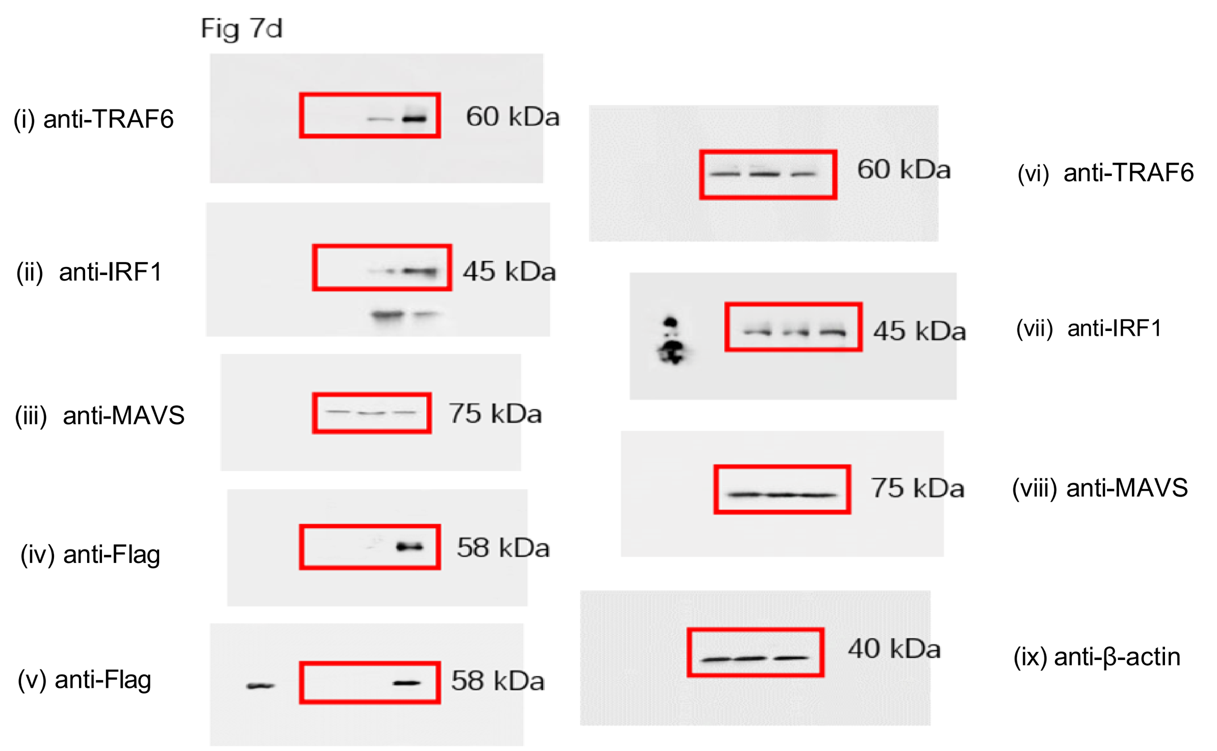

Fig 7e

(i)-(ii): The same samples were separated on two gels, and the membranes were cut and incubated with the indicated antibodies.

(iii)-(v): Another set of the same samples were separated on three gels, and the membranes were cut and incubated with the indicated antibodies.

Fig 7f

(i)-(ii): The same samples were separated on two gels, and the membranes were cut and incubated with the indicated antibodies.

(iii)-(vi):): Another set of the same samples were separated on three gels, and the membranes were cut and incubated with the indicated antibodies

Fig 7g

(i)-(iv): The same samples were separated on two gels, and the membranes were cut and incubated with the indicated antibodies.

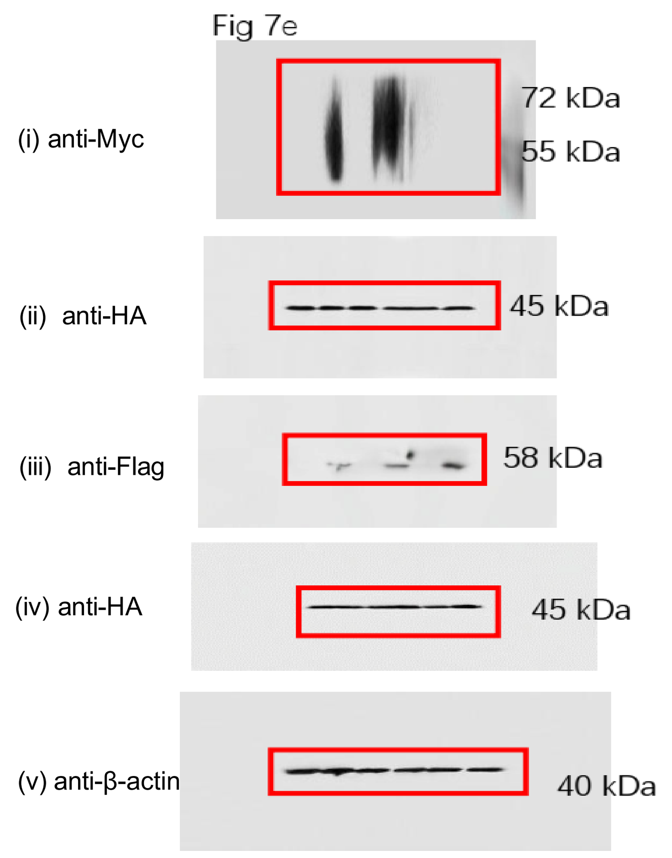

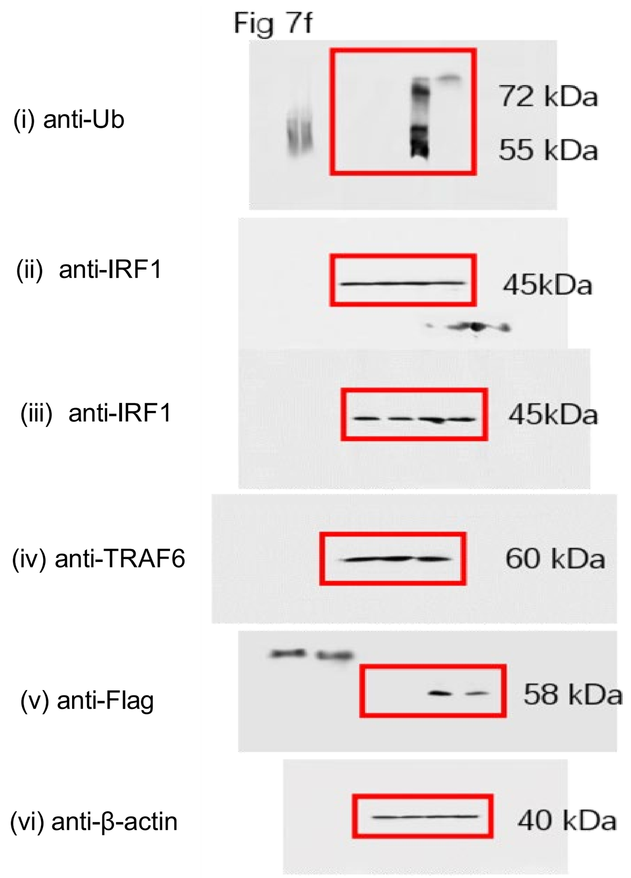

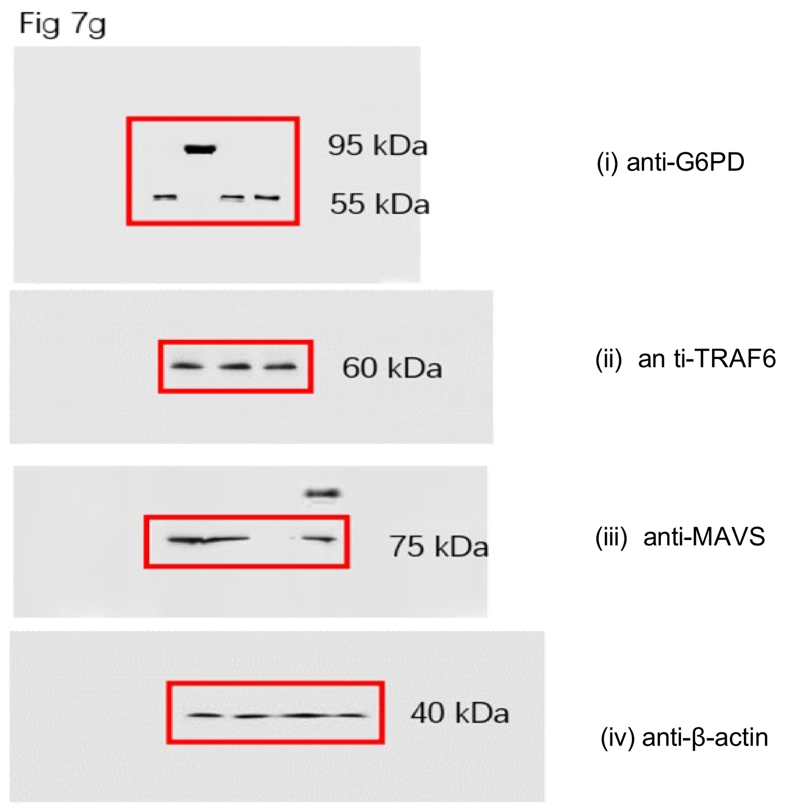

Fig 8a
(i)-(vii): The same samples were separated on three gels, and the membranes were cut and incubated with the indicated antibodies.
(viii)-(xv): Another set of the same samples were separated on four gel, and the membranes were cut and incubated with he indicated antibodies.

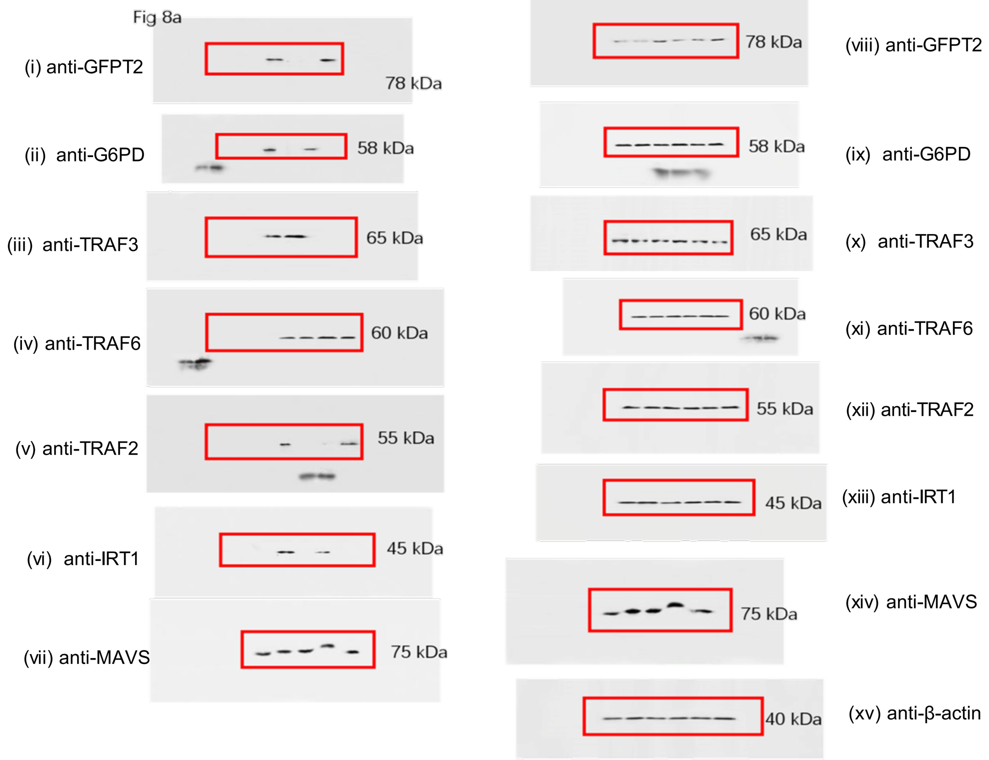

Fig 8b
(i): The same samples were separated on one gel, and the membranes were cut and incubated with the indicated antibodies.
(ii)-(iii): The same samples were separated one gel, and the membrane was cut and incubated with the indicated antibodies.

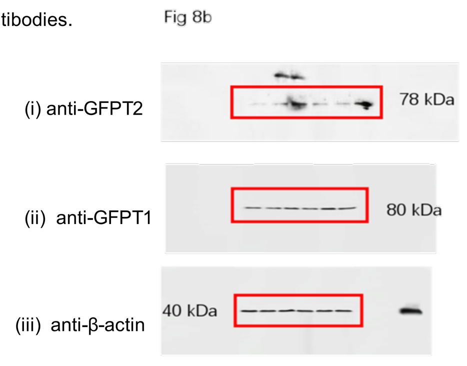

Fig 8c
(i)-(ii): The same samples were separated on two gel, and the membranes were cut and incubated with the indicated antibodies.
(iii)-(v): The same samples were separated two gel, and the membrane were cut and incubated with the indicated antibodies.
Fig 8e
(i)-(ii): The same samples were separated one gel, and the membrane was cut and incubated with the indicated antibodies.

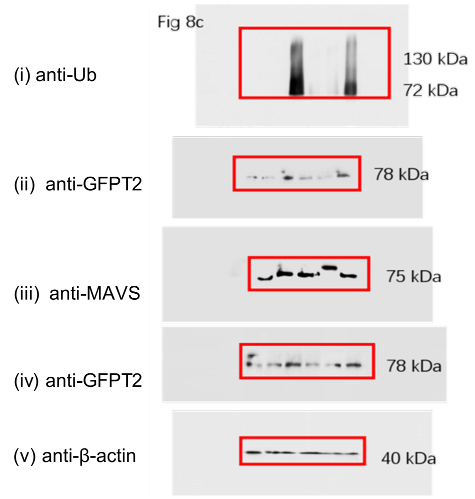

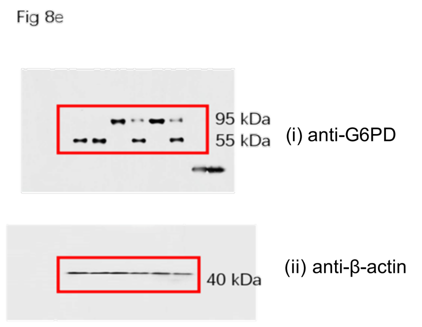

Fig 8f group1-4
Group 1 Mitochondria
(i)-(xi) :The same samples were separated five gel, and the membrane was cut and incubated with the indicated antibodies.

Group 2 MAM
(i)-(xi) :The same samples were separated five gel, and the membrane was cut and incubated with the indicated antibodies.

Group 3 Peroxisome
(i)-(xi) :The same samples were separated five gel, and the membrane was cut and incubated with the indicated antibodies.
Group 4 Cytosol
(i)-(xi) :The same samples were separated five gel, and the membrane was cut and incubated with the indicated antibodies.

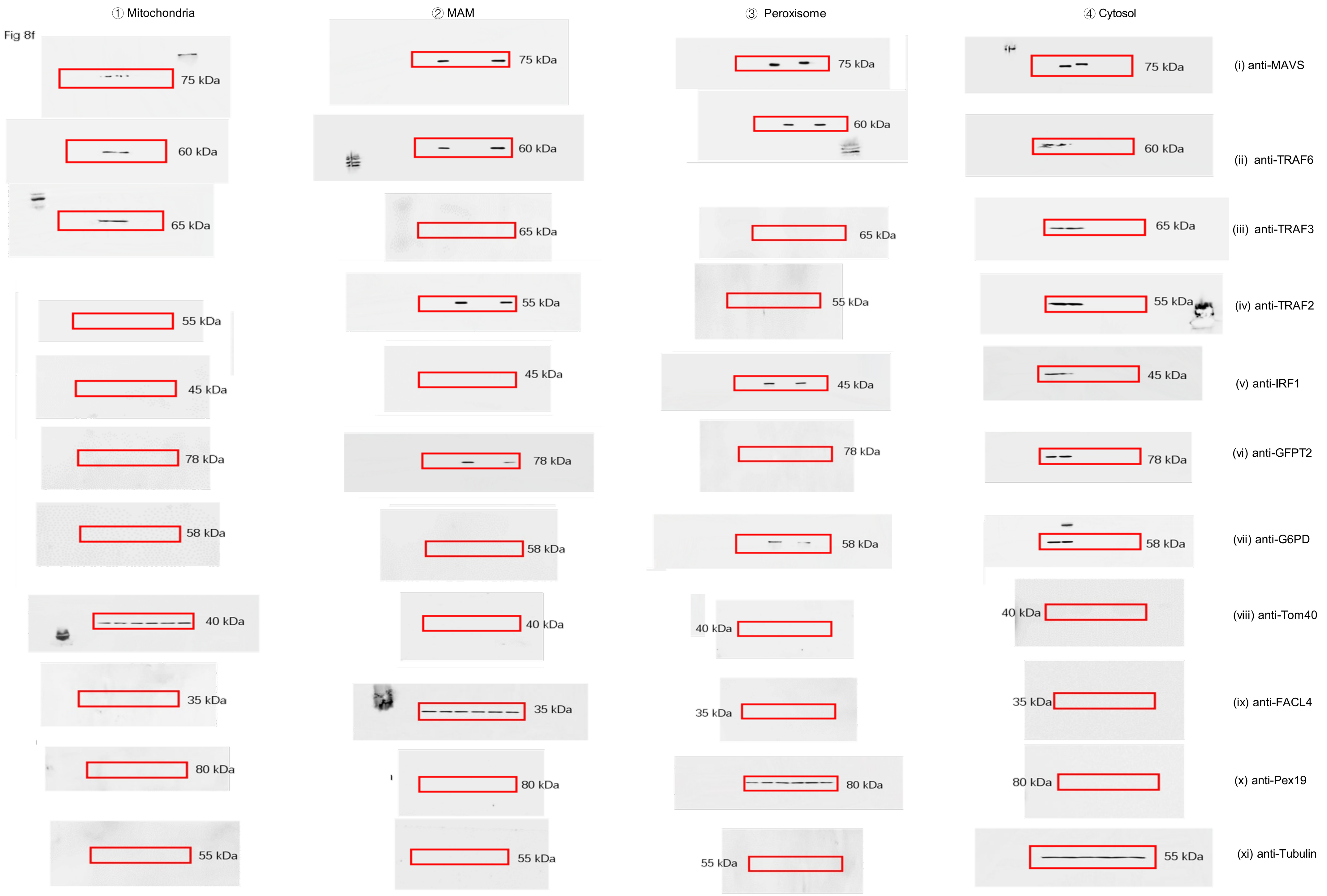

Fig8d: G6PD activity					
	1	2	3	AVE	STDEV
ctrl	1.00	1.02	1.17	1.06	0.09
cyto	0.86	1.15	1.07	1.03	0.15
wt	1.73	1.63	1.89	1.75	0.13
mito	1.02	0.93	1.14	1.03	0.11
pex	2.01	2.14	2.11	2.09	0.07
mam	0.96	1.15	1.06	1.06	0.10

Fig8g: NF-κB luciferase activity						
		1	2	3	AVE	STDEV
sh-Ctrl	ctrl	1.00	2.35	3.04	2.13	1.04
	cyto	2.05	1.96	1.84	1.95	0.11
	wt	31.85	37.05	43.25	37.38	5.71
	mito	12.75	17.96	15.24	15.32	2.61
	pex	43.55	47.01	40.15	43.57	3.43
	mam	38.25	44.84	42.05	41.71	3.31
sh-GFPT2	ctrl	1.52	3.05	2.44	2.34	0.77
	cyto	1.04	1.52	3.77	2.11	1.46
	wt	6.75	4.18	9.24	6.72	2.53
	mito	18.26	21.24	16.33	18.61	2.47
	pex	41.27	44.95	49.22	45.15	3.98
	mam	3.25	5.44	1.17	3.29	2.14
sh-G6PD	ctrl	1.08	1.17	2.33	1.53	0.70
	cyto	2.51	1.93	1.72	2.05	0.41
	wt	12.39	7.47	18.92	12.93	5.74
	mito	14.62	17.09	16.22	15.98	1.25
	pex	13.91	15.66	21.12	16.90	3.76
	mam	43.05	40.11	35.29	39.48	3.92

Fig8h: ISRE luciferase activity						
		1	2	3	AVE	STDEV
sh-Ctrl	ctrl	1.00	2.63	1.27	1.63	0.87
	cyto	0.86	3.04	2.11	2.00	1.09
	wt	14.27	17.88	20.41	17.52	3.09
	mito	9.38	10.25	8.36	9.33	0.95
	pex	19.35	21.25	24.62	21.74	2.67
	mam	30.44	34.26	37.28	33.99	3.43
sh-GFPT2	ctrl	1.52	2.33	3.06	2.30	0.77
	cyto	2.14	3.96	1.05	2.38	1.47
	wt	2.36	4.25	6.38	4.33	2.01
	mito	12.42	11.05	9.36	10.94	1.53
	pex	23.55	26.96	21.08	23.86	2.95
	mam	6.35	4.05	8.92	6.44	2.44
sh-G6PD	ctrl	1.55	1.96	2.37	1.96	0.41
	cyto	1.05	2.64	3.14	2.28	1.09
	wt	9.96	13.78	8.88	10.87	2.57
	mito	9.17	13.96	9.92	11.02	2.58
	pex	12.36	9.85	21.05	14.42	5.88
	mam	32.99	36.18	34.11	34.43	1.62

Fig8i: IRF1 luciferase activity						
		1	2	3	AVE	STDEV
sh-Ctrl	ctrl	1.00	2.99	1.08	1.69	1.13
	cyto	2.14	3.05	1.47	2.22	0.79
	wt	23.24	26.95	28.19	26.13	2.58
	mito	8.29	11.24	7.86	9.13	1.84
	pex	12.75	17.95	18.33	16.34	3.12
	mam	5.86	4.18	7.25	5.76	1.54
sh-GFPT2	ctrl	1.24	1.05	2.04	1.44	0.53
	cyto	2.06	1.78	1.96	1.93	0.14
	wt	21.35	24.85	29.33	25.18	4.00
	mito	6.35	14.25	12.76	11.12	4.20
	pex	19.08	14.75	16.22	16.68	2.20
	mam	6.35	2.17	10.42	6.31	4.13
sh-G6PD	ctrl	1.84	2.33	1.09	1.75	0.62
	cyto	0.96	1.75	0.86	1.19	0.49
	wt	5.24	4.18	3.36	4.26	0.94
	mito	10.24	9.14	6.05	8.48	2.17
	pex	2.06	1.47	3.26	2.26	0.91
	mam	4.15	2.69	9.22	5.35	3.43

Fig8j: ISG56 mRNA level						
		1	2	3	AVE	STDEV
sh-Ctrl	ctrl	1.00	1.63	2.25	1.63	0.63
	cyto	1.33	1.96	3.52	2.27	1.13
	wt	34.28	36.91	29.33	33.51	3.85
	mito	12.85	10.96	15.26	13.02	2.16
	pex	38.29	34.11	39.05	37.15	2.66
	mam	14.27	10.95	9.38	11.53	2.50
sh-GFPT2	ctrl	1.05	1.19	2.39	1.54	0.74
	cyto	2.75	3.67	4.18	3.53	0.72
	wt	6.35	4.18	7.96	6.16	1.90
	mito	17.26	12.06	9.36	12.89	4.02
	pex	42.05	37.19	36.09	38.44	3.17
	mam	2.74	3.06	4.11	3.30	0.72
sh-G6PD	ctrl	1.17	1.52	1.65	1.45	0.25
	cyto	2.05	3.14	1.82	2.34	0.71
	wt	12.75	16.39	18.09	15.74	2.73
	mito	11.95	14.59	17.05	14.53	2.55
	pex	4.89	6.91	8.17	6.66	1.65
	mam	12.07	8.99	15.37	12.14	3.19

Fig8k: ISG15 mRNA level						
		1	2	3	AVE	STDEV
sh-Ctrl	ctrl	1.00	1.53	1.61	1.38	0.33
	cyto	1.59	2.33	1.91	1.94	0.37
	wt	24.81	26.95	29.33	27.03	2.26
	mito	14.85	16.09	17.18	16.04	1.17
	pex	26.38	29.14	22.35	25.96	3.41
	mam	16.24	12.68	19.25	16.06	3.29
sh-GFPT2	ctrl	2.54	1.82	3.04	2.47	0.61
	cyto	1.08	1.56	2.73	1.79	0.85
	wt	4.57	3.96	2.33	3.62	1.16
	mito	19.33	15.14	11.57	15.35	3.88
	pex	24.84	27.14	23.04	25.01	2.06
	mam	2.51	3.05	2.77	2.78	0.27
sh-G6PD	ctrl	1.71	1.55	3.18	2.15	0.90
	cyto	3.05	1.27	1.63	1.98	0.94
	wt	9.36	10.27	7.81	9.15	1.24
	mito	18.74	16.24	13.99	16.32	2.38
	pex	4.81	5.96	2.19	4.32	1.93
	mam	14.24	17.26	13.92	15.14	1.84

Fig s1a-b

(i)-(ii) :The samples were separated one gel, and the membrane was cut and incubated with the indicated antibodies.

Fig s1a

Fig s1b

(i) anti-MAVS

(i) anti-β-actin

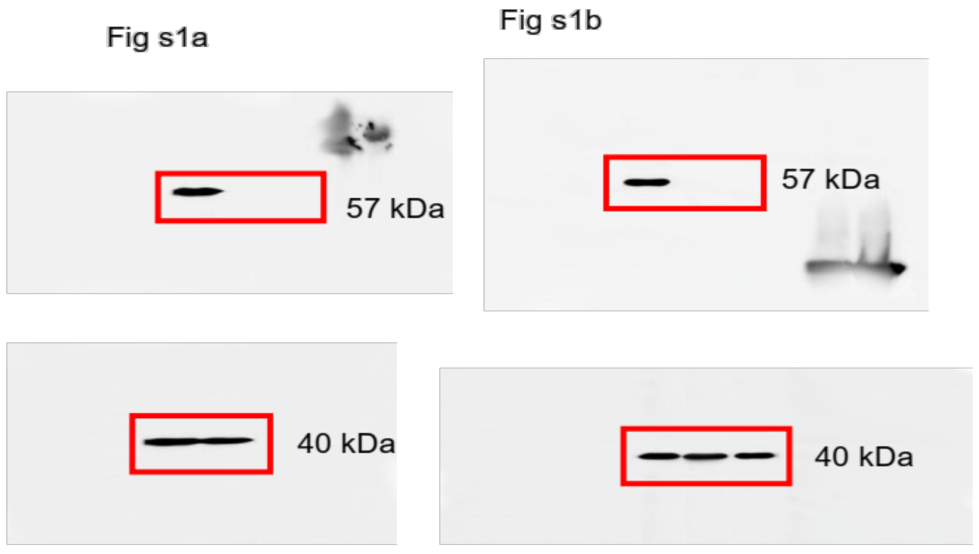

Fig S1-c: glucose levels						
		1	2	3	AVE	STDEV
wt	Ctrl	4.08	4.36	4.22	4.22	0.14
	VSV	11.71	10.92	12.29	11.64	0.69
mamv-/-	Ctrl	3.25	4.12	3.82	3.73	0.44
	VSV	3.62	3.76	3.89	3.76	0.14

Fig S1-d: glucose levels						
		1	2	3	AVE	STDEV
wt	Ctrl	2.24	2.89	3.06	2.73	0.43
	Poly(I:C)	6.37	7.19	7.81	7.12	0.72
mamv-/-	Ctrl	3.14	2.94	3.55	3.21	0.31
	Poly(I:C)	3.09	3.48	2.61	3.06	0.44
		1	2	3	AVE	STDEV
wt	Ctrl	2.88	3.51	3.29	3.23	0.32
	SeV	7.09	9.11	8.17	8.12	1.01
mamv-/-	Ctrl	2.67	3.05	3.44	3.05	0.39
	SeV	2.04	2.89	3.62	2.85	0.79

Fig S1-h: upper glycolytic metabolism												
			M0					M2				
			1	2	3	AVE	STDEV	1	2	3	AVE	STDEV
G6P	wt	Ctrl	61.56	65.18	54.28	60.34	5.55	40.85	45.19	36.29	40.78	4.45
		VSV	20.71	18.09	27.83	22.21	5.04	75.26	78.14	83.06	78.82	3.94
	mamv-/-	Ctrl	60.88	57.18	63.77	60.61	3.30	37.18	44.07	40.26	40.50	3.45
		VSV	62.05	63.27	54.09	59.80	4.99	37.33	42.17	43.96	41.15	3.43
F1,6P	wt	Ctrl	22.35	30.15	16.04	22.85	7.07	72.16	74.15	77.26	74.52	2.57
		VSV	72.15	65.24	61.07	66.15	5.60	31.25	27.18	36.95	31.79	4.91
	mamv-/-	Ctrl	27.18	25.17	20.99	24.45	3.16	74.18	79.26	70.18	74.54	4.55
		VSV	23.08	27.92	20.33	23.78	3.84	70.88	73.26	76.15	73.43	2.64
F6P	wt	Ctrl	55.24	57.16	50.21	54.20	3.59	48.26	50.26	45.26	47.93	2.52
		VSV	26.33	30.17	20.85	25.78	4.68	82.04	78.29	74.28	78.20	3.88
	mamv-/-	Ctrl	45.14	57.02	60.24	54.13	7.95	46.04	49.25	51.27	48.85	2.64
		VSV	61.24	53.29	57.18	57.24	3.98	48.19	44.04	41.20	44.48	3.52

Fig S1-i: PPP metabolism																		
			M0					M1					M2					
			1	2	3	AVE	STDEV	1	2	3	AVE	STDEV	1	2	3	AVE	STDEV
GAP	wt	Ctrl	33.39	48.69	49.33	43.80	9.02	3.26	5.96	9.26	6.16	3.00	45.26	41.71	52.91	46.63	5.72
		VSV	18.63	13.69	10.27	14.20	4.20	15.24	11.08	17.39	14.57	3.21	68.17	72.09	63.29	67.85	4.41
	mamv-/-	Ctrl	39.60	47.29	51.36	46.08	5.97	4.18	6.39	7.19	5.92	1.56	41.28	45.96	49.36	45.53	4.06
		VSV	42.03	51.26	44.09	45.79	4.85	1.39	7.29	8.06	5.58	3.65	38.99	46.35	48.16	44.50	4.86
ATP	wt	Ctrl	77.26	87.29	89.33	84.63	6.46	8.09	15.67	17.17	13.64	4.87	5.36	6.38	13.22	8.32	4.27
		VSV	60.25	58.77	71.93	63.65	7.21	21.05	23.85	24.19	23.03	1.72	15.29	17.26	18.62	17.06	1.67
	mamv-/-	Ctrl	75.26	84.96	98.37	86.20	11.60	9.36	13.27	16.77	13.13	3.71	4.15	3.96	10.26	6.12	3.58
		VSV	87.63	87.92	87.34	87.63	0.29	10.85	13.78	16.39	13.67	2.77	3.29	4.96	9.97	6.07	3.48
R5P	wt	Ctrl	78.25	71.44	76.39	75.36	3.52	12.06	10.17	17.95	13.39	4.06	12.36	17.92	16.33	15.54	2.86
		VSV	32.26	44.15	43.06	39.82	6.57	28.19	36.24	38.04	34.16	5.25	35.26	25.69	33.14	31.36	5.03
	mamv-/-	Ctrl	63.26	75.29	78.19	72.25	7.92	12.11	20.78	14.26	15.72	4.51	14.81	18.29	20.36	17.82	2.80
		VSV	72.02	77.95	80.36	76.78	4.29	14.27	9.35	17.19	13.60	3.96	10.28	16.39	19.36	15.34	4.63

Fig S1-j: TCA cycle metabolism																		
			M0					M1					M2					
			1	2	3	AVE	STDEV	1	2	3	AVE	STDEV	1	2	3	AVE	STDEV
Pyruvate	wt	Ctrl	19.14	13.04	10.69	14.29	4.36	17.26	8.26	6.21	10.58	5.88	78.26	75.11	70.26	74.54	4.03
		VSV	45.26	60.74	47.19	51.06	8.44	27.29	17.96	21.06	22.10	4.75	32.08	35.29	27.01	31.46	4.17
	mamv-/-	Ctrl	11.71	14.51	19.36	15.19	3.87	12.36	10.74	9.19	10.76	1.59	73.34	70.29	77.91	73.85	3.84
		VSV	16.35	18.97	7.33	14.22	6.11	11.74	8.05	14.67	11.49	3.32	68.19	77.18	80.26	75.21	6.27
Lactate	wt	Ctrl	28.26	32.28	26.11	28.88	3.13	16.32	22.69	17.33	18.78	3.42	45.26	47.29	40.81	44.45	3.31
		VSV	43.29	54.18	40.39	45.95	7.27	27.29	31.05	33.72	30.69	3.23	18.99	23.74	16.05	19.59	3.88
	mamv-/-	Ctrl	26.38	33.07	34.84	31.43	4.46	18.39	23.04	16.04	19.16	3.56	43.05	40.18	44.97	42.73	2.41
		VSV	31.25	27.26	36.17	31.56	4.46	20.36	22.36	17.36	20.03	2.52	38.29	45.26	42.74	42.10	3.53
Fumarate	wt	Ctrl	72.25	68.36	64.17	68.26	4.04	13.69	17.98	11.04	14.24	3.50	15.69	19.36	14.21	16.42	2.65
		VSV	71.25	77.22	66.33	71.60	5.45	22.36	25.96	23.33	23.88	1.86	4.58	2.69	5.63	4.30	1.49
	mamv-/-	Ctrl	69.25	62.36	64.04	65.22	3.59	15.96	17.95	19.36	17.76	1.71	16.38	17.15	18.63	17.39	1.14
		VSV	70.18	68.68	76.74	71.87	4.29	11.85	16.33	12.04	13.41	2.53	14.26	11.28	19.96	15.17	4.41

S2-b: LC-MS GlcN-6P level												
			M1					M0				
			1	2	3	AVE	STDEV	1	2	3	AVE	STDEV
GlcN-6P	wt	Ctrl	53.26	62.38	67.11	60.92	7.04	38.26	41.33	45.09	41.56	3.42
		VSV	81.27	83.26	88.16	84.23	3.55	16.35	22.06	17.33	18.58	3.05
	mamv-/-	Ctrl	61.87	64.25	50.96	59.03	7.09	42.36	34.15	49.96	42.16	7.91
		VSV	65.74	68.26	60.14	64.71	4.16	40.55	34.25	37.95	37.58	3.17

S2-c: LC-MS GlcNAc-6P level												
			M1					M0				
			1	2	3	AVE	STDEV	1	2	3	AVE	STDEV
GlcNAc-6P	wt	Ctrl	42.65	57.36	52.11	50.71	7.45	47.36	50.21	45.24	47.60	2.49
		VSV	81.26	88.36	73.26	80.96	7.55	21.36	18.24	16.71	18.77	2.37
	mamv-/-	Ctrl	40.26	53.24	57.26	50.25	8.88	48.36	54.26	47.25	49.96	3.77
		VSV	44.26	53.96	47.25	48.49	4.97	52.93	58.24	42.26	51.14	8.14

S2-d: LC-MS UDP-GlcN level												
			M1					M0				
			1	2	3	AVE	STDEV	1	2	3	AVE	STDEV
UDP-GlcNAc	wt	Ctrl	38.26	43.28	47.26	42.93	4.51	63.29	55.95	68.15	62.46	6.14
		VSV	73.96	82.55	73.95	76.82	4.96	28.69	33.71	24.69	29.03	4.52
	mamv-/-	Ctrl	41.74	43.96	37.26	40.99	3.41	64.25	66.17	57.93	62.78	4.31
		VSV	43.09	37.26	47.29	42.55	5.04	61.05	68.99	56.35	62.13	6.39

Fig S2-e: LC-MS ManNAc level												
			M1					M0				
			1	2	3	AVE	STDEV	1	2	3	AVE	STDEV
ManNAc	wt	Ctrl	32.69	27.14	36.09	31.97	4.52	71.26	79.36	62.08	70.90	8.65
		VSV	58.60	61.36	63.24	61.07	2.33	47.26	31.02	49.33	42.54	10.03
	mamv-/-	Ctrl	30.74	35.17	24.91	30.27	5.15	79.36	73.37	67.24	73.32	6.06
		VSV	32.69	27.25	36.14	32.03	4.48	76.36	77.25	62.37	71.99	8.35

Fig S2-f: LC-MS Neu5Ac level												
			M1					M0				
			1	2	3	AVE	STDEV	1	2	3	AVE	STDEV
Neu5Ac	wt	Ctrl	8.36	12.36	14.17	11.63	2.97	83.04	90.25	94.71	89.33	5.89
		VSV	18.09	22.36	24.95	21.80	3.46	75.14	84.36	78.91	79.47	4.64
	mamv-/-	Ctrl	4.26	14.26	9.35	9.29	5.00	97.26	93.24	85.27	91.92	6.10
		VSV	6.34	16.35	12.07	11.59	5.02	88.09	96.35	85.24	89.89	5.77

Fig S2-g: metabolites quantified by metabolomics						
		1	2	3	AVE	STDEV
G6P	ctrl	1.03	0.86	0.47	0.79	0.29
	polyic	2.15	1.89	3.36	2.47	0.78
	polyic+sh-mavs	0.89	1.14	0.45	0.83	0.35
F6P	ctrl	1.00	1.36	0.57	0.98	0.40
	polyic	3.26	2.39	2.06	2.57	0.62
	polyic+sh-mavs	0.86	0.96	1.35	1.06	0.26

Fig S2-h: metabolites quantified by metabolomics						
		1	2	3	AVE	STDEV
F1,6P	ctrl	1.00	0.86	1.33	1.06	0.24
	polyic	0.53	0.44	0.36	0.44	0.09
	polyic+sh-mavs	1.36	0.86	0.77	1.00	0.32
3PG	ctrl	1.00	1.23	1.06	1.10	0.12
	polyic	0.62	0.48	0.33	0.48	0.15
	polyic+sh-mavs	1.17	0.71	0.69	0.86	0.27
Pyruvate	ctrl	1.00	0.86	0.78	0.88	0.11
	polyic	0.47	0.66	0.34	0.49	0.16
	polyic+sh-mavs	1.05	1.14	0.76	0.98	0.20
Lactate	ctrl	1.00	1.03	0.91	0.98	0.06
	polyic	0.24	0.32	0.17	0.24	0.08
	polyic+sh-mavs	1.14	0.86	0.77	0.92	0.19
Succinate	ctrl	1.00	0.77	0.69	0.82	0.16
	polyic	0.26	0.38	0.14	0.26	0.12
	polyic+sh-mavs	1.06	0.85	1.27	1.06	0.21

Fig S2-i: metabolites quantified by metabolomics						
		1	2	3	AVE	STDEV
6PG	ctrl	1.00	1.26	1.36	1.21	0.19
	polyic	2.96	2.47	2.06	2.50	0.45
	olyic+sh-mav	1.05	1.35	0.87	1.09	0.24
R5P	ctrl	1.00	0.86	1.35	1.07	0.25
	polyic	3.26	2.89	3.75	3.30	0.43
	olyic+sh-mav	0.86	1.41	0.52	0.93	0.45
S7P	ctrl	1.00	1.25	1.41	1.22	0.21
	polyic	2.47	2.55	2.63	2.55	0.08
	olyic+sh-mav	0.88	1.05	1.14	1.02	0.13
GSH	ctrl	1.00	1.27	0.78	1.02	0.25
	polyic	2.04	2.74	2.51	2.43	0.36
	olyic+sh-mav	1.24	0.86	0.91	1.00	0.21

Fig S2-j: metabolites quantified by metabolomics						
		1	2	3	AVE	STDEV
GlcN-6P	ctrl	1.00	1.24	0.86	1.03	0.19
	polyic	2.47	2.28	2.96	2.57	0.35
	olyic+sh-mav	1.20	1.08	1.31	1.20	0.12
GlcNAc-6P	ctrl	1.00	0.84	1.39	1.08	0.28
	polyic	2.34	2.08	2.74	2.39	0.33
	olyic+sh-mav	0.86	0.92	1.17	0.98	0.16
GlcNAc-1P	ctrl	1.00	1.47	0.86	1.11	0.32
	polyic	3.11	3.05	3.47	3.21	0.23
	olyic+sh-mav	0.76	0.87	1.15	0.93	0.20
UDP-GlcNAc	ctrl	1.00	0.86	1.07	0.98	0.11
	polyic	2.96	3.69	3.77	3.47	0.45
	olyic+sh-mav	1.14	0.89	1.23	1.09	0.18

Fig S3-a: mRNA level						
		1	2	3	AVE	STDEV
Glut1	ctrl	1.00	1.25	0.86	1.04	0.20
	polyic	5.86	7.29	9.33	7.49	1.74
	polyic+sh-mavs	2.34	3.15	1.24	2.24	0.96
Glut 4	ctrl	1.00	1.33	1.17	1.17	0.17
	polyic	13.74	10.36	16.79	13.63	3.22
	polyic+sh-mavs	3.24	4.96	2.94	3.71	1.09

Fig S3-b: HK activity					
	1	2	3	AVE	STDEV
Ctrl	1.00	0.92	0.93	0.95	0.04
MAVS	1.67	1.73	1.52	1.64	0.11
sh-Ctrl	1.00	1.14	0.93	1.02	0.11
sh-MAVS	0.51	0.43	0.57	0.50	0.07

Fig S3-c: Pyruvate level					
	1	2	3	AVE	STDEV
ctrl	1.00	0.94	1.24	1.06	0.16
polyic	0.52	0.44	0.58	0.51	0.07
polyic+sh-mavs	1.14	1.05	0.89	1.03	0.13

Fig S3-d: Lactate level					
	1	2	3	AVE	STDEV
ctrl	1.00	0.95	1.14	1.03	0.10
polyic	0.43	0.38	0.33	0.38	0.05
polyic+sh-mavs	0.89	0.92	0.99	0.93	0.05

Fig S3-e: Succinate level					
	1	2	3	AVE	STDEV
ctrl	1.00	0.93	1.14	1.02	0.11
polyic	0.63	0.66	0.71	0.67	0.04
polyic+sh-mavs	0.95	0.89	0.93	0.92	0.03

Fig S3-f: G6PD activity					
	1	2	3	AVE	STDEV
ctrl	1.00	0.86	0.88	0.91	0.08
polyic	1.67	1.88	1.52	1.69	0.18
polyic+sh-mavs	1.14	0.93	0.86	0.98	0.15

Fig S3-h: NADPH level					
	1	2	3	AVE	STDEV
ctrl	1.00	0.89	1.23	1.06	0.17
polyic	0.51	0.58	0.67	0.63	0.08
polyic+sh-mavs	0.91	1.17	1.05	1.11	0.13

Fig S3-g: NADP+/NADPH					
	1	2	3	AVE	STDEV
ctrl	1.00	0.96	1.16	1.04	0.11
polyic	1.68	1.55	1.78	1.67	0.12
polyic+sh-mavs	0.89	1.24	1.14	1.09	0.18

Fig S3-i: VSV mRNA level						
		1	2	3	AVE	STDEV
A549	0	1.00	1.15	1.23	1.13	0.12
	12	1.05	2.35	1.58	1.66	0.65
	24	2.85	2.54	3.25	2.88	0.36
	36	5.53	5.27	6.35	5.72	0.56
	48	5.82	6.74	6.85	6.47	0.57
Hela	0	1.00	1.17	2.35	1.51	0.74
	12	1.27	1.75	1.08	1.37	0.35
	24	3.13	3.69	3.78	3.53	0.35
	36	4.43	4.85	5.96	5.08	0.79
	48	4.39	3.25	6.57	4.74	1.69

S3-j: SeV mRNA level						
		1	2	3	AVE	STDEV
A549	0	1.02	1.02	2.35	1.46	0.77
	12	2.44	2.09	3.17	2.57	0.55
	24	4.61	3.83	5.80	4.75	0.99
	36	9.39	7.76	11.81	9.65	2.04
	48	10.82	11.74	12.96	11.84	1.07
Hela	0	1.00	1.27	2.34	1.54	0.71
	12	1.91	2.54	1.25	1.90	0.65
	24	4.42	3.87	4.09	4.13	0.28
	36	4.88	4.36	4.39	4.54	0.29
	48	6.54	7.72	5.63	6.63	1.05

Fig S3-k:IAV mRNA level						
		1	2	3	AVE	STDEV
A549	0	1.00	1.54	2.48	1.67	0.75
	12	1.27	1.39	2.43	1.70	0.64
	24	3.78	3.64	2.94	3.45	0.45
	36	6.07	5.94	7.57	6.53	0.91
	48	7.14	6.61	7.26	7.00	0.35
Hela	0	1.01	1.74	2.17	1.64	0.59
	12	1.45	2.09	1.87	1.80	0.33
	24	2.20	2.14	3.03	2.46	0.50
	36	3.40	3.73	4.64	3.92	0.64
	48	6.50	5.69	7.15	6.45	0.73

Fig S3-l: mRNA level						
		1	2	3	AVE	STDEV
gfpt1	ctrl	1.00	1.62	1.89	1.19	0.17
	polyic	1.25	0.89	1.74	9.76	1.53
	polyic+sh-mavs	1.32	1.48	1.62	3.00	0.83
gfpt2	ctrl	1.00	1.24	1.33	1.19	0.17
	polyic	8.29	9.63	11.35	9.76	1.53
	polyic+sh-mavs	3.25	2.07	3.67	3.00	0.83

Fig S3-m: GFPT activity						
		1	2	3	AVE	STDEV
sh-ctrl	0	1.00	1.05	1.07	1.04	0.04
	1	1.42	1.58	1.61	1.54	0.10
	3	1.86	1.92	2.14	1.97	0.15
	6	2.04	2.23	2.36	2.21	0.16
sh-MAVS	0	0.94	1.05	1.01	1.00	0.06
	1	0.86	1.13	1.09	1.03	0.15
	3	1.02	1.15	1.17	1.11	0.08
	6	1.18	1.23	1.27	1.23	0.05

Fig s3n

(i)-(iii) :The samples were separated two gels, and the membranes were cut and incubated with the indicated antibodies.

(i) anti- O-GlcNAc

(ii) anti-MAVS

(iii) anti-β-actin

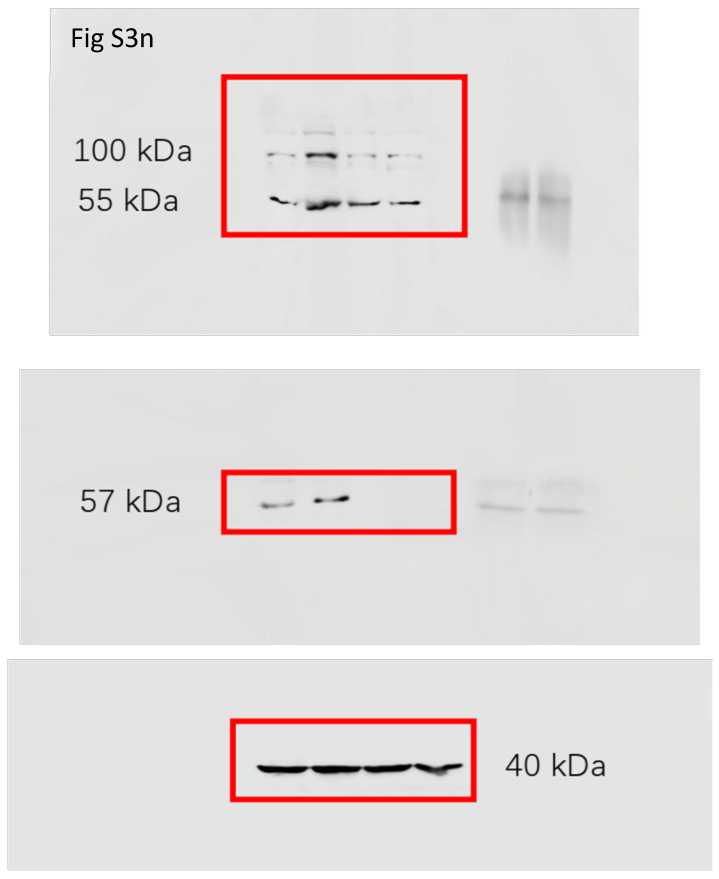

S3-o: UDP-GlcNAc level						
		1	2	3	AVE	STDEV
gfpt1	ctrl	1.00	0.89	1.35	1.08	0.24
	polyic	1.75	1.69	1.88	1.77	0.10
	polyic+sh-mavs	1.14	1.23	1.04	1.14	0.10

Fig S4-d: mRNA level

		1	2	3	AVE	STDEV
Glut1	ctrl	1.00	1.73	1.59	1.44	0.39
	cyto	0.89	1.54	1.61	1.35	0.40
	wt	10.91	9.07	7.19	9.06	1.86
	mito	6.28	7.19	8.33	7.27	1.03
	pex	3.29	4.95	6.19	4.81	1.46
	mam	3.09	4.18	2.97	3.41	0.67
Glut4	ctrl	1.00	1.48	1.63	1.37	0.33
	cyto	0.91	1.27	1.92	1.37	0.51
	wt	7.91	8.99	6.17	7.69	1.42
	mito	6.38	7.84	7.22	7.15	0.73
	pex	4.29	5.91	4.47	4.89	0.89
	mam	2.19	3.94	4.91	3.68	1.38

Fig S4-e: HK activity

	1	2	3	AVE	STDEV
ctrl	1.00	1.05	1.09	1.05	0.05
cyto	0.95	1.09	1.12	1.05	0.09
wt	1.78	1.83	1.62	1.74	0.11
mito	1.57	1.66	1.52	1.58	0.07
pex	1.25	0.98	0.86	1.03	0.20
mam	1.33	1.14	0.82	1.10	0.26

Fig S4-f: NADPH level

	1	2	3	AVE	STDEV
ctrl	1.00	1.10	1.06	1.05	0.05
cyto	1.12	0.86	0.92	0.97	0.14
wt	1.53	1.66	1.48	1.56	0.09
mito	0.95	1.08	1.04	1.02	0.07
pex	1.83	1.92	1.77	1.84	0.08
mam	0.89	1.08	0.93	0.97	0.10

Fig S4-g: NADP+/NADPH

	1	2	3	AVE	STDEV
ctrl	1.00	1.05	1.08	1.04	0.04
cyto	0.95	1.14	0.83	0.97	0.16
wt	0.53	0.44	0.61	0.53	0.09
mito	1.07	0.99	0.83	0.96	0.12
pex	0.32	0.28	0.45	0.35	0.09
mam	1.06	0.92	0.86	0.95	0.10

Figs4c group1-4

Group 1 Mitochondria

(i)-(v) : The same samples were separated two gels, and the membranes were cut and incubated with the indicated antibodies.

Group 2 Peroxisome

(i)-(v) : The same samples were separated two gels, and the membranes were cut and incubated with the indicated antibodies.

Group 3 MAM

(i)-(v) : The same samples were separated two gels, and the membranes were cut and incubated with the indicated antibodies.

Group 4 Cytosol

(i)-(v) : The same samples were separated two gels, and the membranes were cut and incubated with the indicated antibodies.

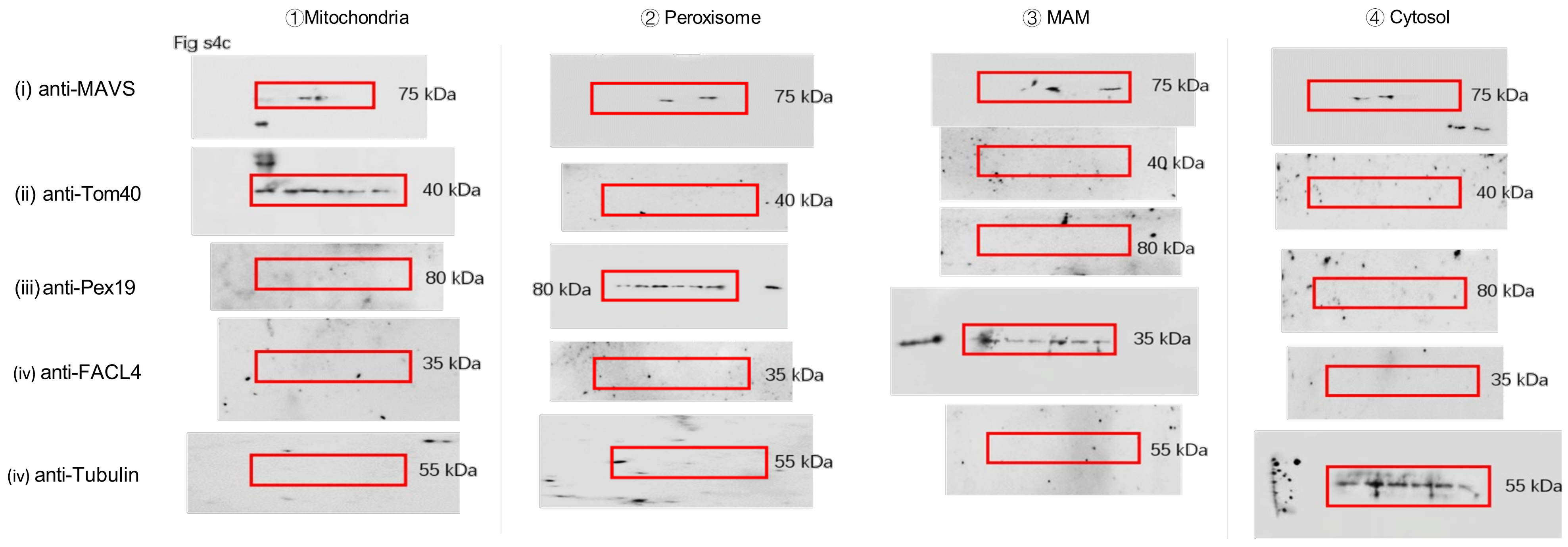

Fig S5-a: G6P level										
	M2					M0				
	1	2	3	AVE	STDEV	1	2	3	AVE	STDEV
ctrl	41.05	43.99	34.26	39.77	4.99	58.69	63.24	66.17	62.70	3.77
cyto	38.29	46.35	44.14	42.93	4.16	55.81	64.95	62.38	61.05	4.71
wt	82.57	86.90	74.58	81.35	6.25	14.75	21.96	26.35	21.02	5.86
mito	65.68	73.22	67.39	68.76	3.95	32.35	26.01	38.78	32.38	6.39
pex	22.07	17.91	25.22	21.73	3.67	73.08	80.14	86.92	80.05	6.92
mam	43.69	47.28	35.96	42.31	5.78	53.25	63.99	67.25	61.50	7.33

Fig S5-b: F6P level										
	M2					M0				
	1	2	3	AVE	STDEV	1	2	3	AVE	STDEV
ctrl	40.25	46.87	48.96	45.36	4.55	52.39	57.92	61.27	57.19	4.48
cyto	38.29	50.96	49.33	46.19	6.89	49.68	63.07	53.79	55.51	6.86
wt	82.07	78.92	75.39	78.79	3.34	28.29	23.04	15.74	22.36	6.30
mito	76.35	65.14	70.18	70.56	5.61	21.05	36.38	33.47	30.30	8.14
pex	21.75	26.38	20.14	22.76	3.24	78.29	85.14	70.29	77.91	7.43
mam	27.28	29.54	22.38	26.40	3.66	78.19	80.91	72.07	77.06	4.53

Fig S5-c: F1,6P level										
	M2					M0				
	1	2	3	AVE	STDEV	1	2	3	AVE	STDEV
ctrl	63.05	66.18	60.25	63.16	2.97	34.28	41.32	36.33	37.31	3.62
cyto	67.18	60.18	57.81	61.72	4.87	33.74	42.30	35.19	37.08	4.58
wt	92.28	98.33	90.14	93.58	4.25	7.25	9.36	4.25	6.95	2.57
mito	88.17	92.17	96.17	92.17	4.00	12.47	2.38	9.35	8.07	5.17
pex	40.25	43.29	47.25	43.60	3.51	63.04	57.84	51.17	57.35	5.95
mam	38.29	35.71	41.57	38.52	2.94	62.35	57.18	65.08	61.54	4.01

Fig S5-d: Pyruvate level															
	M2					M1					M0				
	1	2	3	AVE	STDEV	1	2	3	AVE	STDEV	1	2	3	AVE	STDEV
ctrl	57.44	63.11	66.95	62.50	4.78	24.18	18.92	22.25	21.78	2.66	14.57	18.95	23.04	18.85	4.24
cyto	62.05	54.17	64.33	60.18	5.33	15.27	23.69	26.39	21.78	5.80	25.17	18.24	17.99	20.47	4.08
wt	92.35	84.57	96.33	91.08	5.98	2.35	3.69	4.78	3.61	1.22	9.68	12.30	1.08	7.69	5.87
mito	93.66	82.50	80.39	85.52	7.13	5.36	6.99	7.18	6.51	1.00	12.39	10.27	9.38	10.68	1.55
pex	32.81	30.17	28.09	30.36	2.37	12.35	7.24	6.39	8.66	3.22	68.24	57.98	65.05	63.76	5.25
mam	24.57	36.05	31.21	30.61	5.76	14.17	6.37	9.36	9.97	3.94	63.19	56.38	68.04	62.54	5.86

Fig S5-e: Lactate level															
	M2					M1					M0				
	1	2	3	AVE	STDEV	1	2	3	AVE	STDEV	1	2	3	AVE	STDEV
ctrl	42.05	37.18	35.66	38.30	3.34	30.28	26.39	32.74	29.80	3.20	27.95	33.74	34.15	31.95	3.47
cyto	43.11	36.09	40.71	39.97	3.57	32.04	33.61	27.15	30.93	3.37	26.14	31.75	33.09	30.33	3.69
wt	72.11	74.96	67.18	71.42	3.94	12.28	8.36	20.14	13.59	6.00	9.61	17.25	14.74	13.87	3.89
mito	86.47	74.15	82.18	80.93	6.25	11.04	8.25	5.36	8.22	2.84	12.36	5.01	8.87	8.75	3.68
pex	18.47	23.68	22.04	21.40	2.66	12.07	8.24	7.78	9.36	2.36	66.10	71.08	65.69	67.62	3.00
mam	24.17	17.84	21.06	21.02	3.17	5.18	9.91	14.25	9.78	4.54	70.18	68.54	72.19	70.30	1.83

Fig S5-f: Fumarate level															
	M2					M1					M0				
	1	2	3	AVE	STDEV	1	2	3	AVE	STDEV	1	2	3	AVE	STDEV
ctrl	17.88	23.04	19.65	20.19	2.62	24.58	26.04	14.57	21.73	6.24	57.58	52.96	66.14	58.89	6.69
cyto	16.38	18.92	24.51	19.94	4.16	22.04	17.84	24.95	21.61	3.57	55.95	58.24	64.09	59.43	4.20
wt	34.15	36.25	33.01	34.47	1.64	30.24	41.25	33.25	34.91	5.69	34.28	31.26	26.71	30.75	3.81
mito	47.25	36.35	37.15	40.25	6.08	45.29	41.05	33.96	40.10	5.72	18.29	13.04	27.18	19.50	7.15
pex	2.26	7.26	1.08	3.53	3.28	2.25	3.96	4.71	3.64	1.26	97.41	90.24	88.79	92.15	4.62
mam	1.08	6.38	7.11	4.86	3.29	3.27	6.64	5.07	4.99	1.69	92.17	95.88	85.35	91.13	5.34

Fig S5-g: GAP level															
	M2					M1					M0				
	1	2	3	AVE	STDEV	1	2	3	AVE	STDEV	1	2	3	AVE	STDEV
ctrl	37.14	41.84	43.99	40.99	3.50	12.71	8.29	4.18	8.39	4.27	32.85	48.96	51.99	44.60	10.29
cyto	36.04	42.33	44.95	41.11	4.58	6.18	11.17	7.09	8.15	2.66	36.19	46.33	53.08	45.20	8.50
wt	62.74	64.29	68.25	65.09	2.84	28.25	15.87	19.04	21.05	6.43	7.17	3.66	11.74	7.52	4.05
mito	36.71	42.95	38.19	39.28	3.26	6.19	11.75	12.97	10.30	3.61	37.91	43.05	54.96	45.31	8.75
pex	74.18	86.29	80.25	80.24	6.06	4.87	6.93	13.25	8.35	4.37	12.48	4.17	3.05	6.57	5.15
mam	34.15	45.67	47.81	42.54	7.35	13.07	5.91	7.84	8.94	3.70	36.19	42.81	47.26	42.09	5.57

Fig S5-h: R5P level															
	M2					M1					M0				
	1	2	3	AVE	STDEV	1	2	3	AVE	STDEV	1	2	3	AVE	STDEV
ctrl	17.18	22.95	24.96	21.70	4.04	16.74	19.25	23.67	19.89	3.51	54.18	52.68	68.29	58.38	8.61
cyto	14.28	23.95	26.17	21.47	6.32	15.75	16.97	27.18	19.97	6.28	57.18	55.63	66.96	59.92	6.14
wt	38.29	32.38	44.71	38.46	6.17	34.75	38.68	36.31	36.58	1.98	20.74	28.09	26.19	25.01	3.82
mito	21.74	23.96	18.09	21.26	2.96	17.84	23.61	15.08	18.84	4.35	56.09	59.36	63.04	59.50	3.48
pex	42.29	35.18	47.15	41.54	6.02	47.57	43.94	40.55	44.02	3.51	17.84	13.26	9.36	13.49	4.24
mam	17.54	13.29	27.25	19.36	7.16	16.27	23.04	21.77	20.36	3.60	51.17	67.25	59.35	59.26	8.04

Fig S5-i: ATP level															
	M2					M1					M0				
	1	2	3	AVE	STDEV	1	2	3	AVE	STDEV	1	2	3	AVE	STDEV
ctrl	11.74	5.92	13.26	10.31	3.87	15.68	17.05	19.57	17.43	1.97	68.19	72.15	79.34	73.23	5.65
cyto	12.57	8.29	16.74	12.53	4.23	11.08	14.78	16.35	14.07	2.71	77.09	74.18	76.25	75.84	1.50
wt	21.74	16.37	25.68	21.26	4.67	13.78	18.29	27.15	19.74	6.80	54.28	63.08	67.15	61.50	6.58
mito	37.18	39.33	34.25	36.92	2.55	30.75	27.28	29.04	29.02	1.74	41.25	34.94	36.25	37.48	3.33
pex	28.19	34.15	32.09	31.48	3.03	26.38	27.99	34.18	29.52	4.12	41.28	45.29	37.18	41.25	4.06
mam	8.19	10.57	2.99	7.25	3.88	17.19	8.09	13.67	12.98	4.59	80.95	73.39	88.92	81.09	7.77

Fig S6-a: LC-MS

		M1					M0				
		1	2	3	AVE	STDEV	1	2	3	AVE	STDEV
GlcN-6P	ctrl	42.18	38.29	46.99	42.49	4.36	59.21	55.24	62.35	58.93	3.56
	cyto	32.85	45.29	42.71	40.28	6.57	58.18	69.07	52.11	59.79	8.59
	wt	72.18	77.18	82.26	77.21	5.04	24.16	26.98	20.17	23.77	3.42
	mito	36.91	41.28	47.11	41.77	5.12	52.07	58.99	61.04	57.37	4.70
	pex	33.74	40.14	49.25	41.04	7.79	53.04	62.07	66.35	60.49	6.79
	mam	83.29	90.22	88.19	87.23	3.56	12.39	8.29	17.18	12.62	4.45
ManNAc	ctrl	23.74	18.20	17.25	19.73	3.51	88.27	79.26	74.28	80.60	7.09
	cyto	21.05	16.38	24.85	20.76	4.24	85.74	78.26	76.19	80.06	5.02
	wt	40.29	37.28	46.93	41.50	4.94	67.18	55.39	50.17	57.58	8.71
	mito	17.94	23.68	24.57	22.06	3.60	83.08	70.19	85.96	79.74	8.40
	pex	22.71	26.35	17.74	22.27	4.32	76.39	71.18	87.29	78.29	8.22
	mam	64.25	67.28	61.28	64.27	3.00	38.25	44.18	30.07	37.50	7.08
GlcNAc-6P	ctrl	40.86	37.18	45.11	41.05	3.97	63.27	51.19	57.18	57.21	6.04
	cyto	33.75	41.27	37.18	37.40	3.76	60.19	68.17	55.04	61.13	6.62
	wt	65.79	72.96	75.01	71.25	4.84	18.71	29.35	32.25	26.77	7.13
	mito	36.19	46.17	39.06	40.47	5.14	55.98	63.79	50.05	56.61	6.89
	pex	30.28	37.25	49.50	39.01	9.73	67.74	57.14	51.38	58.75	8.30
	mam	82.04	88.17	95.68	88.63	6.83	6.38	11.47	5.92	7.92	3.08
Neu5Ac	ctrl	12.96	15.36	18.29	15.54	2.67	82.36	88.26	90.24	86.95	4.10
	cyto	8.29	16.38	12.33	12.33	4.05	91.75	86.68	87.99	88.81	2.63
	wt	32.17	27.29	37.99	32.48	5.36	71.28	68.29	60.17	66.58	5.75
	mito	6.19	27.18	10.26	14.54	11.13	91.72	83.63	84.11	86.49	4.54
	pex	11.36	22.06	8.26	13.89	7.24	93.68	85.27	80.99	86.65	6.46
	mam	42.36	37.19	46.78	42.11	4.80	57.25	52.19	61.07	56.84	4.45
JDP-GlcNA	ctrl	30.15	32.96	38.09	33.73	4.03	60.28	67.74	70.95	66.32	5.47
	cyto	28.36	37.18	40.26	35.27	6.18	54.29	66.98	68.09	63.12	7.67
	wt	71.99	65.39	78.19	71.86	6.40	21.55	31.96	28.96	27.49	5.36
	mito	32.78	29.36	39.25	33.80	5.02	61.05	66.95	70.28	66.09	4.67
	pex	31.77	37.89	39.11	36.26	3.93	53.79	69.17	71.44	64.80	9.60
	mam	83.29	88.14	74.95	82.13	6.67	8.29	23.67	14.79	15.58	7.72

Fig S6-b: metabolomics quantify metabolites

		1	2	3	AVE	STDEV
G6P	ctrl	1.00	1.05	1.14	1.06	0.07
	cyto	1.04	1.07	1.12	1.08	0.04
	wt	1.87	1.92	2.17	1.99	0.16
	mito	2.07	2.11	2.14	2.11	0.04
	pex	0.73	0.77	0.68	0.73	0.05
	mam	1.01	0.89	0.94	0.95	0.06
F1,6P	ctrl	1.00	1.25	1.02	1.09	0.14
	cyto	0.86	1.14	0.92	0.97	0.15
	wt	1.85	1.73	1.97	1.85	0.12
	mito	2.17	2.26	2.31	2.25	0.07
	pex	0.42	0.66	0.35	0.48	0.16
	mam	0.62	0.31	0.47	0.47	0.16
6PG	ctrl	1.00	1.02	1.14	1.05	0.08
	cyto	0.86	0.92	1.04	0.94	0.09
	wt	2.04	1.82	1.89	1.92	0.11
	mito	0.92	0.81	1.05	0.93	0.12
	pex	2.17	2.25	2.08	2.17	0.09
	mam	0.98	1.05	0.83	0.95	0.11
GlcNAc-6P	ctrl	1.00	1.05	1.07	1.04	0.04
	cyto	0.88	0.94	1.17	1.00	0.15
	wt	1.58	1.69	1.75	1.67	0.09
	mito	0.93	1.03	1.09	1.02	0.08
	pex	0.94	1.11	0.99	1.01	0.09
	mam	2.24	2.39	2.41	2.35	0.09
F6P	ctrl	1.00	1.11	1.06	1.06	0.06
	cyto	0.89	1.24	1.13	1.09	0.18
	wt	1.92	1.89	2.03	1.95	0.07
	mito	2.17	1.74	2.24	2.05	0.27
	pex	0.59	0.63	0.47	0.56	0.08
	mam	0.66	0.57	0.52	0.58	0.07
3PG	ctrl	1.00	1.04	1.01	1.02	0.02
	cyto	0.96	1.12	0.86	0.98	0.13
	wt	1.83	1.77	1.92	1.84	0.08
	mito	1.67	1.93	2.08	1.89	0.21
	pex	0.57	0.62	0.51	0.57	0.06
	mam	0.45	0.55	0.47	0.49	0.05
R5P	ctrl	1.00	1.04	1.07	1.04	0.04
	cyto	0.96	0.86	1.09	0.97	0.12
	wt	1.86	1.73	1.69	1.76	0.09
	mito	1.03	0.88	1.06	0.99	0.10
	pex	1.93	2.14	1.80	1.96	0.17
	mam	0.92	1.01	1.11	1.01	0.10
GlcNAc-1P	ctrl	1.00	1.08	1.12	1.07	0.06
	cyto	0.96	1.04	0.89	0.96	0.08
	wt	1.75	1.63	1.89	1.76	0.13
	mito	0.82	0.96	1.07	0.95	0.13
	pex	0.87	0.97	0.80	0.88	0.09
	mam	2.09	2.33	2.18	2.20	0.12

		1	2	3	AVE	STDEV
Pyruvate	ctrl	1.00	1.05	1.17	1.07	0.09
	cyto	0.92	1.13	0.97	1.01	0.11
	wt	1.67	1.44	1.72	1.61	0.15
	mito	1.83	1.78	1.88	1.83	0.05
	pex	0.49	0.66	0.48	0.54	0.10
	mam	0.61	0.73	0.55	0.63	0.09
S7P	ctrl	1.00	1.08	1.12	1.07	0.06
	cyto	0.92	1.14	0.82	0.96	0.16
	wt	1.89	1.92	1.72	1.84	0.11
	mito	0.91	0.88	1.09	0.96	0.11
	pex	1.69	1.77	1.84	1.77	0.08
	mam	0.96	1.04	1.14	1.05	0.09
JDP-GlcNA	ctrl	1.00	1.05	1.12	1.06	0.06
	cyto	0.87	0.93	1.09	0.96	0.11
	wt	1.93	1.77	1.82	1.84	0.08
	mito	0.93	0.86	0.74	0.84	0.10
	pex	0.77	0.92	1.08	0.92	0.16
	mam	2.89	3.05	2.57	2.84	0.24
Lactate	ctrl	1.00	1.05	1.12	1.06	0.06
	cyto	0.96	1.07	0.86	0.96	0.11
	wt	2.17	2.31	2.24	2.24	0.07
	mito	2.08	1.93	2.51	2.17	0.30
	pex	0.35	0.47	0.69	0.50	0.17
	mam	0.39	0.44	0.51	0.45	0.06
GSH	ctrl	1.00	1.05	1.14	1.06	0.07
	cyto	0.89	1.14	0.93	0.99	0.13
	wt	1.93	1.77	1.68	1.79	0.13
	mito	0.85	0.73	1.28	0.95	0.29
	pex	1.68	1.89	1.92	1.83	0.13
	mam	1.17	0.79	0.83	0.93	0.21
Succinate	ctrl	1.00	1.05	1.01	1.02	0.03
	cyto	0.92	1.24	0.89	1.02	0.19
	wt	1.62	1.47	1.74	1.61	0.14
	mito	1.59	1.67	1.73	1.66	0.07
	pex	0.59	0.41	0.68	0.56	0.14
	mam	0.51	0.42	0.48	0.47	0.05
GlcN-6P	ctrl	1.00	1.04	1.09	1.04	0.05
	cyto	0.92	0.86	1.17	0.98	0.16
	wt	1.86	1.72	2.09	1.89	0.19
	mito	0.86	0.79	1.24	0.96	0.24
	pex	1.05	0.92	0.97	0.98	0.07
	mam	2.07	2.11	2.36	2.18	0.16

Fig S7-a: mRNA level							
			1	2	3	AVE	STDEV
IFN-β	Medium	Ctrl	1.25	4.28	7.26	4.26	3.01
		VSV	392.25	418.28	449.36	419.96	28.59
	6-AN	ctrl	5.26	14.33	11.95	10.51	4.70
		VSV	368.29	395.02	421.84	395.05	26.78
IFN-λ1	Medium	Ctrl	1.28	4.95	9.25	5.16	3.99
		VSV	126.35	145.27	162.36	144.66	18.01
	6-AN	ctrl	9.77	12.25	17.25	13.09	3.81
		VSV	13.26	24.02	7.14	14.81	8.55
IL-6	Medium	Ctrl	1.00	6.35	2.09	3.15	2.83
		VSV	217.25	263.25	248.29	242.93	23.46
	6-AN	ctrl	6.74	8.95	4.17	6.62	2.39
		VSV	56.47	82.25	34.15	57.62	24.07
TNF-α	Medium	Ctrl	1.00	6.77	12.36	6.71	5.68
		VSV	85.26	79.36	105.36	89.99	13.63
	6-AN	ctrl	4.74	9.36	13.05	9.05	4.16
		VSV	23.69	41.27	20.81	28.59	11.08
IL1-β	Medium	Ctrl	1.00	2.36	7.25	3.54	3.29
		VSV	153.24	178.25	104.33	145.27	37.60
	6-AN	ctrl	4.18	6.33	9.14	6.55	2.49
		VSV	53.26	41.05	63.06	52.46	11.03

Fig S7-b: mRNA level							
			1	2	3	AVE	STDEV
IFN-β	Medium	Ctrl	1.00	4.71	6.58	4.10	2.84
		VSV	427.68	433.96	407.26	422.97	13.96
	OSMI-1	ctrl	14.25	6.35	2.57	7.72	5.96
		VSV	189.25	217.26	227.36	211.29	19.74
IFN-λ1	Medium	Ctrl	1.00	3.47	9.36	4.61	4.30
		VSV	147.26	128.69	158.02	144.66	14.84
	OSMI-1	ctrl	2.05	4.96	5.78	4.26	1.96
		VSV	140.62	159.36	137.05	145.68	11.98
IL-6	Medium	Ctrl	1.00	3.67	7.91	4.19	3.48
		VSV	217.69	189.24	193.26	200.06	15.40
	OSMI-1	ctrl	2.47	5.28	7.19	4.98	2.37
		VSV	78.25	59.36	67.25	68.29	9.49
TNF-α	Medium	Ctrl	1.00	5.28	6.33	4.20	2.82
		VSV	92.57	83.22	70.15	81.98	11.26
	OSMI-1	ctrl	7.15	11.74	3.68	7.52	4.04
		VSV	23.05	31.24	42.99	32.43	10.02
IL1-β	Medium	Ctrl	1.00	2.98	6.33	3.44	2.69
		VSV	136.85	178.29	163.02	159.39	20.96
	OSMI-1	ctrl	10.28	9.35	4.15	7.93	3.30
		VSV	56.38	66.98	49.05	57.47	9.01

Fig s7C

(i)-(V) :The samples were separated two gels, and the membranes were cut and incubated with the indicated antibodies.

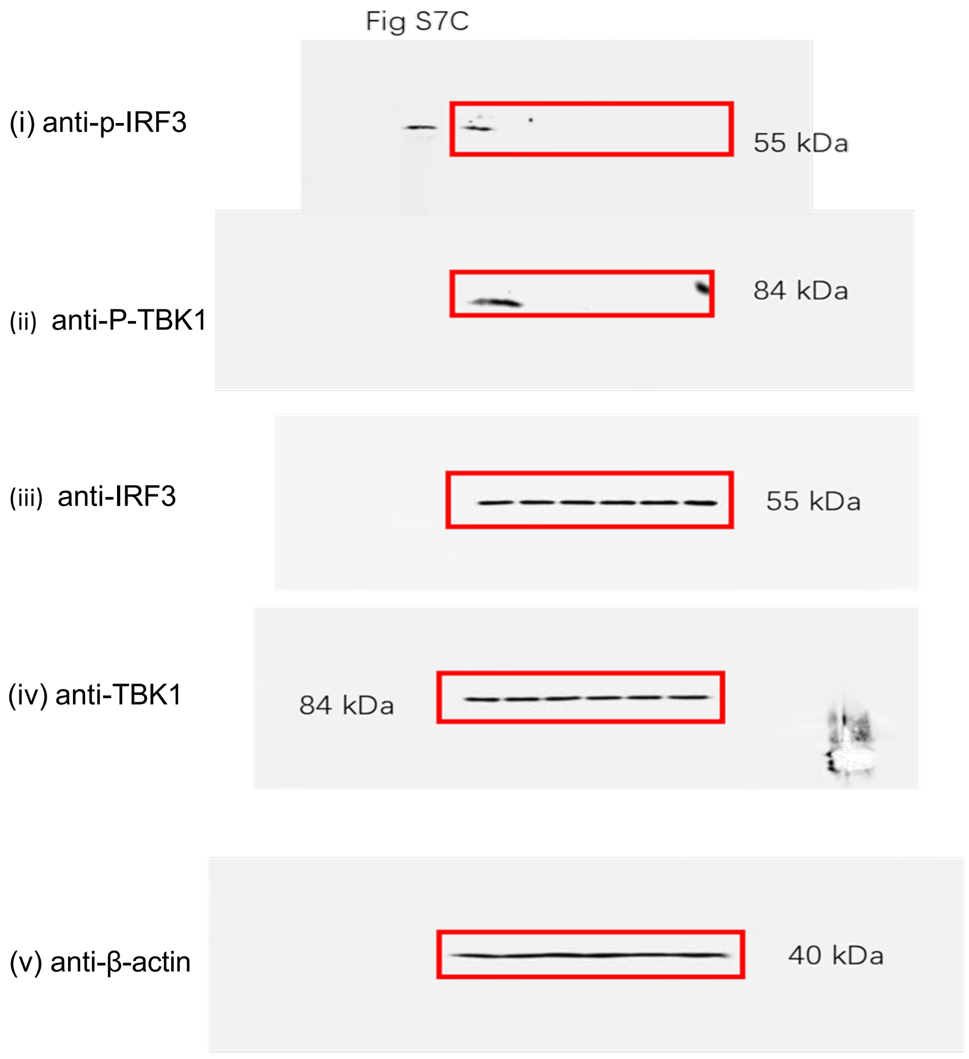

Fig S7-d: VSV replication						
		1	2	3	AVE	STDEV
ui	Medium	0.00	0.00	0.00	0.00	0.00
	G6PDi	0.00	0.00	0.00	0.00	0.00
ctrl	Medium	100.00	103.25	101.95	101.73	1.64
	G6PDi	95.38	105.32	107.24	102.65	6.37
cyto	Medium	93.85	101.02	117.85	104.24	12.32
	G6PDi	83.02	95.36	99.04	92.47	8.39
wt	Medium	41.25	44.29	47.25	44.26	3.00
	G6PDi	82.14	74.89	88.36	81.80	6.74
mito	Medium	42.58	51.25	55.36	49.73	6.52
	G6PDi	50.74	47.63	53.69	50.69	3.03
pex	Medium	38.26	35.26	30.28	34.60	4.03
	G6PDi	92.14	85.24	97.62	91.67	6.20
mam	Medium	41.75	35.96	43.99	40.57	4.14
	G6PDi	48.25	34.28	37.36	39.96	7.34

Fig S7-e: VSV replication						
		1	2	3	AVE	STDEV
ui	Medium	0.00	0.00	0.00	0.00	0.00
	Aza	0.00	0.00	0.00	0.00	0.00
ctrl	Medium	100.00	104.95	93.24	99.40	5.88
	Aza	103.25	95.25	97.05	98.52	4.20
cyto	Medium	97.14	102.73	96.78	98.88	3.34
	Aza	89.36	87.25	91.46	89.36	2.11
wt	Medium	41.36	35.78	39.14	38.76	2.81
	Aza	71.05	77.25	81.24	76.51	5.13
mito	Medium	52.36	48.25	57.93	52.85	4.86
	Aza	42.69	51.24	56.74	50.22	7.08
pex	Medium	32.58	28.69	33.94	31.74	2.72
	Aza	29.36	34.17	38.29	33.94	4.47
mam	Medium	41.28	34.29	33.05	36.21	4.44
	Aza	87.29	94.15	92.04	91.16	3.51

Fig S8-a: mRNA level

		1	2	3	AVE	STDEV
THP-1	ctrl	1.00	1.25	1.06	1.10	0.13
	sh-G6PD #1	0.24	0.16	0.09	0.16	0.08
	sh-G6PD#2	0.34	0.29	0.22	0.28	0.06
BMDMs	ctrl	1.00	1.24	1.51	1.25	0.26
	sh-G6PD #1	0.09	0.10	0.07	0.09	0.02
	sh-G6PD#2	0.26	0.34	0.18	0.26	0.08

Fig S8-b: mRNA level

		1	2	3	AVE	STDEV
THP-1	ctrl	1.00	1.11	1.29	1.13	0.15
	sh-GFPT2#1	0.08	0.15	0.24	0.16	0.08
	sh-GFPT2#2	0.34	0.27	0.19	0.27	0.08
BMDMs	ctrl	1.00	1.52	1.74	1.42	0.38
	sh-GFPT2#1	0.11	0.06	0.21	0.13	0.08
	sh-GFPT2#2	0.24	0.36	0.19	0.26	0.09

Fig S8-c: mRNA level

			1	2	3	AVE	STDEV
IFN-β	medium	sh-ctrl	1.00	1.26	1.37	1.21	0.19
		sh-g6pd	0.89	1.63	2.84	1.79	0.98
	ployic	sh-ctrl	23.81	26.98	20.74	23.84	3.12
		sh-g6pd	19.35	24.85	26.33	23.51	3.68
IFN-λ	medium	sh-ctrl	1.00	1.63	1.95	1.53	0.48
		sh-g6pd	0.89	1.47	2.36	1.57	0.74
	ployic	sh-ctrl	13.85	11.74	8.54	11.38	2.67
		sh-g6pd	2.14	2.93	1.04	2.04	0.95
IL-6	medium	sh-ctrl	1.00	2.04	1.33	1.46	0.53
		sh-g6pd	1.63	1.27	2.96	1.95	0.89
	ployic	sh-ctrl	18.29	17.22	23.06	19.52	3.11
		sh-g6pd	7.26	9.36	11.75	9.46	2.25
TNF-α	medium	sh-ctrl	1.00	1.75	2.33	1.69	0.67
		sh-g6pd	0.86	1.36	2.35	1.52	0.76
	ployic	sh-ctrl	8.29	11.55	7.14	8.99	2.29
		sh-g6pd	3.26	4.15	2.99	3.47	0.61
IL1-β	medium	sh-ctrl	1.00	1.32	2.41	1.58	0.74
		sh-g6pd	0.88	1.17	1.95	1.33	0.55
	ployic	sh-ctrl	14.26	17.69	12.08	14.68	2.83
		sh-g6pd	5.26	7.19	8.92	7.12	1.83

Fig S8-d: mRNA level

			1	2	3	AVE	STDEV
IFN-β	medium	sh-ctrl	1.00	1.42	1.09	1.17	0.22
		sh-gfpt2	0.89	2.63	1.47	1.66	0.89
	ployic	sh-ctrl	23.85	28.96	21.85	24.89	3.67
		sh-gfpt2	12.38	15.96	14.77	14.37	1.82
IFN-λ	medium	sh-ctrl	1.00	1.36	1.84	1.40	0.42
		sh-gfpt2	0.98	2.04	1.67	1.56	0.54
	ployic	sh-ctrl	12.74	8.29	14.75	11.93	3.31
		sh-gfpt2	10.74	14.94	9.24	11.64	2.95
IL-6	medium	sh-ctrl	1.00	1.05	1.36	1.14	0.20
		sh-gfpt2	0.85	1.63	2.77	1.75	0.97
	ployic	sh-ctrl	23.25	18.47	16.28	19.33	3.56
		sh-gfpt2	1.74	0.96	1.17	1.29	0.40
TNF-α	medium	sh-ctrl	1.00	1.47	2.33	1.60	0.67
		sh-gfpt2	1.14	1.32	1.76	1.41	0.32
	ployic	sh-ctrl	10.78	8.26	7.26	8.77	1.81
		sh-gfpt2	0.95	0.81	1.24	1.00	0.22
IL1-β	medium	sh-ctrl	1.00	1.63	2.77	1.80	0.90
		sh-gfpt2	0.89	2.05	2.36	1.77	0.77
	ployic	sh-ctrl	12.85	18.29	19.74	16.96	3.63
		sh-gfpt2	0.92	0.79	2.79	1.50	1.12

Fig S8-e: IFNβ level

		1	2	3	AVE	STDEV
sh-ctrl	ui	0.00	0.00	0.00	0.00	0.00
	ctrl	1.00	1.24	1.17	1.14	0.12
	cyto	2.36	1.78	2.04	2.06	0.29
	wt	17.25	20.36	19.37	18.99	1.59
	mito	10.52	9.75	5.26	8.51	2.84
	pex	3.92	5.95	6.07	5.31	1.21
	mam	3.78	4.27	6.79	4.95	1.62
sh-g6pd	ui	0.00	0.00	0.00	0.00	0.00
	ctrl	0.89	1.22	1.37	1.16	0.25
	cyto	2.06	1.47	2.64	2.06	0.59
	wt	21.36	22.98	16.47	20.27	3.39
	mito	7.95	6.08	9.37	7.80	1.65
	pex	4.77	7.26	5.36	5.80	1.30
	mam	5.40	6.39	4.15	5.31	1.12

Fig S8-f: IFNλ level

		1	2	3	AVE	STDEV
sh-ctrl	ui	0.00	0.00	0.00	0.00	0.00
	ctrl	1.25	1.00	1.37	1.21	0.19
	cyto	0.92	0.83	1.17	0.97	0.18
	wt	11.52	9.36	7.28	9.39	2.12
	mito	5.04	6.35	4.89	5.43	0.80
	pex	9.35	8.24	6.27	7.95	1.56
	mam	3.25	2.06	1.28	2.20	0.99
sh-g6pd	ui	0.00	0.00	0.00	0.00	0.00
	ctrl	0.86	1.41	1.07	1.11	0.28
	cyto	0.88	1.42	0.93	1.08	0.30
	wt	3.26	5.28	5.99	4.84	1.42
	mito	5.96	5.14	4.29	5.13	0.84
	pex	1.05	0.86	0.75	0.89	0.15
	mam	3.33	1.95	3.74	3.01	0.94

Fig S8-g: IL6 level

		1	2	3	AVE	STDEV
sh-ctrl	ui	0.00	0.00	0.00	0.00	0.00
	ctrl	1.00	1.18	1.24	1.14	0.12
	cyto	0.78	1.76	0.63	1.06	0.61
	wt	13.54	14.99	17.91	15.48	2.23
	mito	8.24	7.19	9.74	8.39	1.28
	pex	9.35	6.33	8.99	8.22	1.65
	mam	12.63	10.27	13.24	12.05	1.57
sh-g6pd	ui	0.00	0.00	0.00	0.00	0.00
	ctrl	0.92	0.68	1.75	1.12	0.56
	cyto	1.05	1.18	1.93	1.39	0.48
	wt	6.25	7.26	4.11	5.87	1.61
	mito	8.04	8.79	7.62	8.15	0.59
	pex	2.05	1.24	0.86	1.38	0.61
	mam	11.75	12.09	12.91	12.25	0.60

Fig S8-h: TNFα level

		1	2	3	AVE	STDEV
sh-ctrl	ui	0.00	0.00	0.00	0.00	0.00
	ctrl	1.00	1.47	1.66	1.38	0.34
	cyto	0.92	0.71	1.69	1.11	0.52
	wt	6.74	9.35	6.01	7.37	1.76
	mito	4.06	3.86	4.97	4.30	0.59
	pex	8.15	11.42	9.07	9.55	1.69
	mam	9.36	10.24	8.20	9.27	1.02
sh-g6pd	ui	0.00	0.00	0.00	0.00	0.00
	ctrl	0.85	0.63	1.78	1.09	0.61
	cyto	1.36	1.78	0.62	1.25	0.59
	wt	2.36	3.04	4.11	3.17	0.88
	mito	3.05	4.15	5.99	4.40	1.49
	pex	2.05	1.14	0.78	1.32	0.65
	mam	7.05	12.24	6.89	8.73	3.04

Fig S8-i: IL1-β level

		1	2	3	AVE	STDEV
sh-ctrl	ui	0.00	0.00	0.00	0.00	0.00
	ctrl	1.00	1.24	1.69	1.31	0.35
	cyto	0.65	1.86	2.04	1.52	0.76
	wt	12.06	8.09	7.88	9.34	2.36
	mito	5.28	3.96	6.98	5.41	1.51
	pex	7.15	9.36	6.47	7.66	1.51
	mam	14.25	13.85	10.25	12.78	2.20
sh-g6pd	ui	0.00	0.00	0.00	0.00	0.00
	ctrl	0.74	0.56	1.89	1.06	0.72
	cyto	1.14	1.47	1.79	1.47	0.33
	wt	3.25	4.15	4.98	4.13	0.87
	mito	3.74	4.95	7.58	5.42	1.96
	pex	1.25	2.33	0.87	1.48	0.76
	mam	15.87	12.95	13.07	13.96	1.65

Fig S8-j: IFNβ level

		1	2	3	AVE	STDEV
sh-ctrl	ui	0.00	0.00	0.00	0.00	0.00
	ctrl	1.00	1.24	1.85	1.36	0.44
	cyto	2.05	2.14	1.86	2.02	0.14
	wt	17.81	19.36	16.33	17.83	1.52
	mito	11.86	7.26	6.38	8.50	2.94
	pex	9.25	8.75	8.19	8.73	0.53
	mam	4.33	3.08	4.96	4.12	0.96
sh-gfpt2	ui	0.00	0.00	0.00	0.00	0.00
	ctrl	0.86	0.71	1.75	1.11	0.56
	cyto	2.37	1.08	2.86	2.10	0.92
	wt	9.31	7.74	5.36	7.47	1.99
	mito	10.89	8.26	9.33	9.49	1.32
	pex	10.25	7.96	7.05	8.42	1.65
	mam	0.97	0.86	1.12	0.98	0.13

Fig S8-k: IFNλ level

		1	2	3	AVE	STDEV
sh-ctrl	ui	0.00	0.00	0.00	0.00	0.00
	ctrl	1.00	1.72	1.24	1.32	0.37
	cyto	1.14	0.86	0.75	0.92	0.20
	wt	13.85	11.04	14.71	13.20	1.92
	mito	8.29	10.74	11.24	10.09	1.58
	pex	8.29	5.27	9.33	7.63	2.11
	mam	4.05	3.88	4.79	4.24	0.48
sh-gfpt2	ui	0.00	0.00	0.00	0.00	0.00
	ctrl	0.85	1.75	0.77	1.12	0.54
	cyto	1.32	0.91	1.75	1.33	0.42
	wt	15.91	12.86	14.05	14.27	1.54
	mito	12.85	7.14	7.94	9.31	3.09
	pex	7.18	10.74	8.72	8.88	1.79
	mam	5.29	3.14	3.72	4.05	1.11

Fig S8-l: IL6 level

		1	2	3	AVE	STDEV
sh-ctrl	ui	0.00	0.00	0.00	0.00	0.00
	ctrl	1.00	1.24	1.39	1.21	0.20
	cyto	1.04	0.77	0.69	0.83	0.18
	wt	9.25	12.35	7.98	9.86	2.25
	mito	9.25	6.33	6.09	7.22	1.76
	pex	6.38	9.35	7.04	7.59	1.56
	mam	19.36	14.25	17.21	16.94	2.57
sh-gfpt2	ui	0.00	0.00	0.00	0.00	0.00
	ctrl	0.86	0.68	1.95	1.16	0.69
	cyto	1.35	1.75	0.88	1.33	0.44
	wt	4.26	3.11	2.09	3.15	1.09
	mito	7.15	9.35	6.03	7.51	1.69
	pex	6.35	6.08	8.92	7.12	1.57
	mam	2.36	3.74	3.99	3.36	0.88

Fig S8-m: TNFα level

		1	2	3	AVE	STDEV
sh-ctrl	ui	0.00	0.00	0.00	0.00	0.00
	ctrl	1.00	1.14	1.02	1.05	0.08
	cyto	1.35	1.41	0.83	1.20	0.32
	wt	8.26	10.24	9.35	9.28	0.99
	mito	4.26	3.25	2.96	3.49	0.68
	pex	5.33	2.39	2.91	3.54	1.57
	mam	12.36	13.05	10.75	12.05	1.18
sh-gfpt2	ui	0.00	0.00	0.00	0.00	0.00
	ctrl	0.81	0.73	1.89	1.14	0.65
	cyto	0.75	0.99	1.34	1.03	0.30
	wt	2.74	4.26	3.96	3.65	0.81
	mito	4.98	4.18	2.09	3.75	1.49
	pex	4.26	3.74	3.05	3.68	0.61
	mam	1.25	2.96	0.86	1.69	1.12

Fig S8-n: IL1-β level

		1	2	3	AVE	STDEV
sh-ctrl	ui	0.00	0.00	0.00	0.00	0.00
	ctrl	1.00	1.17	1.08	1.08	0.09
	cyto	1.16	1.30	0.75	1.07	0.29
	wt	10.85	16.35	15.27	14.16	2.91
	mito	10.36	8.24	6.38	8.33	1.99
	pex	7.29	9.36	6.89	7.85	1.33
	mam	16.39	19.25	13.85	16.50	2.70
sh-gfpt2	ui	0.00	0.00	0.00	0.00	0.00
	ctrl	0.82	0.68	1.92	1.14	0.68
	cyto	1.32	0.85	1.04	1.07	0.24
	wt	3.86	4.15	5.92	4.64	1.12
	mito	7.26	8.96	9.11	8.44	1.03
	pex	6.48	9.57	9.22	8.42	1.69
	mam	2.05	1.96	3.74	2.58	1.00

Fig s9a-b

(i)-(ii) :The samples were separated one gel, and the membrane was cut and incubated with the indicated antibodies.

(i) anti-AR

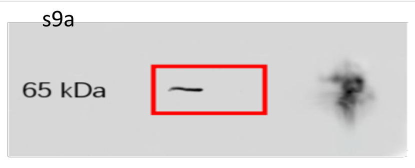

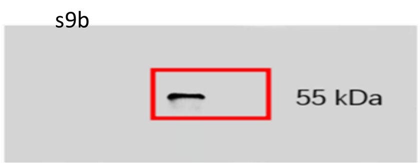

(i) anti-LR

(ii) anti-β-actin

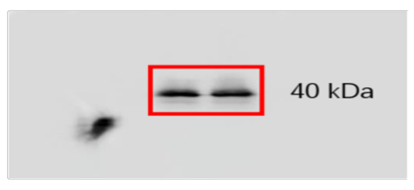

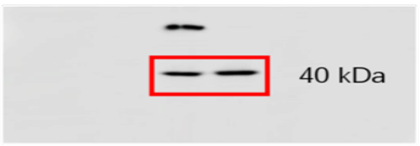

(ii) anti-β-actin

FigS9-c: VSV replication and ISG56 level

			1	2	3	AVE	STDEV
vsv replication	vector	ui	0.00	0.00	0.00	0.00	0.00
		wt	100.00	105.36	101.25	102.20	2.80
		ar-/-	139.25	147.26	150.24	145.58	5.68
		lr-/-	145.05	153.26	168.26	155.52	11.77
	g6pd	ui	0.00	0.00	0.00	0.00	0.00
		wt	34.28	48.64	36.35	39.76	7.76
		ar-/-	46.25	40.25	54.18	46.89	6.99
		lr-/-	138.69	162.03	167.05	155.92	15.13
ISG56	vector	ui	1.00	1.05	1.14	1.06	0.07
		wt	12.75	15.72	17.96	15.48	2.61
		ar-/-	5.18	4.96	8.26	6.13	1.85
		lr-/-	8.96	9.26	11.27	9.83	1.26
	g6pd	ui	3.86	0.93	1.42	2.07	1.57
		wt	41.85	44.26	37.26	41.12	3.56
		ar-/-	23.65	22.74	18.06	21.48	3.00
		lr-/-	12.36	7.29	8.95	9.53	2.58

FigS9-d: VSV replication and ISG56 level

			1	2	3	AVE	STDEV
vsv replication	vector	ui	0.00	0.00	0.00	0.00	0.00
		wt	100.00	105.69	101.48	102.39	2.95
		ar-/-	152.36	142.77	130.25	141.79	11.09
		lr-/-	137.14	142.36	120.75	133.42	11.28
	gfpt2	ui	0.00	0.00	0.00	0.00	0.00
		wt	34.25	49.36	37.23	40.28	8.00
		ar-/-	143.96	151.14	139.05	144.72	6.08
		lr-/-	54.25	47.26	57.99	53.17	5.45
ISG56	vector	ui	1.00	1.17	1.86	1.34	0.46
		wt	12.75	16.96	17.26	15.66	2.52
		ar-/-	11.25	8.17	7.10	8.84	2.15
		lr-/-	8.26	7.26	5.26	6.93	1.53
	gfpt2	ui	0.86	3.04	1.56	1.82	1.11
		wt	32.26	35.09	37.25	34.87	2.50
		ar-/-	12.63	10.25	7.26	10.05	2.69
		lr-/-	15.39	12.35	19.22	15.65	3.44

FigS9-e: VSV replication and ISG56 level

			1	2	3	AVE	STDEV
vsv replication	sh-ctrl	ui	0.00	0.00	0.00	0.00	0.00
		wt	100.00	103.01	101.97	101.66	1.53
		anti-ifnα/β	153.24	167.26	151.07	157.19	8.79
		anti-ifnλ	178.95	180.27	183.64	180.95	2.42
	sh-g6pd	ui	0.00	0.00	0.00	0.00	0.00
		wt	224.14	241.78	249.01	238.31	12.79
		anti-ifnα/β	374.51	369.11	391.74	378.45	11.82
		anti-ifnλ	186.29	177.31	190.22	184.61	6.62
ISG56	sh-ctrl	ui	1.00	1.15	1.92	1.36	0.49
		wt	23.14	19.26	22.07	21.49	2.00
		anti-ifnα/β	11.75	13.95	15.33	13.68	1.81
		anti-ifnλ	16.07	12.95	19.02	16.01	3.04
	sh-g6pd	ui	0.86	2.11	1.24	1.40	0.64
		wt	3.67	2.19	5.01	3.62	1.41
		anti-ifnα/β	3.69	4.18	2.04	3.30	1.12
		anti-ifnλ	21.24	17.94	13.44	17.54	3.92

FigS9-f: VSV replication and ISG56 level

			1	2	3	AVE	STDEV
vsv replication	sh-ctrl	ui	0.00	0.00	0.00	0.00	0.00
		wt	100.00	112.06	104.62	105.56	6.08
		anti-ifnα/β	147.26	158.91	136.08	147.42	11.42
		anti-ifnλ	175.80	189.14	163.26	176.07	12.94
	sh-gfpt2	ui	0.00	0.00	0.00	0.00	0.00
		wt	214.25	186.23	181.31	193.93	17.77
		anti-ifnα/β	167.04	177.95	171.59	172.19	5.48
		anti-ifnλ	275.36	296.34	312.04	294.58	18.40
ISG56	sh-ctrl	ui	1.00	1.05	1.17	1.07	0.09
		wt	23.75	18.95	17.44	20.05	3.29
		anti-ifnα/β	12.41	8.69	14.62	11.91	3.00
		anti-ifnλ	14.25	13.60	17.26	15.04	1.95
	sh-gfpt2	ui	0.95	1.68	2.74	1.79	0.90
		wt	2.69	4.89	5.43	4.34	1.45
		anti-ifnα/β	16.75	17.62	11.59	15.32	3.26
		anti-ifnλ	2.36	1.44	3.07	2.29	0.82

Fig S9-g: VSV replication and ISG56 level

			1	2	3	AVE	STDEV
vsv replication	vector	ui	0.00	0.00	0.00	0.00	0.00
		wt	100.00	106.37	110.42	105.60	5.25
		anti-ifnα/β	117.25	129.24	132.27	126.25	7.94
		anti-ifnλ	128.26	136.35	139.02	134.54	5.60
	g6pd	ui	0.00	0.00	0.00	0.00	0.00
		wt	48.26	39.32	31.02	39.53	8.62
		anti-ifnα/β	45.26	42.15	37.91	41.77	3.69
		anti-ifnλ	142.36	124.62	121.39	129.46	11.29
ISG56	vector	ui	1.00	1.28	2.33	1.54	0.70
		wt	16.35	12.48	17.88	15.57	2.78
		anti-ifnα/β	4.96	7.92	8.22	7.03	1.80
		anti-ifnλ	7.26	6.91	10.25	8.14	1.84
	g6pd	ui	0.86	2.74	1.92	1.84	0.94
		wt	45.96	47.92	42.04	45.31	2.99
		anti-ifnα/β	23.74	26.49	27.11	25.78	1.79
		anti-ifnλ	11.48	7.18	6.70	8.45	2.63

FigS 9-h: VSV replication and ISG56 level

			1	2	3	AVE	STDEV
vsv replication	vector	ui	0.00	0.00	0.00	0.00	0.00
		wt	100.00	102.96	107.84	103.60	3.96
		anti-ifnα/β	138.26	147.92	120.24	135.47	14.05
		anti-ifnλ	125.36	148.26	150.74	141.45	13.99
	gfpt2	ui	0.00	0.00	0.00	0.00	0.00
		wt	39.36	46.01	32.04	39.14	6.99
		anti-ifnα/β	140.71	135.26	121.74	132.57	9.77
		anti-ifnλ	36.09	64.25	51.20	50.51	14.09
ISG56	vector	ui	1.00	1.27	1.75	1.34	0.38
		wt	12.04	11.65	17.26	13.65	3.13
		anti-ifnα/β	12.57	13.26	10.74	12.19	1.30
		anti-ifnλ	8.26	7.26	9.26	8.26	1.00
	gfpt2	ui	2.19	2.31	3.05	2.52	0.47
		wt	45.26	40.28	43.92	43.15	2.58
		anti-ifnα/β	8.29	14.26	13.77	12.11	3.31
		anti-ifnλ	19.28	17.26	20.05	18.86	1.44

Fig S9-i: VSV replication and ISG56 level

			1	2	3	AVE	STDEV
vsv replication	ctrl	ui	0.00	0.00	0.00	0.00	0.00
		ctrl	100.00	105.26	101.89	102.38	2.66
		cyto	97.26	109.26	95.04	100.52	7.65
		wt	35.04	28.19	31.77	31.67	3.43
		mito	42.75	44.19	39.29	42.08	2.52
		pex	51.24	55.96	58.26	55.15	3.58
		mam	48.59	53.92	58.04	53.52	4.74
	g6pd	ui	0.00	0.00	0.00	0.00	0.00
		ctrl	104.95	97.26	95.33	99.18	5.09
		cyto	92.15	94.57	114.26	100.33	12.13
		wt	2.69	7.05	3.94	4.56	2.25
		mito	48.24	40.05	38.24	42.18	5.33
		pex	2.36	7.25	11.27	6.96	4.46
		mam	52.74	59.34	50.22	54.10	4.71
	g6pd+anti-ifnα/β	ui	0.00	0.00	0.00	0.00	0.00
		ctrl	127.62	131.82	136.25	131.90	4.32
		cyto	123.68	127.26	139.36	130.10	8.22
		wt	64.27	59.24	69.24	64.25	5.00
		mito	72.15	76.29	69.35	72.60	3.49
		pex	5.96	10.47	3.75	6.73	3.42
		mam	83.26	86.24	79.15	82.88	3.56
	g6pd+anti-ifnλ	ui	0.00	0.00	0.00	0.00	0.00
		ctrl	139.35	138.26	130.74	136.12	4.69
		cyto	137.29	142.63	132.57	137.50	5.03
		wt	75.98	78.19	82.14	78.77	3.12
		mito	82.33	85.19	88.02	85.18	2.85
		pex	66.24	61.85	53.26	60.45	6.60
		mam	62.04	53.47	56.81	57.44	4.32
ISG56	ctrl	ui	0.00	0.00	0.00	0.00	0.00
		ctrl	1.00	1.05	1.39	1.15	0.21
		cyto	0.86	1.96	2.42	1.75	0.80
		wt	28.26	22.75	25.33	25.45	2.76
		mito	26.75	28.95	27.15	27.62	1.17
		pex	14.26	12.75	16.96	14.66	2.13
		mam	12.85	10.78	16.68	13.44	2.99
	g6pd	ui	0.00	0.00	0.00	0.00	0.00
		ctrl	0.98	2.36	1.47	1.60	0.70
		cyto	2.33	1.18	2.04	1.85	0.60
		wt	62.86	67.99	66.04	65.63	2.59
		mito	24.33	28.91	30.04	27.76	3.02
		pex	42.85	40.96	50.33	44.71	4.96
		mam	13.77	16.08	14.25	14.70	1.22
	g6pd+anti-ifnα/β	ui	0.00	0.00	0.00	0.00	0.00
		ctrl	0.85	0.77	0.69	0.77	0.08
		cyto	0.91	0.83	0.73	0.82	0.09
		wt	14.75	16.77	14.36	15.29	1.29
		mito	4.69	8.04	3.17	5.30	2.49
		pex	47.25	50.63	46.33	48.07	2.26
		mam	2.38	3.69	5.14	3.74	1.38
	g6pd+anti-ifnλ	ui	0.00	0.00	0.00	0.00	0.00
		ctrl	0.93	0.75	1.34	1.01	0.30
		cyto	0.93	1.47	1.56	1.32	0.34
		wt	14.05	19.17	11.96	15.06	3.71
		mito	2.75	8.59	9.11	6.82	3.53
		pex	3.96	8.14	4.26	5.45	2.33
		mam	17.25	10.99	13.47	13.90	3.15

Fig S9-j: VSV replication and ISG56 level

			1	2	3	AVE	STDEV
vsv replication	ctrl	ui	0.00	0.00	0.00	0.00	0.00
		ctrl	100.00	108.26	104.63	104.30	4.14
		cyto	99.25	105.96	92.35	99.19	6.81
		wt	32.15	28.24	38.11	32.83	4.97
		mito	42.84	44.19	40.27	42.43	1.99
		pex	52.14	48.27	44.68	48.36	3.73
		mam	47.18	52.37	56.33	51.96	4.59
	gfpt2	ui	0.00	0.00	0.00	0.00	0.00
		ctrl	95.36	107.26	96.31	99.64	6.61
		cyto	114.26	92.36	90.27	98.96	13.29
		wt	2.86	8.29	7.61	6.25	2.96
		mito	39.26	47.26	43.92	43.48	4.02
		pex	52.39	57.16	55.02	54.86	2.39
		mam	5.74	6.33	10.24	7.44	2.45
	gfpt2+anti-ifnα/β	ui	0.00	0.00	0.00	0.00	0.00
		ctrl	124.10	117.26	129.36	123.57	6.07
		cyto	108.26	135.28	128.91	124.15	14.12
		wt	62.74	57.96	66.29	62.33	4.18
		mito	72.18	76.95	68.09	72.41	4.43
		pex	47.29	53.26	58.14	52.90	5.43
		mam	83.02	86.35	90.14	86.50	3.56
	gfpt2+anti-ifnλ	ui	0.00	0.00	0.00	0.00	0.00
		ctrl	137.26	148.26	134.05	139.86	7.45
		cyto	130.48	136.26	140.75	135.83	5.15
		wt	72.48	78.29	73.05	74.61	3.20
		mito	86.29	80.17	91.24	85.90	5.55
		pex	78.26	86.25	83.04	82.52	4.02
		mam	3.74	5.17	9.31	6.07	2.89
ISG56	ctrl	ui	0.00	0.00	0.00	0.00	0.00
		ctrl	1.00	1.86	2.04	1.63	0.56
		cyto	0.83	1.75	2.85	1.81	1.01
		wt	17.08	23.66	27.15	22.63	5.11
		mito	18.28	22.85	24.95	22.03	3.41
		pex	12.75	16.99	18.09	15.94	2.82
		mam	14.27	8.26	11.74	11.42	3.02
	gfpt2	ui	0.00	0.00	0.00	0.00	0.00
		ctrl	0.95	1.57	2.41	1.64	0.73
		cyto	2.24	1.08	0.76	1.36	0.78
		wt	62.72	58.17	55.96	58.95	3.45
		mito	24.85	26.38	20.11	23.78	3.27
		pex	14.05	17.84	19.05	16.98	2.61
		mam	31.05	27.15	24.99	27.73	3.07
	gfpt2+anti-ifnα/β	ui	0.00	0.00	0.00	0.00	0.00
		ctrl	0.86	1.42	1.66	1.31	0.41
		cyto	1.48	1.25	1.33	1.35	0.12
		wt	8.09	9.11	5.04	7.41	2.12
		mito	4.75	2.06	7.26	4.69	2.60
		pex	14.75	17.89	20.75	17.80	3.00
		mam	5.27	9.35	4.11	6.24	2.75
	gfpt2+anti-ifnλ	ui	0.00	0.00	0.00	0.00	0.00
		ctrl	0.90	1.75	0.68	1.11	0.57
		cyto	0.86	0.77	1.93	1.19	0.65
		wt	12.74	9.05	7.44	9.74	2.72
		mito	3.11	4.78	1.86	3.25	1.47
		pex	3.26	2.18	5.97	3.80	1.95
		mam	33.17	28.02	23.14	28.11	5.02

Fig S10-a: VSV replication

			1	2	3	AVE	STDEV
Spleen	medium	Ctrl	0.00	0.00	0.00	0.00	0.00
		VSV	100.00	105.56	112.41	105.99	6.22
	6-AN	Ctrl	0.00	0.00	0.00	0.00	0.00
		VSV	426.38	415.26	397.26	412.97	14.69
Liver	medium	Ctrl	0.00	0.00	0.00	0.00	0.00
		VSV	100.00	108.96	102.47	103.81	4.63
	6-AN	Ctrl	0.00	0.00	0.00	0.00	0.00
		VSV	356.24	334.85	315.62	335.57	20.32

Fig S10-d: VSV replication

			1	2	3	AVE	STDEV
Spleen	medium	Ctrl	0.00	0.00	0.00	0.00	0.00
		VSV	100.00	113.02	104.36	105.79	6.63
	Aza	Ctrl	0.00	0.00	0.00	0.00	0.00
		VSV	395.24	352.24	376.55	374.68	21.56
Liver	medium	Ctrl	0.00	0.00	0.00	0.00	0.00
		VSV	100.00	107.25	111.36	106.20	5.75
	Aza	Ctrl	0.00	0.00	0.00	0.00	0.00
		VSV	341.25	330.74	322.41	331.47	9.44

Fig S10-b Spleen proinflammatory cytokines and IFN level

			1	2	3	AVE	STDEV
IFN-α	medium	Ctrl	5.26	11.75	3.17	6.73	4.47
		VSV	1857.96	2047.25	2165.24	2023.48	155.01
	6-AN	ctrl	15.04	7.26	6.25	9.52	4.81
		VSV	2247.96	1869.36	2172.81	2096.71	200.44
IFN-β	medium	Ctrl	13.72	9.02	18.29	13.68	4.64
		VSV	2817.14	3055.18	2674.25	2848.86	192.44
	6-AN	Ctrl	3.75	11.08	15.27	10.03	5.83
		VSV	2718.25	2965.36	2817.36	2833.66	124.36
IFN-λ	medium	Ctrl	5.37	9.36	14.27	9.67	4.46
		VSV	1475.26	1208.26	1287.36	1323.63	137.14
	6-AN	Ctrl	15.02	7.81	3.09	8.64	6.01
		VSV	82.36	142.36	127.26	117.33	31.21
IL-6	medium	Ctrl	12.75	2.74	21.96	12.48	9.61
		VSV	2075.36	2318.09	2275.69	2223.05	129.65
	6-AN	Ctrl	14.27	6.74	17.28	12.76	5.43
		VSV	628.36	875.26	439.28	647.63	218.63
IL1-β	medium	Ctrl	2.27	9.05	15.27	8.86	6.50
		VSV	1296.35	1079.26	1385.96	1253.86	157.70
	6-AN	Ctrl	26.35	20.14	17.25	21.25	4.65
		VSV	428.69	597.26	627.25	551.07	107.04

Fig S10-e Spleen proinflammatory cytokines and IFN level

			1	2	3	AVE	STDEV
IFN-α	medium	Ctrl	1.87	6.19	17.24	8.43	7.93
		VSV	2042.95	1876.26	1733.24	1884.15	155.01
	Aza	Ctrl	2.84	24.96	3.05	10.28	12.71
		VSV	741.25	596.35	924.56	754.05	164.48
IFN-β	medium	Ctrl	4.97	15.28	9.25	9.83	5.18
		VSV	2896.35	2641.85	2508.26	2682.15	197.16
	Aza	Ctrl	21.08	15.72	11.24	16.01	4.93
		VSV	785.96	669.24	814.27	756.49	76.88
IFN-λ	medium	Ctrl	2.79	6.77	11.42	6.99	4.32
		VSV	1217.95	1094.52	958.61	1090.36	129.72
	Aza	Ctrl	20.87	18.26	3.01	14.05	9.65
		VSV	1095.25	1189.36	1044.60	1109.74	73.46
IL-6	medium	Ctrl	1.57	6.11	15.36	7.68	7.03
		VSV	2396.47	2208.69	2497.06	2367.41	146.37
	Aza	Ctrl	21.57	3.96	7.28	10.94	9.36
		VSV	92.35	142.68	77.60	104.21	34.12
IL1-β	medium	Ctrl	1.77	6.18	15.44	7.80	6.98
		VSV	952.74	1347.05	1142.63	1147.47	197.20
	Aza	Ctrl	20.91	17.28	15.14	17.78	2.92
		VSV	86.29	104.75	57.26	82.77	23.94

Fig S10-c Liver proinflammatory cytokines and IFN level

			1	2	3	AVE	STDEV
IFN-α	medium	Ctrl	24.51	14.26	8.06	15.61	8.31
		VSV	974.24	815.27	804.21	864.57	95.13
	6-AN	ctrl	9.35	18.24	11.47	13.02	4.64
		VSV	887.92	982.04	718.94	862.97	133.31
IFN-β	medium	Ctrl	9.35	16.93	23.05	16.44	6.86
		VSV	1678.25	1892.36	1774.21	1781.61	107.25
	6-AN	Ctrl	21.05	15.24	7.15	14.48	6.98
		VSV	1705.96	1924.75	1977.25	1869.32	143.89
IFN-λ	medium	Ctrl	11.24	2.68	23.96	12.63	10.71
		VSV	847.26	715.96	671.06	744.76	91.56
	6-AN	Ctrl	3.55	14.05	23.06	13.55	9.76
		VSV	64.85	104.61	79.66	83.04	20.09
IL-6	medium	Ctrl	3.98	12.38	24.25	13.54	10.18
		VSV	1374.81	1456.25	1299.74	1376.93	78.28
	6-AN	Ctrl	6.97	15.24	19.04	13.75	6.17
		VSV	457.92	693.14	540.36	563.81	119.35
IL1-β	medium	Ctrl	15.02	23.80	7.92	15.58	7.95
		VSV	814.20	705.62	678.14	732.65	71.95
	6-AN	Ctrl	5.02	6.35	26.35	12.57	11.95
		VSV	215.02	201.33	149.20	188.52	34.73

Fig S10-f Liver proinflammatory cytokines and IFN level

			1	2	3	AVE	STDEV
IFN-α	medium	Ctrl	11.86	6.29	2.09	6.75	4.90
		VSV	1247.26	1089.36	1007.96	1114.86	121.67
	Aza	Ctrl	2.78	8.29	14.57	8.55	5.90
		VSV	425.69	381.05	304.85	370.53	61.10
IFN-β	medium	Ctrl	1.08	9.35	15.35	8.59	7.17
		VSV	1759.36	2014.26	1896.47	1890.03	127.57
	Aza	Ctrl	5.78	9.66	16.37	10.60	5.36
		VSV	715.04	663.21	508.19	628.81	107.63
IFN-λ	medium	Ctrl	2.78	8.29	13.22	8.10	5.22
		VSV	718.95	889.26	915.27	841.16	106.63
	Aza	Ctrl	4.78	9.25	19.35	11.13	7.46
		VSV	759.36	796.35	1052.34	869.35	159.55
IL-6	medium	Ctrl	8.29	16.38	24.85	16.51	8.28
		VSV	1463.05	1376.25	1078.29	1305.86	201.81
	Aza	Ctrl	5.92	9.67	18.29	11.29	6.34
		VSV	125.69	180.44	95.38	133.84	43.11
IL1-β	medium	Ctrl	7.35	9.35	19.25	11.98	6.37
		VSV	715.26	689.26	896.35	766.96	112.81
	Aza	Ctrl	17.84	22.48	3.45	14.59	9.92
		VSV	89.26	77.36	52.06	72.89	19.00

Fig s11a

(i):The samples were separated one gel, and the membrane was incubated with the indicated antibodies.

(ii)-(iii) :The same samples were separated two gels, and the membranes were cut and incubated with the indicated antibodies.

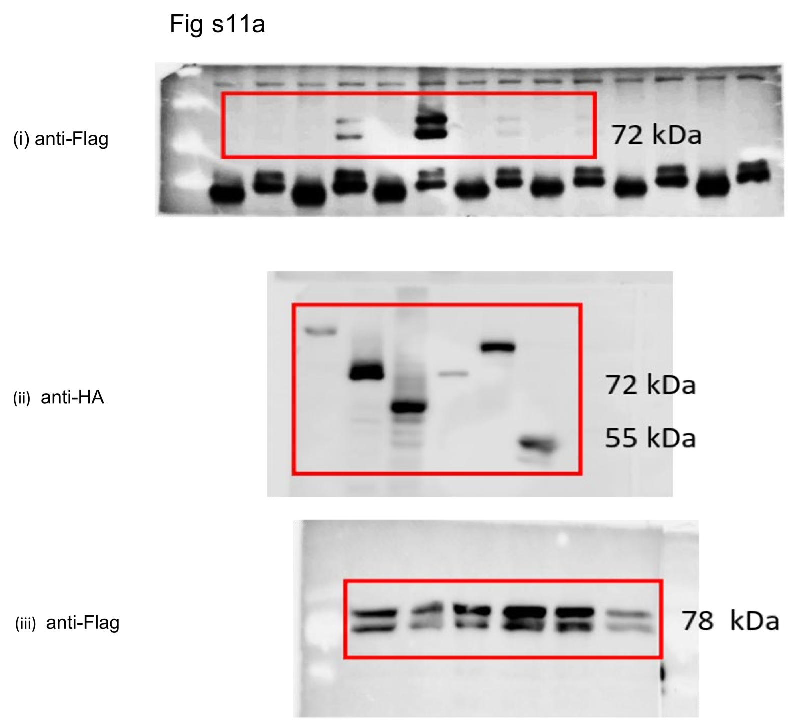

Fig s11b

(i) ,(ii): The same samples were separated two gels, and the membranes were cut and incubated with the indicated antibodies.

(iii)(iv),(v): Another set of the same samples were separated two gels, and the membranes were cut and incubated with the indicated antibodies

Fig s11c

(i) ,(ii): The same samples were separated two gels, and the membranes were cut and incubated with the indicated antibodies.

(iii)(iv),(v): Another set of the same samples were separated two gels, and the membranes were cut and incubated with the indicated antibodies

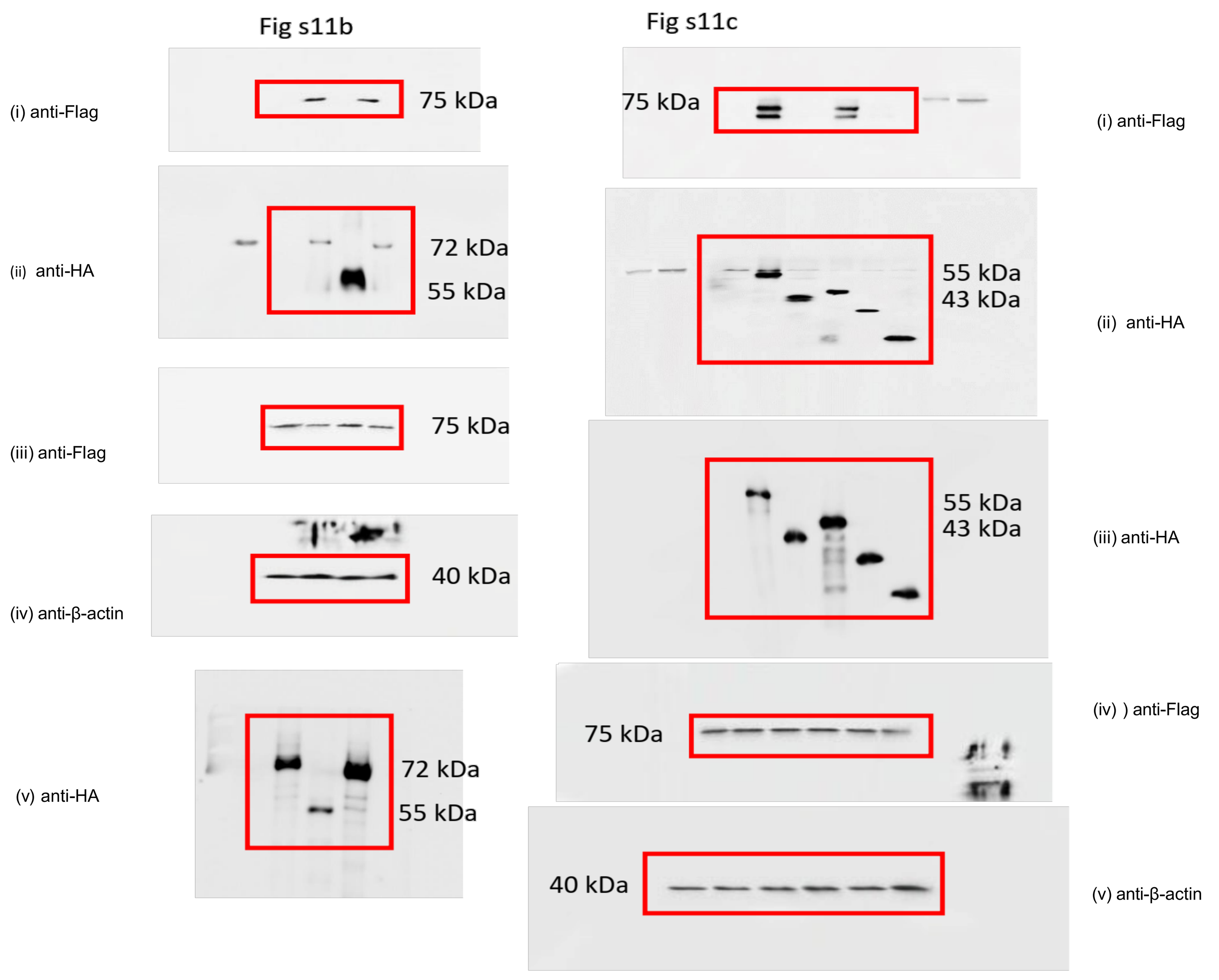

Fig s11d

(i) ,(ii): The same samples were separated on one gel, and the membrane was cut and incubated with the indicated antibodies.

(iii)(iv),(v): Another set of the same samples were separated on two gels, and the membranes were cut and incubated with the indicated antibodies.

Fig s11e

(i) ,(ii): The same samples were separated on one gel, and the membrane was cut and incubated with the indicated antibodies.

(iii)(iv),(v): Another set of the same samples were separated on two gels, and the membranes were cut and incubated with the indicated antibodies.

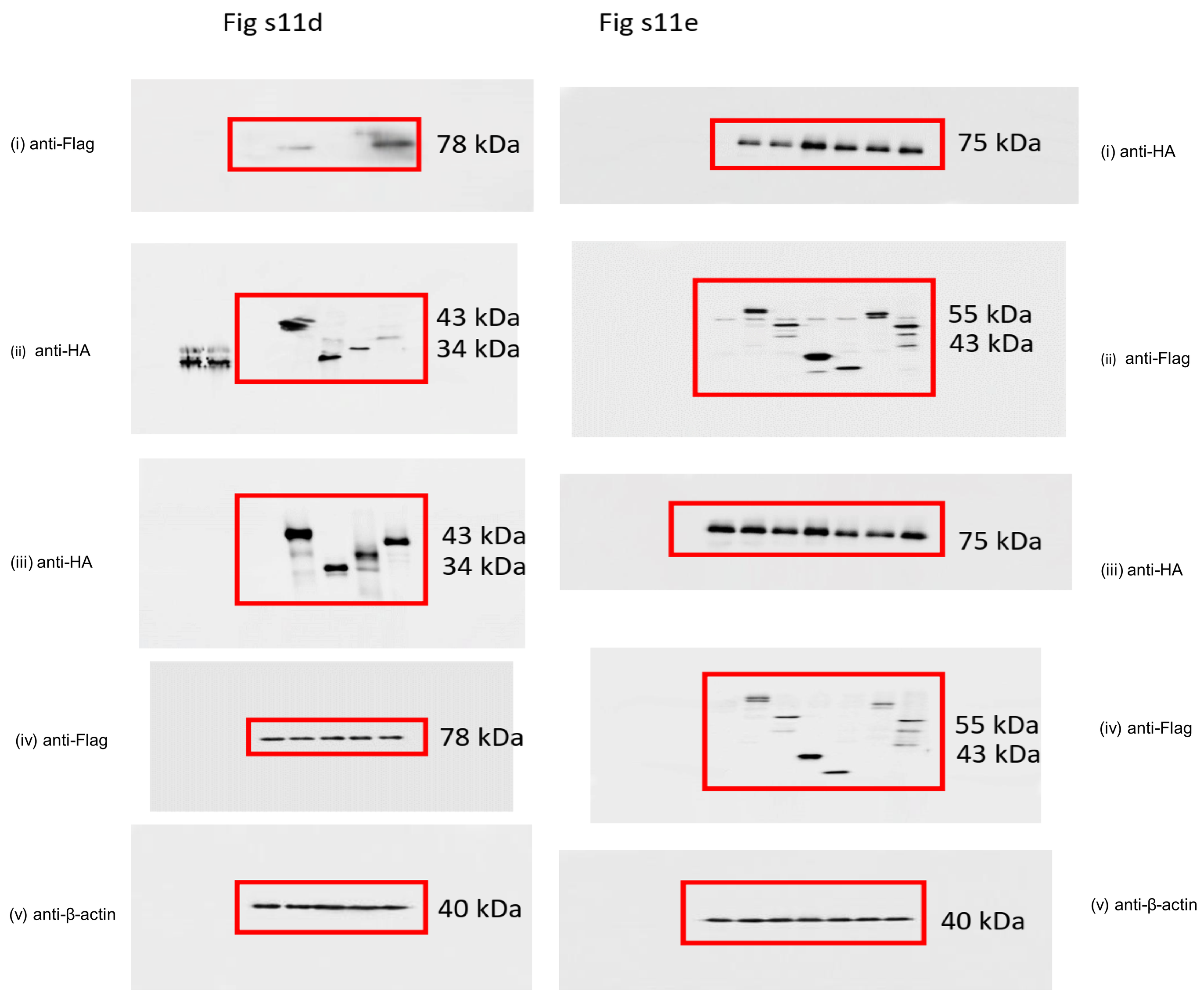

Fig s11f

(i) ,(ii): The same samples were separated on two gels, and the membranes were cut and incubated with the indicated antibodies.

(iii)(iv),(v): Another set of the same samples were separated on two gels, and the membranes were cut and incubated with the indicated antibodies.

Fig s11g

(i) ,(ii): The same samples were separated on two gels, and the membranes were cut and incubated with the indicated antibodies.

(iii)(iv),(v): Another set of the same samples were separated on two gels, and the membranes were cut and incubated with the indicated antibodies.

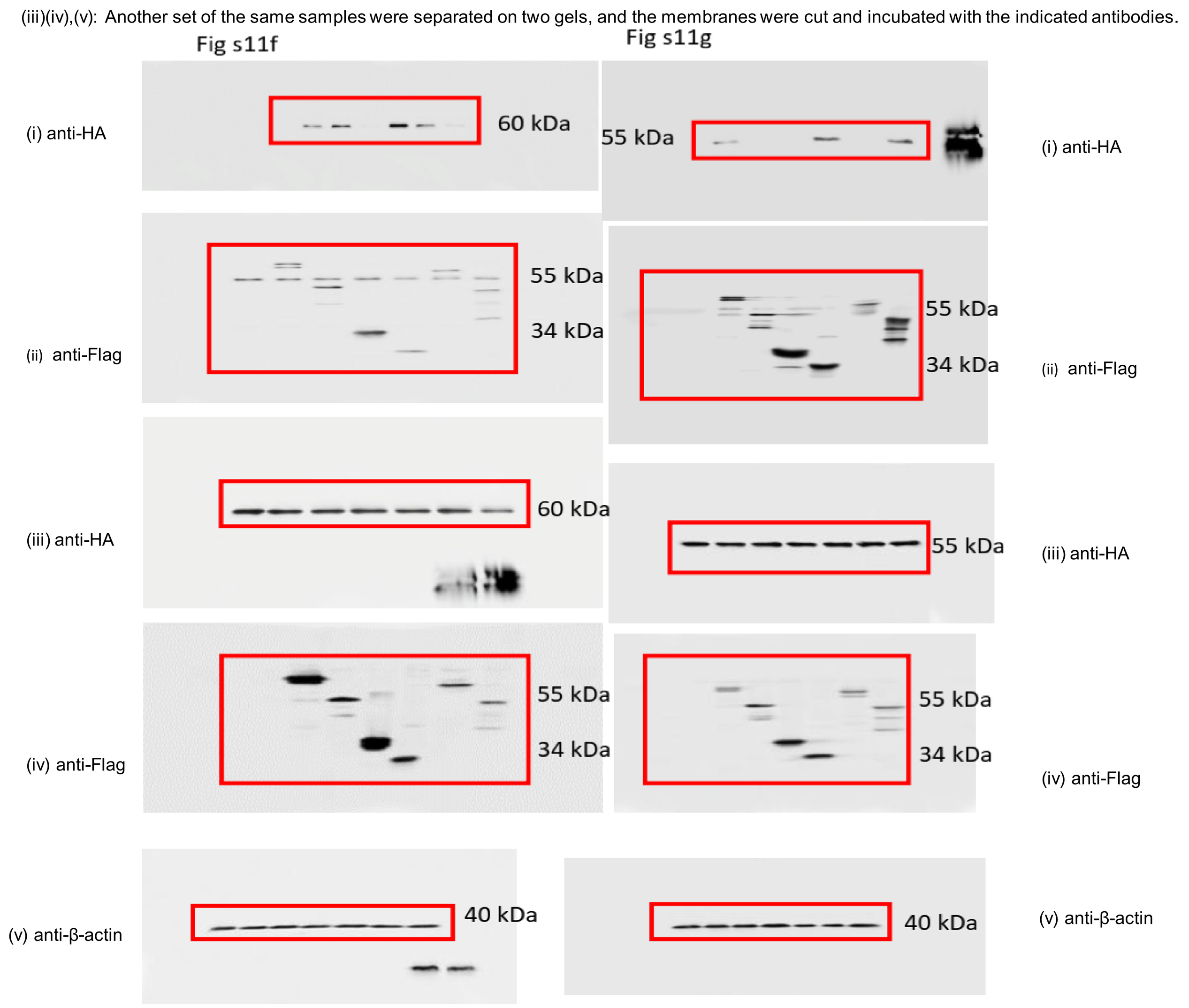

Fig s12a

(i)-(vii): The same samples were separated on five gels, and the membranes were cut and incubated with the indicated antibodies.

Fig s12b

(i)-(vii): The same samples were separated on five gels, and the membranes were cut and incubated with the indicated antibodies.

Fig s12a

(i) anti-p-IkBα

(ii) anti-IkBα

(iii) anti-p-IKKα/β

(iv) anti-IKKα/β

(v) anti-p-IRF3

(vi) anti-IRF3

(vii) anti-Tubulin

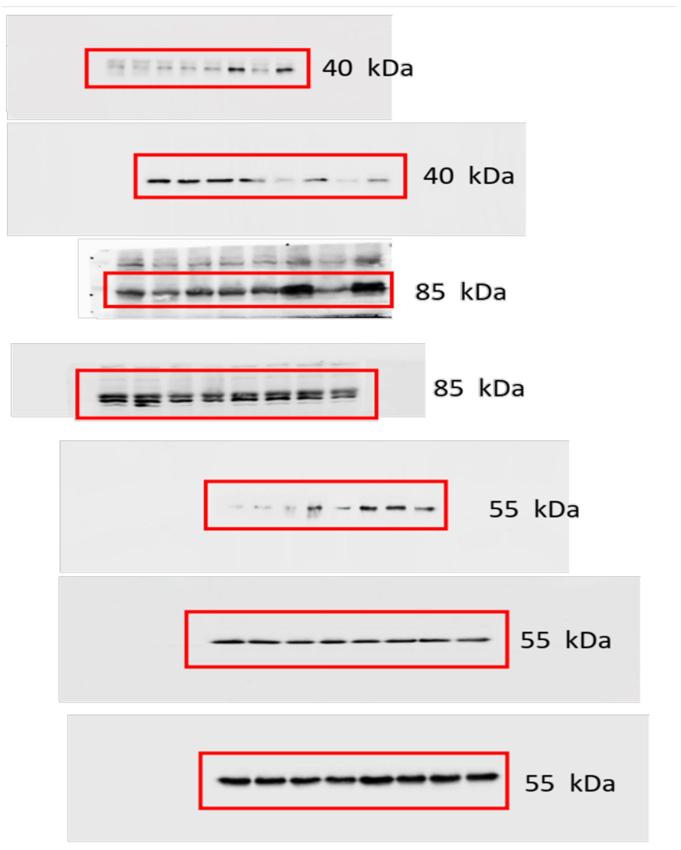

Fig s12b

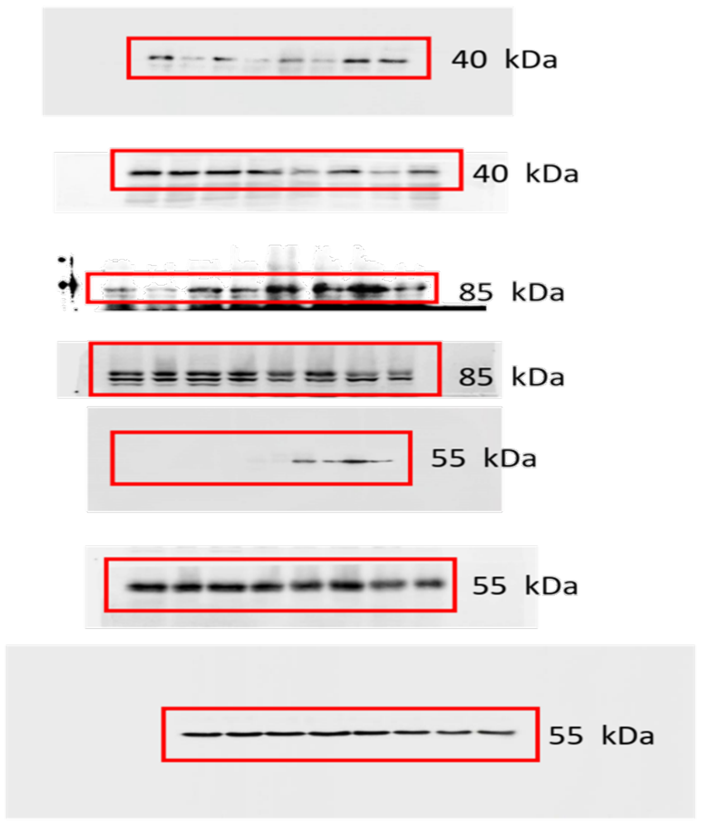

(i) anti-p-IkBα

(ii) anti-IkBα

(iii) anti-p-IKKα/β

(iv) anti-IKKα/β

(v) anti-p-IRF3

(vi) anti-IRF3

(vii) anti-Tubulin

Fig S12-c: NF-κB luciferase activity

	1	2	3	AVE	STDEV
ctrl	1.00	2.63	3.58	2.40	1.30
vsv	45.96	49.25	53.68	49.63	3.87
vsv+sh-gfpt2	8.96	7.26	16.02	10.75	4.65
ctrl	1.00	2.04	6.35	3.13	2.84
MAVS	23.84	27.92	31.25	27.67	3.71
mavs+sh-gfpt2	7.95	11.25	14.26	11.15	3.16

Fig S12-d: ISRE luciferase activity

	1	2	3	AVE	STDEV
ctrl	1.00	1.63	3.74	2.12	1.44
vsv	32.85	42.69	35.18	36.91	5.14
vsv+sh-gfpt2	5.96	7.15	2.99	5.37	2.14
ctrl	1.00	2.05	4.84	2.63	1.98
MAVS	17.26	20.14	13.85	17.08	3.15
mavs+sh-gfpt2	4.25	3.06	7.91	5.07	2.53

Fig S12-e: ISRE luciferase activity

	1	2	3	AVE	STDEV
ctrl	1.00	2.18	1.92	1.70	0.62
vsv	53.96	47.26	57.90	53.04	5.38
vsv+sh-gfpt	50.17	42.26	56.24	49.56	7.01
ctrl	1.00	3.07	2.11	2.06	1.04
MAVS	42.96	47.11	51.09	47.05	4.07
mavs+sh-gfp	40.17	42.68	56.35	46.40	8.71

Fig s13a

(i) ,(ii): The same samples were separated on two gels, and the membranes were cut and incubated with the indicated antibodies.

(iii)(iv),(v): Another set of the same samples were separated on two gels, and the membranes were cut and incubated with the indicated antibodies.

Fig s13b

(i) ,(ii): The same samples were separated on two gels, and the membranes were cut and incubated with the indicated antibodies.

Fig s13c

(i) ,(ii): The same samples were separated on two gels, and the membranes were cut and incubated with the indicated antibodies.

(iii)(iv),(v): Another set of the same samples were separated on two gels, and the membranes were cut and incubated with the indicated antibodies.

Fig s13d

(i) ,(ii): The same samples were separated on two gels, and the membranes were cut and incubated with the indicated antibodies.

(iii)(iv),(v): Another set of the same samples were separated on two gels, and the membranes were cut and incubated with the indicated antibodies.

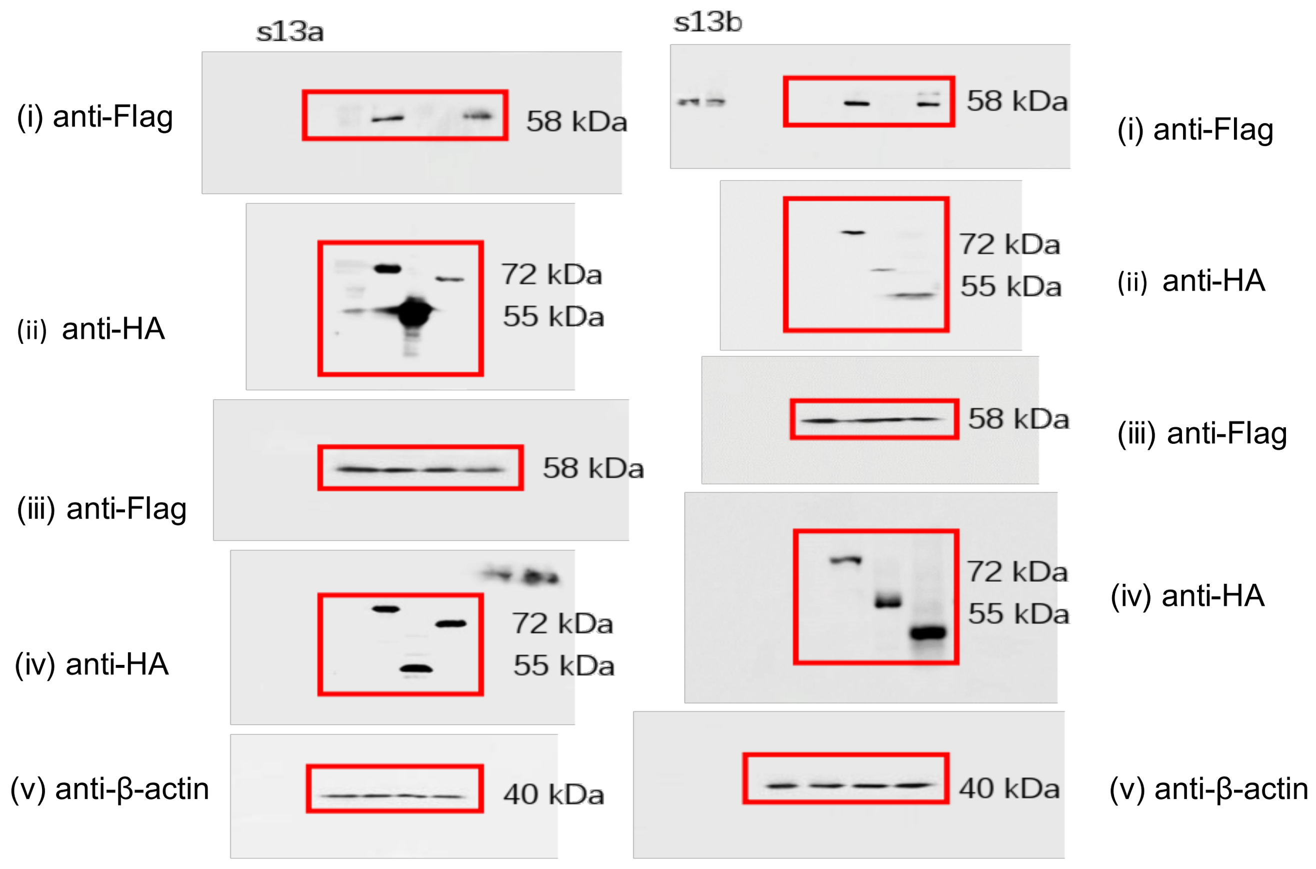

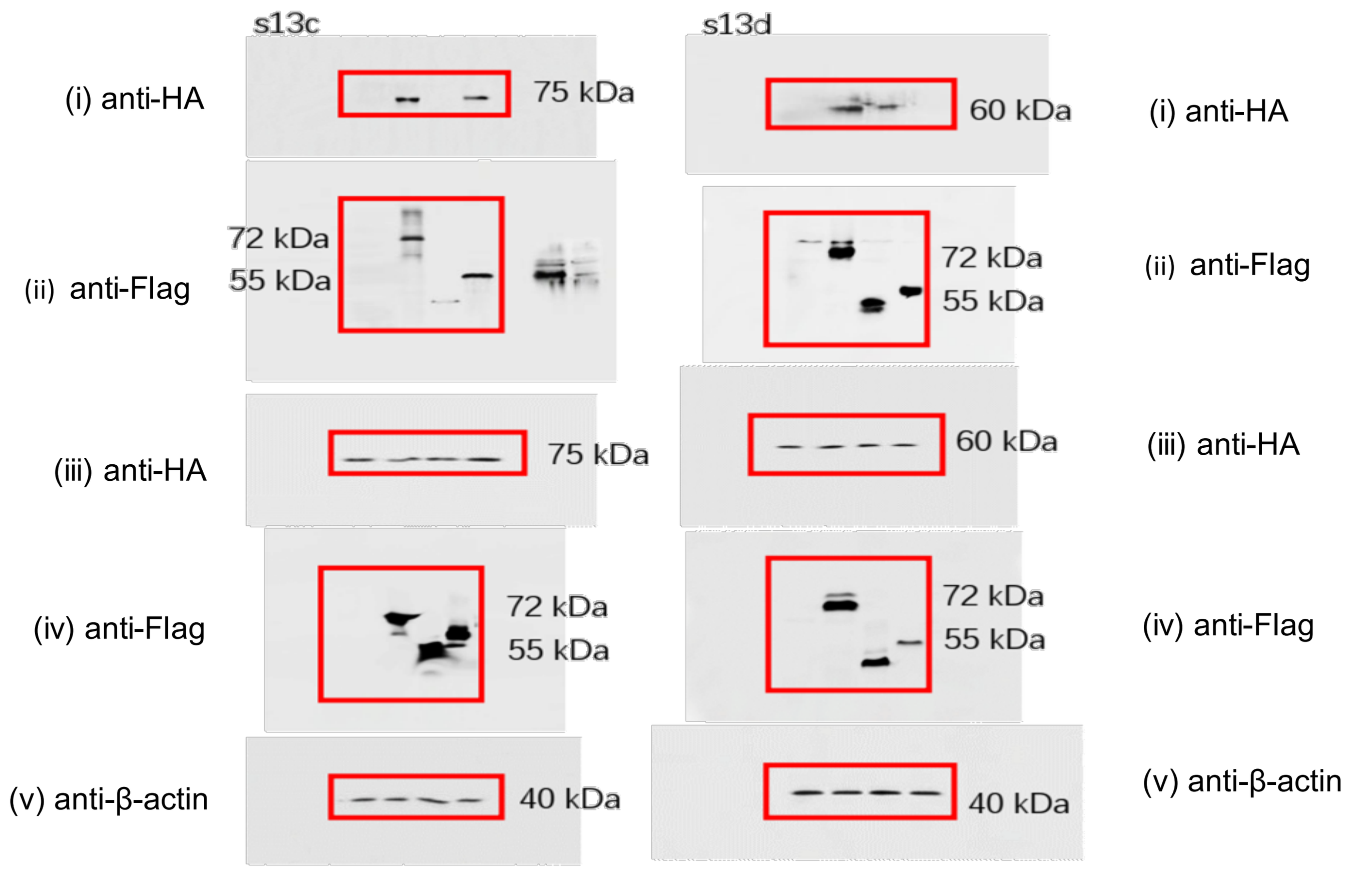

Fig S13-e: NF-κB luciferase activity

	1	2	3	AVE	STDEV
ctrl	1.00	5.24	2.78	3.01	2.13
vsv	62.24	63.14	58.29	61.22	2.58
vsv+sh-g6pd	38.36	34.27	46.33	39.65	6.13
ctrl	1.00	4.71	1.27	2.33	2.07
MAVS	25.36	21.07	29.35	25.26	4.14
mavs+sh-g6pd	14.29	10.25	16.38	13.64	3.12

Fig S13-f: ISRE luciferase activity

	1	2	3	AVE	STDEV
ctrl	1.00	3.04	5.71	3.25	2.36
vsv	35.29	41.25	33.96	36.83	3.88
vsv+sh-g6pd	22.04	28.92	16.33	22.43	6.30
ctrl	1.00	2.44	5.07	2.84	2.06
MAVS	15.27	19.36	13.24	15.96	3.12
mavs+sh-g6pd	12.36	9.85	6.33	9.51	3.03

Fig S13-g: ISRE luciferase activity

	1	2	3	AVE	STDEV
ctrl	1.00	2.38	4.15	2.51	1.58
vsv	52.14	47.96	44.25	48.12	3.95
vsv+sh-g6pd	13.68	3.05	6.41	7.71	5.43
ctrl	1.00	3.25	3.39	2.55	1.34
MAVS	43.26	33.74	38.91	38.64	4.77
mavs+sh-g6pd	3.25	4.79	9.95	6.00	3.51

Fig S14-b：MAVS-GFPT2 Quantitative colocalization									
	1	2	3	4	5	6	7	AVE	STDEV
Ctrl	0.12	0.07	0.31	0.17	0.44	0.23	0.19	0.22	0.12
VSV	0.92	0.99	0.75	0.83	0.90	0.87	0.89	0.88	0.07

Fig S14-d：MAVS-G6PD Quantitative colocalization									
	1	2	3	4	5	6	7	AVE	STDEV
Ctrl	0.22	0.56	0.59	0.23	0.37	0.21	0.14	0.33	0.18
VSV	0.93	0.75	0.66	0.59	0.81	0.76	0.72	0.75	0.11

Fig S15-a: mRNA level						
		1	2	3	AVE	STDEV
A549	ctrl	1.00	1.57	1.25	1.27	0.29
	TRAF6#1	0.26	0.14	0.06	0.15	0.10
	TRAF6#2	0.47	0.36	0.51	0.45	0.08
BMDMs	ctrl	1.00	1.51	1.63	1.38	0.33
	TRAF6#1	0.14	0.26	0.33	0.24	0.10
	TRAF6#2	0.31	0.42	0.22	0.32	0.10

Fig S15-c: mRNA level						
		1	2	3	AVE	STDEV
A549	ctrl	1.24	1.00	1.32	1.19	0.17
	IRF1#1	0.14	0.26	0.07	0.16	0.10
	IRF1#2	0.32	0.24	0.17	0.24	0.08
BMDMs	ctrl	1.00	1.46	1.59	1.35	0.31
	IRF1#1	0.13	0.17	0.32	0.21	0.10
	IRF1#2	0.46	0.29	0.25	0.33	0.11

Fig S15-e: metabolomics quantify metabolites						
		1	2	3	AVE	STDEV
Pyruvate	ctrl	1.00	1.05	1.49	1.18	0.27
	vsv	0.68	0.53	0.41	0.54	0.14
	vsv+sh-traf6	0.92	1.08	1.14	1.05	0.11
	vsv+sh-traf2	0.89	0.77	0.78	0.81	0.07
	vsv+sh-irf1	0.96	0.82	0.87	0.88	0.07
Lactate	ctrl	1.00	1.32	1.29	1.20	0.18
	vsv	0.62	0.33	0.29	0.41	0.18
	vsv+sh-traf6	1.02	1.17	0.96	1.05	0.11
	vsv+sh-traf2	0.89	0.75	0.61	0.75	0.14
	vsv+sh-irf1	0.73	0.88	0.79	0.80	0.08
Succinate	ctrl	1.00	1.05	1.19	1.08	0.10
	vsv	0.58	0.40	0.33	0.44	0.13
	vsv+sh-traf6	0.83	0.99	1.14	0.99	0.16
	vsv+sh-traf2	0.66	0.74	0.89	0.76	0.12
	vsv+sh-irf1	0.72	0.98	0.80	0.83	0.13

Fig S15-f: metabolomics quantify metabolites						
		1	2	3	AVE	STDEV
6PG	ctrl	1.00	1.21	1.26	1.16	0.14
	vsv	2.04	2.11	1.83	1.99	0.15
	vsv+sh-traf6	0.86	1.35	0.99	1.07	0.25
	vsv+sh-traf2	1.86	2.17	2.12	2.05	0.17
	vsv+sh-irf1	1.05	1.30	1.24	1.20	0.13
R5P	ctrl	1.00	1.09	1.33	1.14	0.17
	vsv	1.68	1.76	1.53	1.66	0.12
	vsv+sh-traf6	0.92	1.15	1.19	1.09	0.15
	vsv+sh-traf2	1.62	1.83	1.69	1.71	0.11
	vsv+sh-irf1	0.86	1.22	1.19	1.09	0.20
S7P	ctrl	1.00	1.07	1.11	1.06	0.06
	vsv	1.76	1.88	1.91	1.85	0.08
	vsv+sh-traf6	0.97	1.13	0.86	0.99	0.14
	vsv+sh-traf2	1.67	1.89	1.96	1.84	0.15
	vsv+sh-irf1	0.88	1.16	1.21	1.08	0.18
GSH	ctrl	1.00	1.34	1.04	1.13	0.19
	vsv	1.68	1.77	1.86	1.77	0.09
	vsv+sh-traf6	0.92	1.04	1.07	1.01	0.08
	vsv+sh-traf2	1.88	1.92	1.53	1.78	0.21
	vsv+sh-irf1	0.97	0.89	1.14	1.00	0.13

Fig S15-h: HK activity						
		1	2	3	AVE	STDEV
HK activity	ctrl	1.00	1.25	0.92	1.06	0.17
	vsv	1.68	1.83	1.72	1.74	0.08
	vsv+sh-traf6	0.83	0.92	1.04	0.93	0.11
	vsv+sh-traf2	1.96	2.12	2.09	2.06	0.09
	vsv+sh-irf1	2.11	1.96	1.92	2.00	0.10

Fig S15-j: G6PD activity						
		1	2	3	AVE	STDEV
G6PD	ctrl	1.00	0.92	1.04	0.99	0.06
	vsv	1.86	1.73	1.59	1.73	0.14
	vsv+sh-traf6	0.86	0.73	1.24	0.94	0.27
	vsv+sh-traf2	1.77	1.95	1.83	1.85	0.09
	vsv+sh-irf1	0.95	1.04	0.77	0.92	0.14

Fig S15-l: GFPT1 and GFPT2 mRNA levels						
		1	2	3	AVE	STDEV
GFPT1	ctrl	1.00	1.26	0.86	1.04	0.20
	vsv	1.52	1.06	1.75	1.44	0.35
	vsv+sh-traf6	0.86	1.42	1.76	1.35	0.45
	vsv+sh-traf2	0.87	1.54	1.93	1.45	0.54
	vsv+sh-irf1	0.86	0.76	1.72	1.11	0.53
GFPT2	ctrl	1.00	1.24	1.05	1.10	0.13
	vsv	7.36	9.35	5.22	7.31	2.07
	vsv+sh-traf6	2.14	2.06	1.75	1.98	0.21
	vsv+sh-traf2	1.11	2.85	0.96	1.64	1.05
	vsv+sh-irf1	8.36	9.25	6.17	7.93	1.59

Fig S15-b: mRNA level						
		1	2	3	AVE	STDEV
A549	ctrl	1.00	1.84	1.17	1.34	0.44
	TRAF2#1	0.14	0.06	0.21	0.14	0.08
	TRAF2#2	0.69	0.73	0.88	0.77	0.10
BMDMs	ctrl	1.00	1.27	1.82	1.36	0.42
	TRAF2#1	0.12	0.10	0.06	0.09	0.03
	TRAF2#2	0.23	0.34	0.16	0.24	0.09

Fig S15-d: mRNA level						
		1	2	3	AVE	STDEV
G6P	ctrl	1.00	1.06	1.24	1.10	0.12
	vsv	1.41	1.63	1.45	1.50	0.12
	vsv+sh-traf6	0.86	0.96	0.89	0.90	0.05
	vsv+sh-traf2	2.03	2.29	1.92	2.08	0.19
	vsv+sh-irf1	2.34	2.12	2.56	2.34	0.22
F6P	ctrl	1.00	1.14	1.36	1.17	0.18
	vsv	1.52	1.79	1.89	1.73	0.19
	vsv+sh-traf6	0.93	1.17	1.03	1.04	0.12
	vsv+sh-traf2	2.06	2.28	2.18	2.17	0.11
	vsv+sh-irf1	2.44	2.39	2.07	2.30	0.20
F1,6P	ctrl	1.00	1.18	1.02	1.07	0.10
	vsv	0.42	0.38	0.52	0.44	0.07
	vsv+sh-traf6	0.92	1.14	1.03	1.03	0.11
	vsv+sh-traf2	0.71	0.81	0.73	0.75	0.05
	vsv+sh-irf1	0.98	0.85	0.92	0.92	0.07
3PG	ctrl	1.00	1.35	1.17	1.17	0.18
	vsv	0.55	0.47	0.36	0.46	0.10
	vsv+sh-traf6	0.89	1.20	1.09	1.06	0.16
	vsv+sh-traf2	0.79	0.71	0.85	0.78	0.07
	vsv+sh-irf1	0.99	0.94	0.82	0.92	0.09

Fig S15-g: metabolomics quantify metabolites						
		1	2	3	AVE	STDEV
GlcN-6P	ctrl	1.00	1.19	1.30	1.16	0.15
	vsv	2.14	2.08	1.79	2.00	0.19
	vsv+sh-traf6	0.93	1.17	1.22	1.11	0.16
	vsv+sh-traf2	1.05	1.18	0.94	1.06	0.12
	vsv+sh-irf1	1.92	2.11	2.05	2.03	0.10
GlcNAc-6P	ctrl	1.00	1.21	1.13	1.11	0.11
	vsv	2.04	2.13	2.07	2.08	0.05
	vsv+sh-traf6	1.14	0.98	1.05	1.06	0.08
	vsv+sh-traf2	1.01	1.16	0.89	1.02	0.14
	vsv+sh-irf1	1.89	2.13	2.04	2.02	0.12
GlcNAc-1P	ctrl	1.14	1.06	1.35	1.18	0.15
	vsv	2.03	2.11	1.78	1.97	0.17
	vsv+sh-traf6	0.92	1.24	1.18	1.11	0.17
	vsv+sh-traf2	1.18	1.04	1.09	1.10	0.07
	vsv+sh-irf1	1.83	1.94	1.97	1.91	0.07
JDP-GlcNA	ctrl	1.00	1.18	1.12	1.10	0.09
	vsv	1.79	1.86	1.92	1.86	0.07
	vsv+sh-traf6	0.98	1.02	1.13	1.04	0.08
	vsv+sh-traf2	0.91	0.96	1.27	1.05	0.20
	vsv+sh-irf1	1.94	1.97	1.88	1.93	0.05

S15F ig-i: pyruvate and lactate levels						
		1	2	3	AVE	STDEV
Pyruvate	ctrl	1.00	0.86	1.14	1.00	0.14
	vsv	0.56	0.47	0.39	0.47	0.09
	vsv+sh-traf6	1.05	1.12	0.96	1.04	0.08
	vsv+sh-traf2	0.83	0.61	0.79	0.74	0.12
	vsv+sh-irf1	0.95	0.87	0.81	0.88	0.07
Lactate	ctrl	1.00	1.05	0.97	1.01	0.04
	vsv	0.53	0.44	0.41	0.46	0.06
	vsv+sh-traf6	1.05	1.09	1.13	1.09	0.04
	vsv+sh-traf2	0.79	0.98	0.70	0.82	0.14
	vsv+sh-irf1	0.84	0.89	0.86	0.86	0.03

Fig S15-k: NADPH and NADP+/NADPH ratio levels						
		1	2	3	AVE	STDEV
NAPDH	ctrl	1.00	0.85	0.77	0.87	0.12
	vsv	1.67	1.83	1.79	1.76	0.08
	vsv+sh-traf6	0.89	0.96	0.93	0.93	0.04
	vsv+sh-traf2	1.75	1.88	1.90	1.84	0.08
	vsv+sh-irf1	1.14	1.05	1.09	1.09	0.05
NAPD+/NAPDH	ctrl	1.00	0.98	1.16	1.05	0.10
	vsv	0.53	0.61	0.36	0.50	0.13
	vsv+sh-traf6	0.95	0.91	1.15	1.00	0.13
	vsv+sh-traf2	0.42	0.36	0.57	0.45	0.11
	vsv+sh-irf1	1.14	1.11	0.86	1.04	0.15

Fig S15-m: UDP-GlcNAc level						
		1	2	3	AVE	STDEV
UDP-GlcNAc	ctrl	1.00	1.05	0.93	0.99	0.06
	vsv	1.76	1.62	1.83	1.74	0.11
	vsv+sh-traf6	0.96	1.25	1.33	1.18	0.19
	vsv+sh-traf2	0.87	1.01	1.24	1.04	0.19
	vsv+sh-irf1	1.92	1.68	1.77	1.79	0.12

Fig S16-a: HK activity

		1	2	3	AVE	STDEV
ctrl	sh-ctrl	1.00	1.05	1.14	1.06	0.07
	sh-traf6	0.96	1.07	1.10	1.04	0.07
	sh-traf2	0.92	0.98	1.07	0.99	0.08
	sh-irf1	1.24	0.83	0.88	0.98	0.22
cyto	sh-ctrl	1.24	0.88	0.76	0.96	0.25
	sh-traf6	0.72	1.18	1.12	1.01	0.25
	sh-traf2	0.93	1.04	1.17	1.05	0.12
	sh-irf1	0.97	1.14	0.78	0.96	0.18
wt	sh-ctrl	2.14	1.86	1.80	1.93	0.18
	sh-traf6	0.86	1.22	0.83	0.97	0.22
	sh-traf2	1.93	1.86	2.11	1.97	0.13
	sh-irf1	2.12	1.99	1.76	1.96	0.18
mito	sh-ctrl	2.21	1.73	1.79	1.91	0.26
	sh-traf6	0.93	1.14	0.86	0.98	0.15
	sh-traf2	1.96	1.88	1.93	1.92	0.04
	sh-irf1	1.78	1.89	1.96	1.88	0.09
pex	sh-ctrl	0.96	1.14	1.27	1.12	0.16
	sh-traf6	1.12	1.09	0.86	1.02	0.14
	sh-traf2	1.17	0.86	1.03	1.02	0.16
	sh-irf1	0.96	1.05	1.07	1.03	0.06
mam	sh-ctrl	0.93	0.84	1.22	1.00	0.20
	sh-traf6	0.79	1.08	1.01	0.96	0.15
	sh-traf2	0.92	0.88	1.11	0.97	0.12
	sh-irf1	1.13	0.88	0.73	0.91	0.20

Fig S16-b: Pyruvate level

		1	2	3	AVE	STDEV
ctrl	sh-ctrl	1.00	1.05	1.14	1.06	0.07
	sh-traf6	0.86	0.93	1.26	1.02	0.21
	sh-traf2	1.28	0.80	0.89	0.99	0.26
	sh-irf1	0.92	1.01	1.08	1.00	0.08
cyto	sh-ctrl	0.93	0.91	0.86	0.90	0.04
	sh-traf6	1.14	0.75	0.81	0.90	0.21
	sh-traf2	0.84	0.96	0.99	0.93	0.08
	sh-irf1	1.04	1.08	0.87	1.00	0.11
wt	sh-ctrl	1.56	1.73	1.58	1.62	0.09
	sh-traf6	0.86	0.77	0.63	0.75	0.12
	sh-traf2	2.23	2.31	2.10	2.21	0.11
	sh-irf1	2.07	2.28	2.18	2.18	0.11
mito	sh-ctrl	1.75	1.66	1.81	1.74	0.08
	sh-traf6	0.86	1.14	1.09	1.03	0.15
	sh-traf2	1.83	1.96	1.78	1.86	0.09
	sh-irf1	2.14	1.76	1.70	1.87	0.24
pex	sh-ctrl	0.47	0.36	0.33	0.39	0.07
	sh-traf6	1.05	0.92	1.14	1.04	0.11
	sh-traf2	0.52	0.49	0.56	0.52	0.04
	sh-irf1	0.86	1.16	1.05	1.02	0.15
mam	sh-ctrl	0.39	0.41	0.30	0.37	0.06
	sh-traf6	1.22	0.86	1.04	1.04	0.18
	sh-traf2	1.01	0.92	1.19	1.04	0.14
	sh-irf1	0.53	0.57	0.31	0.47	0.14

Fig S16-c: Lactate level

		1	2	3	AVE	STDEV
ctrl	sh-ctrl	1.00	1.07	1.17	1.08	0.09
	sh-traf6	0.92	1.14	1.16	1.07	0.13
	sh-traf2	0.86	0.79	1.26	0.97	0.25
	sh-irf1	1.25	0.86	0.87	0.99	0.22
cyto	sh-ctrl	0.86	0.74	1.27	0.96	0.28
	sh-traf6	0.76	0.79	1.17	0.91	0.23
	sh-traf2	0.89	0.94	1.02	0.95	0.07
	sh-irf1	0.88	0.97	1.19	1.01	0.16
wt	sh-ctrl	1.83	1.61	1.77	1.74	0.11
	sh-traf6	0.86	0.79	0.93	0.86	0.07
	sh-traf2	2.15	2.21	2.32	2.23	0.09
	sh-irf1	2.30	2.17	2.11	2.19	0.10
mito	sh-ctrl	1.76	1.92	2.24	1.97	0.24
	sh-traf6	0.95	0.99	1.12	1.02	0.09
	sh-traf2	1.83	1.93	1.89	1.88	0.05
	sh-irf1	1.76	2.05	2.17	1.99	0.21
pex	sh-ctrl	0.35	0.47	0.51	0.44	0.08
	sh-traf6	1.12	0.95	0.91	0.99	0.11
	sh-traf2	0.52	0.55	0.43	0.50	0.06
	sh-irf1	0.93	1.01	0.87	0.94	0.07
mam	sh-ctrl	0.38	0.47	0.52	0.46	0.07
	sh-traf6	1.05	0.93	0.97	0.98	0.06
	sh-traf2	1.14	0.75	1.08	0.99	0.21
	sh-irf1	0.51	0.42	0.36	0.43	0.08

Fig S16-d: G6PD activity

		1	2	3	AVE	STDEV
ctrl	sh-ctrl	1.00	1.04	1.09	1.04	0.05
	sh-traf6	0.86	0.91	1.27	1.01	0.22
	sh-traf2	0.77	0.71	1.29	0.92	0.32
	sh-irf1	1.05	0.81	0.74	0.87	0.16
cyto	sh-ctrl	0.86	0.80	1.22	0.96	0.23
	sh-traf6	0.96	0.83	1.28	1.02	0.23
	sh-traf2	0.74	0.79	1.18	0.90	0.24
	sh-irf1	1.15	0.75	0.85	0.92	0.21
wt	sh-ctrl	1.89	1.93	2.14	1.99	0.13
	sh-traf6	0.96	0.84	0.83	0.88	0.07
	sh-traf2	2.24	1.79	1.82	1.95	0.25
	sh-irf1	1.13	1.05	0.93	1.04	0.10
mito	sh-ctrl	1.05	0.77	1.23	1.02	0.23
	sh-traf6	0.79	1.14	1.25	1.06	0.24
	sh-traf2	1.05	1.09	1.18	1.11	0.07
	sh-irf1	0.86	0.93	1.13	0.97	0.14
pex	sh-ctrl	1.75	1.83	1.96	1.85	0.11
	sh-traf6	0.93	0.84	1.18	0.98	0.18
	sh-traf2	2.01	1.71	1.80	1.84	0.15
	sh-irf1	1.06	0.93	0.87	0.95	0.10
mam	sh-ctrl	0.93	1.02	1.11	1.02	0.09
	sh-traf6	1.05	1.14	0.93	1.04	0.11
	sh-traf2	1.02	1.17	1.13	1.11	0.08
	sh-irf1	0.93	0.86	0.98	0.92	0.06

Fig S16-e: NADPH level

		1	2	3	AVE	STDEV
ctrl	sh-ctrl	1.00	1.07	1.12	1.06	0.06
	sh-traf6	0.77	0.84	1.23	0.95	0.25
	sh-traf2	1.16	0.73	0.78	0.89	0.24
	sh-irf1	1.27	0.73	0.71	0.90	0.32
cyto	sh-ctrl	0.96	0.91	0.87	0.91	0.05
	sh-traf6	1.05	1.09	0.83	0.99	0.14
	sh-traf2	0.63	1.24	1.12	1.00	0.32
	sh-irf1	0.86	0.96	1.17	1.00	0.16
wt	sh-ctrl	1.83	1.93	1.73	1.83	0.10
	sh-traf6	0.93	0.95	1.02	0.97	0.05
	sh-traf2	1.92	1.77	1.82	1.84	0.08
	sh-irf1	1.07	0.92	0.84	0.94	0.12
mito	sh-ctrl	1.04	0.93	1.17	1.05	0.12
	sh-traf6	0.86	1.12	0.93	0.97	0.13
	sh-traf2	0.96	1.15	1.25	1.12	0.15
	sh-irf1	0.71	0.75	1.31	0.92	0.34
pex	sh-ctrl	1.90	1.84	1.77	1.84	0.07
	sh-traf6	1.13	0.89	0.76	0.93	0.19
	sh-traf2	1.80	1.73	1.91	1.81	0.09
	sh-irf1	0.79	0.83	0.92	0.85	0.07
mam	sh-ctrl	0.78	1.23	1.14	1.05	0.24
	sh-traf6	0.73	0.71	1.29	0.91	0.33
	sh-traf2	1.14	1.02	0.93	1.03	0.11
	sh-irf1	0.94	1.01	1.15	1.03	0.11

Fig S16-f: NADP+/NADPH

		1	2	3	AVE	STDEV
ctrl	sh-ctrl	1.00	1.04	1.09	1.04	0.05
	sh-traf6	0.79	0.77	1.27	0.94	0.28
	sh-traf2	0.81	0.89	1.24	0.98	0.23
	sh-irf1	0.93	0.98	1.07	0.99	0.07
cyto	sh-ctrl	0.79	0.85	1.20	0.95	0.22
	sh-traf6	1.04	1.09	0.81	0.98	0.15
	sh-traf2	0.86	0.91	1.15	0.97	0.16
	sh-irf1	0.92	1.17	1.06	1.05	0.13
wt	sh-ctrl	0.32	0.28	0.39	0.33	0.06
	sh-traf6	1.06	0.99	1.17	1.07	0.09
	sh-traf2	0.51	0.30	0.57	0.46	0.14
	sh-irf1	1.25	0.86	0.89	1.00	0.22
mito	sh-ctrl	1.10	1.26	0.75	1.04	0.26
	sh-traf6	0.89	0.93	1.05	0.96	0.08
	sh-traf2	0.98	0.91	1.03	0.97	0.06
	sh-irf1	0.86	1.06	1.12	1.01	0.14
pex	sh-ctrl	0.29	0.56	0.51	0.45	0.14
	sh-traf6	1.03	1.15	1.09	1.09	0.06
	sh-traf2	0.37	0.59	0.44	0.47	0.11
	sh-irf1	1.10	1.18	0.93	1.07	0.13
mam	sh-ctrl	0.89	1.07	0.93	0.96	0.09
	sh-traf6	1.03	1.01	0.74	0.93	0.16
	sh-traf2	0.76	0.88	0.93	0.86	0.09
	sh-irf1	1.24	0.77	0.70	0.90	0.29

Fig S16-g: GFPT2 mRNA level

		1	2	3	AVE	STDEV
ctrl	sh-ctrl	1.00	2.04	3.12	2.05	1.06
	sh-traf6	3.86	2.93	3.17	3.32	0.48
	sh-traf2	2.99	4.05	4.02	3.69	0.60
	sh-irf1	3.87	4.07	3.14	3.69	0.49
cyto	sh-ctrl	2.84	3.93	2.07	2.95	0.93
	sh-traf6	2.81	1.86	1.21	1.96	0.80
	sh-traf2	3.85	2.15	3.03	3.01	0.85
	sh-irf1	2.01	2.24	2.73	2.33	0.37
wt	sh-ctrl	13.85	17.22	19.26	16.78	2.73
	sh-traf6	5.96	7.26	3.18	5.47	2.08
	sh-traf2	9.35	4.85	10.29	8.16	2.91
	sh-irf1	14.96	15.93	17.29	16.06	1.17
mito	sh-ctrl	2.75	3.86	4.02	3.54	0.69
	sh-traf6	1.28	4.26	2.85	2.80	1.49
	sh-traf2	4.15	2.36	3.96	3.49	0.98
	sh-irf1	4.15	3.65	1.82	3.21	1.23
pex	sh-ctrl	2.11	2.96	4.75	3.27	1.35
	sh-traf6	4.33	1.96	2.35	2.88	1.27
	sh-traf2	2.75	3.96	1.28	2.66	1.34
	sh-irf1	2.29	2.11	3.75	2.72	0.90
mam	sh-ctrl	18.20	20.74	16.35	18.43	2.20
	sh-traf6	9.74	11.25	6.37	9.12	2.50
	sh-traf2	8.19	5.69	11.24	8.37	2.78
	sh-irf1	16.93	17.95	21.35	18.74	2.31

Fig S16-h: UDP-GlcNAc level

		1	2	3	AVE	STDEV
ctrl	sh-ctrl	1.00	1.04	1.19	1.08	0.10
	sh-traf6	0.76	0.83	1.28	0.96	0.28
	sh-traf2	0.77	0.89	1.24	0.97	0.24
	sh-irf1	0.93	1.15	1.07	1.05	0.11
cyto	sh-ctrl	0.86	0.74	1.28	0.96	0.28
	sh-traf6	0.93	0.81	1.14	0.96	0.17
	sh-traf2	0.85	0.81	1.29	0.98	0.27
	sh-irf1	1.05	1.14	1.01	1.07	0.07
wt	sh-ctrl	2.11	1.86	1.76	1.91	0.18
	sh-traf6	0.93	0.87	1.04	0.95	0.09
	sh-traf2	0.89	0.96	1.04	0.96	0.08
	sh-irf1	2.07	1.92	1.88	1.96	0.10
mito	sh-ctrl	1.25	1.08	0.86	1.06	0.20
	sh-traf6	1.47	0.86	0.74	1.02	0.39
	sh-traf2	0.86	0.93	1.15	0.98	0.15
	sh-irf1	0.93	0.99	1.17	1.03	0.12
pex	sh-ctrl	0.89	1.05	0.94	0.96	0.08
	sh-traf6	0.79	0.88	1.21	0.96	0.22
	sh-traf2	1.26	0.86	0.81	0.98	0.25
	sh-irf1	0.86	0.93	1.22	1.00	0.19
mam	sh-ctrl	1.83	2.14	1.73	1.90	0.21
	sh-traf6	0.86	0.79	1.02	0.89	0.12
	sh-traf2	0.76	0.96	1.26	0.99	0.25
	sh-irf1	1.77	1.92	1.84	1.84	0.08
